# Complex Regional Pain Syndrome: Practical Diagnostic and Treatment Guidelines, 5th Edition

**DOI:** 10.1093/pm/pnac046

**Published:** 2022-06-10

**Authors:** R Norman Harden, Candida S McCabe, Andreas Goebel, Michael Massey, Tolga Suvar, Sharon Grieve, Stephen Bruehl

**Affiliations:** Departments of PM&R and Physical Therapy and Human Movement Sciences, Northwestern University; University of the West of England, Stapleton, Bristol, UK; Dorothy House Hospice, Bradford-on-Avon, Wilts, UK; Pain Research Institute, Faculty of Health and Life Science, University of Liverpool, Liverpool, UK; CentraCare Neurosciences Pain Center, CentraCare, St. Cloud, Minnesota, USA; Department of Anesthesiology and Pain Medicine, Rush University Medical Center, Chicago, Illinois, USA; Royal United Hospitals Bath NHS Foundation Trust, Bath, UK; Department of Anesthesiology, Vanderbilt University Medical Centers, Nashville, Tennessee, USA

**Keywords:** Complex Regional Pain Syndrome, CRPS, Reflex Sympathetic Dystrophy, RSD, Diagnostic Criteria, Treatment Guidelines

## Abstract

There have been some modest recent advancements in the research of Complex Regional Pain Syndrome, yet the amount and quality of the work in this complicated multifactorial disease remains low (with some notable exceptions; e.g., the recent work on the dorsal root ganglion stimulation). The semi-systematic (though in some cases narrative) approach to review is necessary so that we might treat our patients while waiting for “better research.” This semi-systematic review was conducted by experts in the field, (deliberately) some of whom are promising young researchers supplemented by the experience of “elder statesman” researchers, who all mention the system they have used to examine the literature. What we found is generally low- to medium-quality research with small numbers of subjects; however, there are some recent exceptions to this. The primary reason for this paucity of research is the fact that this is a rare disease, and it is very difficult to acquire a sufficient sample size for statistical significance using traditional statistical approaches. Several larger trials have failed, probably due to using the broad general diagnostic criteria (the “Budapest” criteria) in a multifactorial/multi-mechanism disease. Responsive subsets can often be identified in these larger trials, but not sufficient to achieve statistically significant results in the general diagnostic grouping. This being the case the authors have necessarily included data from less compelling protocols, including trials such as case series and even in some instances case reports/empirical information. In the humanitarian spirit of treating our often desperate patients with this rare syndrome, without great evidence, we must take what data we can find (as in this work) and tailor a treatment regime for each patient.

## Introduction

This is the fifth edition of the Diagnostic and Treatment Guidelines for Complex Regional Pain Syndrome (CRPS; also known as Reflex Sympathetic Dystrophy [RSD], causalgia). These guidelines have been sponsored by the Reflex Sympathetic Dystrophy Syndrome Association and are written by expert practitioners in each discipline that is traditionally utilized in the treatment of CRPS [1]. There is a fairly recent, excellent, rigorous systematic review of the treatment literature in CRPS [2] which confirmed there is only modest high-quality research in the area. Nonetheless, in this “evidence vacuum” we still have a responsibility to treat. Certainly, we must develop better evidence, but our patients cannot wait for that. Thus, although the authors of these practical guidelines all utilized a systematic approach to reviewing the available and relevant literature, they have also included less rigorous, preliminary research reports, often supplemented by extensive empirical experience. The authors perforce must also extrapolate from “related conditions” (e.g., neuropathy [3]). The research quality, clinical relevance and “state of the art” of diagnostic criteria or treatment modalities are discussed, sometimes in considerable detail. Where there have been no discernable updates in areas since the 4th edition, text from that has been kept, sometimes verbatim.

These guidelines are intended to serve as an aid to the informed practitioner. They are not intended to replace or supplant the clinician’s best judgment, experience, training and/or a careful consideration of the clinical context. Although every reasonable attempt has been made to minimize the bias of the authors, it must be recalled that, in context, all the experts are to a degree biased to “their” therapeutic approach.

Detailed sections are provided as a guide and informational source not only to the “expert” in CRPS therapy but also the primary practitioner who is interested. Levels of evidence are mentioned when appropriate (Table 1), so that the practitioner can better assess the modality under discussion and, if desired, to personally review the citations in detail. In the humanitarian spirit of making the most of all current thinking in the area, balanced by a careful case by case analysis of the risk/cost versus benefit analysis, we offer these “practical” guidelines.

**Table 1.** Levels of evidence (modified by consensus: used in prior versions and in this review [3])

Level 1: Meta-analysis or systematic reviews.
Level 2: One or more well-powered randomized, controlled trials, or statistically systematic validation criteria studies
Level 3: Retrospective studies, open label trials, small pilot studies.
Level 4: Anecdotes, case reports, clinical experience, empirical observations

## Diagnosis

Historically, Complex Regional Pain Syndrome (CRPS) has been referred to by many names, with Reflex Sympathetic Dystrophy (RSD) and Causalgia the best known. Both terms are sometimes still used inappropriately, and there are no validated diagnostic criteria for either. The historical evolution of terms and diagnostic criteria for CRPS is interesting and colorful but is beyond the scope of this review. Interested readers are referred to the prior version of this review for a more detailed history [3].

The label CRPS originated at an international conference held in Orlando, Florida, in 1994 [4, 5] that led to the first consensus-based diagnostic criteria for CRPS adopted by the International Association for the Study of Pain (IASP) [6]. These 1994 IASP criteria for CRPS were necessary and important, yet experience gained from developing and systematically improving diagnostic criteria for headache and psychiatric disorders highlighted the necessity of validating and modifying such preliminary consensus-based criteria through systematic validation research [7].To this end, a series of validation studies were conducted, leading ultimately to an empirically derived set of CRPS criteria (the so-called Budapest Criteria) that were adopted formally by the IASP committee on taxonomy as the new IASP criteria in 2012 (Table 2). The fact that the clinical presentation of CRPS (and its underlying mechanisms) can differ between patients and even within a patient over time made development of validated and clinically useful criteria somewhat more challenging. The results of these diagnostic validation studies are now briefly reviewed to detail the rationale for the format and content of the 2012 revised IASP criteria.

**Table 2.** Revised CRPS criteria adopted by the IASP in 2012 [3, 8]

General Features of the Syndrome:
CRPS is a syndrome characterized by a continuing (spontaneous and/or evoked) regional pain that is seemingly disproportionate in time or degree to the usual course of any known trauma or other lesion. The pain is regional (not in a specific nerve territory or dermatome) and usually has a distal predominance of abnormal sensory, motor, sudomotor, vasomotor and/or trophic findings. The syndrome shows variable progression over time.
*Clinical* Diagnostic Criteria for CRPS
1) Continuing pain, which is disproportionate to any inciting event
2) Must report at least one symptom in *three of the four* following categories:
*Sensory:* Reports of hyperalgesia and/or allodynia
*Vasomotor:* Reports of temperature asymmetry and/or skin color changes and/or skin color asymmetry
*Sudomotor/Edema:* Reports of edema and/or sweating changes and/or sweating asymmetry
*Motor/Trophic:* Reports of decreased range of motion and/or motor dysfunction (weakness, tremor, dystonia) and/or trophic changes (hair, nail, skin)
3) Must display at least one sign* at time of evaluation in *two or more* of the following categories:
*Sensory:* Evidence of hyperalgesia (to pinprick) and/or allodynia (to light touch and/or deep somatic pressure and/or joint movement)
*Vasomotor:* Evidence of temperature asymmetry and/or skin color changes and/or asymmetry
*Sudomotor/Edema:* Evidence of edema and/or sweating changes and/or sweating asymmetry
*Motor/Trophic:* Evidence of decreased range of motion and/or motor dysfunction (weakness, tremor, dystonia) and/or trophic changes (hair,nail, skin)
4) There is no other diagnosis that better explains the signs and symptoms

* A sign is counted only if it is observed at time of diagnosis.

###  

#### Validation Studies

The validity of the 1994 consensus-based IASP CRPS criteria was evaluated as part of a multisite study of patients meeting the 1994 CRPS criteria and a comparison group of non-CRPS neuropathic pain patients (level 2 evidence) [9, 10]. Signs and symptoms historically associated with CRPS were systematically-assessed in these patients using a standardized format. These clinical data were then subjected to a series of statistical analyses to address several questions regarding the 1994 criteria. The statistical approach used was based on one employed previously to validate headache diagnostic criteria [11–13] and psychiatric diagnostic criteria [14]. Detailed background on diagnostic validation methods and limitations can be found in Bruehl et al. [15].

In the first study, a statistical pattern recognition technique (principal component analysis) was used to identify distinct, statistically-derived subgroups of CRPS signs and symptoms (factors) as they occur in the clinical setting [9]. The format of the 1994 CRPS criteria implicitly assumed that signs and symptoms of CRPS cluster into two subgroups (pain/sensory and vasomotor/sudomotor/edema), an assumption that was not supported by the validation study [9]. Clinical features of CRPS actually clustered into four statistically-distinct subgroups (see Table 3 and discussion in Harden et al. [9]). The findings of this study had three important clinical implications. First, grouping of statistically distinct vasomotor and sudomotor/edema features into a single criterion in the 1994 IASP criteria likely led to poor specificity and overdiagnosis of CRPS [8–10]. Second, signs of motor dysfunction (e.g., dystonia, tremor) [16–18] and trophic features (e.g., changes in hair or nail growth) [19, 20] long believed to be part of the clinical syndrome comprised a distinct set of interrelated CRPS features that were entirely absent from the 1994 criteria. Finally, the observed patterns of CRPS signs versus symptoms suggested that while they were associated as expected, both likely provided non-redundant information that was potentially important for accurate diagnosis.

**Table 3.** Factors (and factor loadings) resulting from principal components analysis of diagnostic and associated signs and symptoms of CRPS, reused by permission from [9]

Factor 1	Factor 2	Factor 3	Factor 4
Hyperalgesia Signs (0.75)	Temperature Asymmetry Symptoms (0.68)	Edema Signs (0.69)	Decreased Range of Motion Signs (0.81)
“Hyperesthesia” Symptoms (0.78)	Color Change Signs (0.67)	Sweating Asymmetry Signs (0.62)	Decreased Range of Motion Symptoms (0.77)
Allodynic Signs (0.44)	Color Change Symptoms (0.52)	Edema Symptoms (0.61)	Motor Dysfunction Signs (0.77)
			Motor Dysfunction Symptoms (0.61)
			Trophic Symptoms (0.52)
			Trophic Signs (0.51)

Factor loadings can be interpreted as correlations between individual signs/symptoms and the overall factor on which they load. Reproduced from [*Harden, Bruehl, Galer,* et al. Complex Regional Pain Syndrome: are the IASP diagnostic criteria valid and sufficiently comprehensive? Pain 1999; 83(2): 211–9] doi: 10.1016/s0304-3959(99)00104–9 with permission from Wolters Kluwer Health Inc. Journal of the International Association for the Study of Pain.

The next validity study examined the accuracy with which the 1994 CRPS criteria were able to distinguish CRPS patients from non-CRPS neuropathic pain patients based on patterns of signs and symptoms [10]. This appeared to be a minimal requirement for clinical utility of the criteria. Although absence of a clear pathophysiological “gold standard” for CRPS diagnosis made design of this study more challenging, an approach was chosen based on methods used in developing evidence-based diagnostic criteria for other conditions with unclear pathophysiology (headache and psychiatric disorders) [8–10, 15]. The method chosen “stacked the deck” in favor of the 1994 criteria being able to discriminate accurately between the CRPS and non-CRPS neuropathic pain patients. Nonetheless, the results of a preliminary study and a subsequent larger study failed to support the validity of these criteria [10, 21]. In the larger more definitive study, diagnostic sensitivity of the 1994 IASP criteria (i.e., ability to detect CRPS when it is present) was found to be quite high as expected (0.98), but specificity (i.e., minimizing false positive diagnoses) was poor (0.36; i.e., worse than a coin flip), with a positive diagnosis of CRPS likely to be correct in as few as 40% of cases [10].

The results of the validity studies above prompted development and exploration of the potential utility of proposed revised CRPS criteria informed by these findings and designed to address the limitations identified with the 1994 IASP criteria. These proposed revised criteria grouped all CRPS features into one of the four statistically derived factors described above (pain/sensation, vasomotor, sudomotor/edema, motor/trophic; Table 3). Based on the findings of Harden et al. [9], these criteria also required the presence of a defined number of both objective signs *and* self-reported symptoms of CRPS for criteria to be met.

Using the same methodology described above, the sensitivity and specificity of the proposed revised criteria were directly compared to diagnostic discrimination using the 1994 IASP criteria [10]. Results showed that employing a decision rule requiring that at least *two of four sign* categories be positive and at least *three of four symptom* categories be positive for the diagnosis to be made resulted in a sensitivity of 0.85 (i.e., capturing most CRPS positive cases) and a specificity of 0.69, substantially improving on the specificity of 0.36 with the 1994 criteria (and better than most “objective” diagnostic tests). These proposed revised criteria and decision rules were reviewed at an international consensus meeting in Budapest, Hungary, in 2003, and a decision was made to conduct a second independent validation study to confirm the diagnostic findings above. Results of this re-validation study (level 2) [8] confirmed that the “Budapest criteria” had excellent sensitivity (0.99) and that they improved specificity substantially (0.68) over the older criteria. Based on these findings, the IASP Committee for Classification of Chronic Pain in 2012 formally adopted the revised criteria as part of the IASP pain taxonomy (Table 2). These criteria, with some clarifications, are in the process of being added to the International Classification of Diseases, 11th revision (ICD-11) [22].

It is important to recall that the “Budapest” criteria were designed and developed as a broad, inclusive and accessible screening type diagnostic criteria. CRPS is a disease of many different mechanisms usually presenting at different times in the course

#### CRPS Stages? CRPS Subtypes?

Is CRPS a uniform phenomenon across individuals, or are there distinct subtypes and/or stages of the syndrome? This issue of diagnostic heterogeneity, addressing whether or not patient presentations (i.e., the overall pattern of CRPS signs and symptoms) tend to be similar across individuals, may have significant implications for both prognosis and treatment. Historically, three progressive stages of CRPS have been cited as important in identifying and treating the syndrome (e.g., [23–25]), but empirical studies indicate that the existence of such sequential stages is clinical lore and is an unsubstantiated theory based on certain authors’ clinical experience rather than an outcome of specific scientific study (level 4). Statistical analysis (cluster analysis) to identify CRPS patient subgroups based on presence of similar patterns of clinical features has failed to support the traditional sequential staging of CRPS (level 2) [26, 27]. When patients are assigned into three subgroups based on similarity of CRPS features, pain duration is similar across the groups, unlike what would be expected if the traditional sequential stages of CRPS were valid [26, 27]. Results of the first such study [26], for example, identified distinct patient subgroups characterized by: (1) a relatively limited syndrome with vasomotor signs predominating, (2) a relatively limited syndrome with neuropathic pain/sensory abnormalities predominating, and (3) a florid CRPS syndrome with a wide array of CRPS features similar to “classic RSD” descriptions. Both studies addressing this issue found statistical evidence for this latter, more severe CRPS patient subgroup [26, 27].

In conclusion, these findings argue against the historical three *sequential* stages of CRPS [26, 28–30]. Nonetheless, lack of support for traditional sequential stages does not invalidate the concept of other CRPS subtypes that may evolve over time. One promising candidate, consistent with clinical observations, is the distinction between “warm CRPS” and “cold CRPS.” A large, international, prospective multi-site study tested whether distinct warm and cold CRPS subtypes could be identified solely using unbiased statistical pattern recognition (i.e., no a priori assumptions). Results of cluster analysis using automated cluster selection revealed a warm CRPS patient cluster characterized by a warm, red, dry and edematous extremity, and a distinct cold CRPS patient cluster characterized by a cold, blue, sweaty and less edematous extremity (level 2) [31]. Consistent with clinical observations, median CRPS duration was much shorter in the warm CRPS subtype (4.7 months) than in the cold CRPS subtype (20 months), with comparable pain intensity across these subtypes [31]. Although a warm presentation is by far the most common in early CRPS, a small subgroup of patients was noted who had CRPS of brief duration yet displayed a cold CRPS pattern, a group provisionally-labelled “primary cold CRPS” [31]. Further bearing on the issue of temporal sequencing of these subtypes, a score reflecting total number of inflammatory features was found to be significantly elevated at baseline in the warm subtype relative to the cold subtype, with these elevations significantly diminishing only in the warm CRPS subtype over a 3-month follow-up period. This pattern is what might be expected if cold CRPS reflected a relatively stable chronic non-inflammatory condition, whereas warm CRPS were more of an acute inflammatory state subject to a later transition in phenotype. Future application of comparable analytic methods to the complexities of CRPS may permit the identification of other discrete CRPS subgroups which may eventually permit more effective targeting of treatment interventions [32]. Although potentially important clinically, classification of “warm CRPS” vs “cold CRPS” in diagnosis remains at present an informal subtyping. There remains some hesitancy among experts to making this distinction a “formal” CRPS subtype until additional research is conducted, although there is agreement that clinicians should note whether a patient’s CRPS presentation is predominately warm or cold, given its possible implications for prognosis and treatment [22]. It is important to note that at this time there is no evidence to suggest that “subtyping” in any way obviates the need for interdisciplinary care, and subtyping (presumably reflecting different mechanisms) may be most relevant to predicting responses to individual interventions.

A recent IASP consensus meeting in Valencia, Spain, addressed another important CRPS diagnostic subtype issue [22]. In both the 1994 and 2012 versions of the IASP criteria, there was no CRPS subtype category to capture patients who had previously been diagnosed with CRPS, then improved sufficiently to no longer meet the full criteria but suffered from continued symptoms requiring ongoing care. This significant clinical issue prompted the proposal of a new formal CRPS subtype termed “CRPS with Remission of Some Features.” This subtype will be included in the new ICD-11 version of the CRPS criteria. It should be applied only to patients who were documented to meet full CRPS criteria at an earlier point in time but who currently do not display sufficient signs and symptoms to meet full criteria. Patients in this category are not necessarily improved with regards to pain intensity nor are they free of all CRPS-related signs and symptoms [22], and they may “relapse.” We empirically note the occasional patient who may fully meet diagnostic criteria one day and not the next. It is critical for legal and insurance reasons that temporarily not meeting criteria, for whatever reason, is not considered equal to a “cure” of the condition, particularly given the known lability of CRPS features.

A final CRPS subtyping issue is the distinction between CRPS-Type I (without “major nerve damage”) and CRPS-Type II (with “major nerve damage”; see Table 2). This is an historical distinction carried over into the 1994 IASP CRPS criteria based on the previously separate diagnostic categories of RSD (now CRPS-Type I) and Causalgia (now CRPS-Type II). At the time of the Budapest consensus group meeting, there was broad agreement that problems do exist with creating this division given the large overlap in clinical features between them (i.e., the primary diagnostic criteria are identical). The group was concerned that these divisions are dependent on nebulous definitions of what constitutes “major nerve damage,” and regarding what tests or criteria were necessary to make this distinction (e.g., electrodiagnosis, which is almost always too painful). Despite agreement that the CRPS-Type I vs. Type II distinction may neither be clinically significant nor affect the specific therapeutic method used, this distinction was retained by the Budapest group largely for historical reasons, and remains a formal subtype in the 2012 IASP CRPS criteria and the CRPS criteria to be included in the new ICD-11, pending more data.

#### Beyond Dichotomous Diagnosis: Assessment of CRPS Symptom Severity

Dichotomous diagnostic criteria (yes/no), while valuable both clinically and in research for consistent identification of CRPS as a syndrome, do not capture individual differences in the severity of CRPS. Tracking severity of CRPS symptoms (i.e., beyond pain intensity) is important for monitoring treatment-related changes in clinical care and is potentially valuable as a disease modification outcome in CRPS clinical trials. In 2010, the CRPS Severity Score (CSS) was developed to address this gap (level 2) [33]. The CSS deliberately uses the components of the 2012 IASP diagnostic criteria (and can be applied in any clinic, without special training or equipment; i.e., it is accessible) to create a continuous index of CRPS symptom severity. The final CSS version contains 16 elements (8 signs and 8 symptoms; e.g., allodynia/hyperalgesia, edema, skin temperature changes etc.) coded as present/absent based on the history and physical examination (possible score 0–16). In initial development work [33], CSS scores were associated significantly, as expected (i.e., higher scores were linked to worse patient status), with dichotomous diagnosis results, levels of pain intensity, distress, and functional impairments; and they discriminated well between CRPS and non-CRPS neuropathic pain patients. In a subsequent cross-validation sample (level 2) [34], CSS scores were relatively stable, as expected, in established chronic CRPS patients, with scores changing significantly more over time in acute CRPS patients undergoing initial treatment. Moreover, extent of changes in CSS scores over time were significantly associated with contemporaneous changes in pain intensity, fatigue, and functional impairments. Thus, the CSS appears to be valid and sensitive to change, a prerequisite for clinical utility. Available data indicate that a change of 5 or more CSS scale points reflects a clinically-significant change [34].

#### The Development of an International Core Data Set and Clinical Research Registry for CRPS

An International Research Consortium for CRPS (IRC) was established in 2015, which comprises a community of researchers in the field (https://www.crpsconsortium.org/). As part of its work, the IRC considered the current barriers to the conduct of collaborative, multicenter CRPS research studies in order to achieve adequate sample sizes for clinically meaningful studies. Currently, due to the low incidence rate of CRPS [35] and a heterogeneous patient population, research is mainly confined to small study populations. This presents methodological challenges associated with the synthesis of data, as a wide range of different outcome measures are used [36], and consequently advances in CRPS research are hindered. Clinical research registries, with agreed core data sets are widely used in healthcare, providing access to a large, standardized set of observational, retrospective data from across a wide geographical area [37, 38]. In CRPS, an international registry and core data set could be used to aid in the identification of risk factors that precipitate CRPS and potential CRPS subtypes, better understand the mechanisms driving the condition, and evaluate the effectiveness of treatments in clinical samples. Such a registry could provide researchers and clinicians with access to a novel, large and consistent set of CRPS outcome and demographic data, and lists with contact information for CRPS patients willing to participate in research. Access to such data would have the potential to improve health outcomes for the CRPS population worldwide.

To address this issue, in 2013, an international consortium of patients, researchers, clinicians and industry representatives was established with the long-term aim to establish agreement about a CRPS core data set for clinicians and researchers in the field, along with an international, clinical research registry for CRPS. The acronym COMPACT; “Core Outcome Measurement set for complex regional PAin syndrome Clinical sTudies,” was adopted to achieve this initiative. Membership was from 20 countries across Europe, the Americas, Asia, and Africa. Participation in the project work was via digital communications, teleconferences and international workshops. The COMPACT initiative was adopted by the IRC to facilitate international collaboration and the pooling of resources to improve the quality of CRPS-related studies (http://www.crpsconsortium.org). The Royal United Hospitals Bath NHS Foundation Trust, Bath, UK, is the lead center for this work.

**Figure 1.**
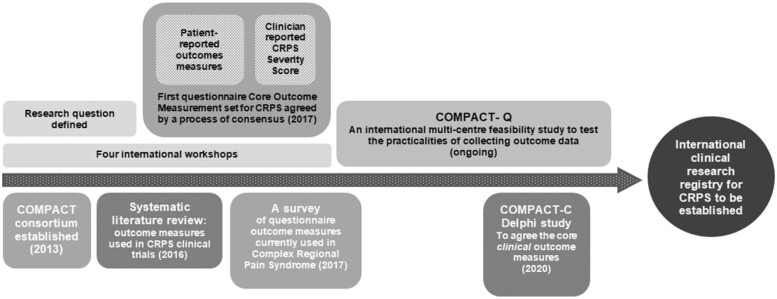
COMPACT process.

An iterative program of research, conducted by members of the COMPACT consortium, has informed the development of the long-term, international CRPS clinical research registry and core data set (Figure 1). This work is ongoing, and more detail is given below on each stage of the program to date.

##### A CRPS Core Data Set

A core outcome measurement set can be defined as a minimum set of standardized outcomes, which should be measured and reported in all research studies for a particular clinical condition [39]. The COMPACT core data set was developed in two stages and includes:

- Demographic data
- Patient-reported questionnaire outcome measures
- Clinician-reported outcomes/questionnaires.
- Clinical outcome measures (currently in development)

In the first stage, a core set of patient-reported and clinician-reported *questionnaire* outcome measures was agreed upon (Table 4). This work was informed by a systematic literature review utilizing PubMed of outcome measures used in CRPS clinical trials (level 1 evidence), and an online survey (level 4 evidence) of usage of outcome measures in CRPS clinical trials by healthcare professionals and academics [40]. Agreement on the final core set was achieved by means of consensus within international workshops [40]. The patient-reported questionnaire outcome measures captured the minimum domains needed to answer the consortium-agreed research question “What is the clinical presentation and course of CRPS and what factors influence it?” and comprised the following domains: pain, disease severity, participation and function, emotional and psychological function, sleep quality, self-efficacy, catastrophizing, and patient's global impression of change. One clinician-reported questionnaire outcome measure, namely, the CSS, was also included [34].

**Table 4.** COMPACT data set

Outcome Measure		Construct
Patient-reported	Demographic data	Date of birth, gender, CRPS affected limb, limb dominance prior to CRPS, CRPS duration and participation in employment/education/voluntary work.
	PROMIS-29^†^	PROMIS-29 Profile assesses 7 domains; physical function, pain interference and intensity, fatigue, depression, anxiety, sleep disturbance, and ability to participate in social roles and activities.
	Suicide ideation item	Assessed using a single PROMIS item
	Pain intensity numeric rating scale	The least and worst pain in the previous 24 hours captures the daily variability in its intensity.
	Short-form McGill Pain Questionnaire-2 (SF-MPQ-2)	Six neuropathic items capture pain quality
	Pain Catastrophizing Scale	Impact of catastrophizing on the pain experience
	EQ-5D-5L	Measurement of health state comprising mobility, self-care, usual activities, pain/discomfort, anxiety/depression
	Pain Self-efficacy Questionnaire	The respondent considers how confident they are performing each activity, while taking their pain into account
	CRPS symptom questions	Eight questions asking about CRPS symptoms and derived from the Budapest diagnostic criteria [8]
	Patient Global Impression of Change	This will be completed at follow-up only
Clinician-reported	CRPS Severity Score	The clinician records patient-reported CRPS symptoms and CRPS signs which are present on examination. Greater CRPS severity is indicated by a higher score [34]
Clinical measures	Currently in development.	To be defined

† PROMIS (Patient-Reported Outcomes Measurement Information System) is a National Institute of Health funded system, which provides validated patient reported outcome measures that can be used in a wide range of chronic conditions.

The second stage was comprised of a two stage e-Delphi study of clinicians and academics working internationally in the area of CRPS in order to agree on which (if any) *clinical* outcome measures should be included in the core data set. Results of the e-Delphi survey were presented to core members of the study team in a workshop held in Valencia, Spain, in September 2019. The group considered the feasibility and acceptability of each outcome in the final selected list, and whether an outcome should be “core” or optional. This work is in preparation for publication.

##### Development of a CRPS Clinical Research Registry

A multicenter, feasibility study was conducted over a two year period (2019–2021) to inform the design and delivery of the future CRPS international clinical research registry.

This study tested the feasibility and acceptability of collecting the COMPACT core outcome measurement set questionnaire data in an international population, and a bespoke electronic data capture system (ALEA) to collect and manage the COMPACT registry data, which was developed by the Clinical Informatics Research Unit (CIRU) at the University of Southampton, UK. Participants were recruited from research centers in Brazil, Israel, Japan, Switzerland, the USA, and the UK. Where applicable, COMPACT was translated using a “best practice” translation protocol [41]. The conduct and findings from this feasibility study are currently in preparation for publication.

It is anticipated the COMPACT international clinical research registry will be open for recruitment in 2022. In order to assess the future impact of this registry, and the CRPS core data set that underpins it, an online survey was conducted to provide a baseline assessment of the current use of questionnaire outcome measures by the international CRPS research community. Results demonstrated that researchers currently select from a broad range of different questionnaires outcome measures [36]. After the international registry is established, this survey will be repeated to establish what the global uptake is of the CRPS core data set.

Once the registry is “live,” information on how to contribute as a recruiting center, and how researchers can access these data for interrogation, will be found on the IRC website (https://www.crpsconsortium.org/). For the first time, the registry will provide researchers with access to an ever-increasing data set of standardized, CRPS-specific, outcomes for investigation.

## Interdisciplinary Management

The following interdisciplinary rehabilitation section focuses on the history, description and evidence for CRPS rehabilitation-based treatment. A systematic review of the evidence was conducted utilizing PubMed and MEDLINE. Search terms included “CRPS,” “Complex Regional Pain Syndrome,” “causalgia,” “reflex sympathetic dystrophy,” “rehabilitation,” “interdisciplinary management,” “occupational therapy,” “physical therapy,” “recreational therapy,” and “vocational therapy”. No time limit was applied to this search. Studies were selected based on the highest quality evidence available and relevance to CRPS rehabilitation. Also, anecdotal and practical information are included to assist the CRPS treatment practitioner.

**Figure 2.**
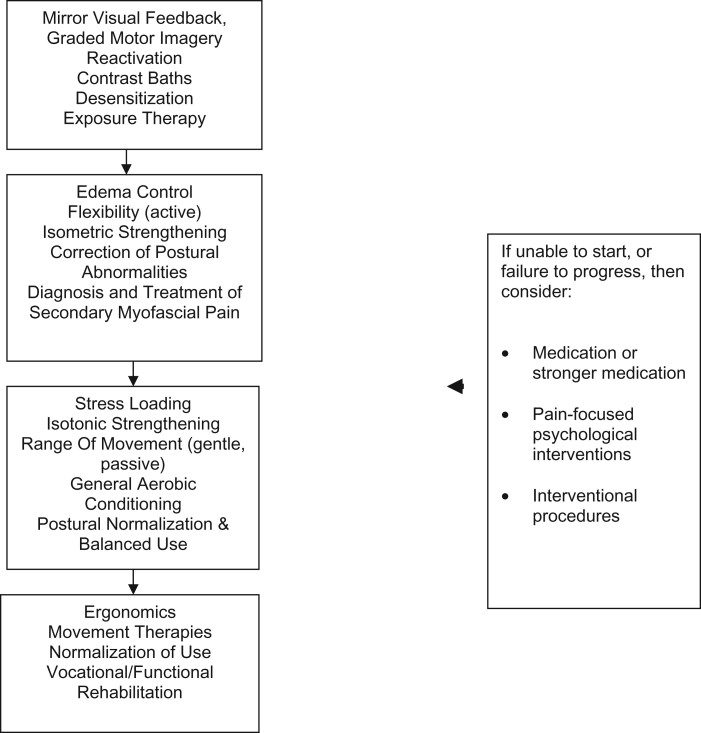
Malibu treatment algorithm (updated; modified by consensus) [3].

A Dahlem type (think-tank) conference was held in Malibu, California, in 1997 to generate consensus as to treatment guidelines for CRPS [1]. All treatments were focused on functional restoration (primarily the “reanimation of the affected part”); the use of drugs, blocks, and psychotherapy was reserved for patients failing to progress in the functional algorithm. Figure 2 portrays the Malibu CRPS treatment algorithm, updated to reflect some newer intervention approaches now available. Interdisciplinary/multidisciplinary pain management techniques emphasizing functional restoration are thought to be the most effective therapy for chronic pain, perhaps by resetting altered central processing and/or normalizing the distal environment (level 1) [42, 43]. The Malibu algorithm has been corroborated and empirically “validated” by frequent clinical use, although a proper controlled trial of such a clinical algorithm is not fiscally feasible.

The principle of functional restoration is based on a gradual and steady progression from activation of pre-sensorimotor cortices (i.e., motor imagery and visual tactile discrimination), to very gentle active movements such as progressive active Range of Motion (ROM), to weight bearing such as carrying light bags with the upper extremity or putting partial weight on the lower extremity in gait training (level 4) [44]. This progresses to movements that involve more active load bearing such as the scrub and carry techniques of Carlson (level 3) [45, 46]. Gradual desensitization to increasing sensory stimulus goes along with increased function. Desensitization strategies could include progressive stimulation with silk, progressing to other textures of cloth such as a towel, or contrast baths that progressively broaden the temperature difference between the two baths. It is thought that perhaps this gradual increase in normalized sensation tends to reset the “altered central processing” in the nervous system (level 3) [47]. It is important to manage edema in order to optimize range of motion and to encourage general aerobic activity throughout treatment (level 4) [48].

Another basic principle of these functional restoration guidelines is that if patients do not progress through the steps in “a reasonable time,” then other interventions will be progressively added to give the patient greater comfort or confidence so that they may proceed to the next level. For instance, if the allodynic pain is too great, a sympathetic and/or somatic block may give the patient a comfort window of opportunity to begin to entertain more aggressive therapy; or, if a patient has kinesiophobia [49, 50], cognitive behavioral techniques could be undertaken to demonstrate to the patient that movement does not necessarily lead to negative consequences. Sympathetic blocks, psychotherapy, and drugs should be used mainly in situations of failure to progress. However, if a patient presents with significant concomitant problems (e.g., severe depression or anxiety, pain too severe to engage in physical therapy) then certain drugs, blocks or psychotherapies are recommended from the outset (see below) [1].

### The Rationale for Functional Restoration

CRPS can be a very difficult condition to treat successfully. Not only is the syndrome bio-medically multifaceted, comprising both central and peripheral pathophysiology, but it also frequently contains psychosocial components that are additional pivotal diagnostic features (and thus, critical treatment targets). The array of possible patient presentations and the fact that the presentation often changes over time also complicate successful identification and treatment [26]. To further add to the clinical challenges of managing CRPS, the epidemiology and natural history of CRPS are only superficially known; evidence concerning CRPS treatment has developed slowly due in large part to the early vagaries of diagnosis (see above); and, moreover, research data—when they are available—are sometimes challenging to interpret [51]. Given these obstacles to diagnosis, treatment, and research, how is a specialist to embark on a path toward the successful treatment of such a complicated and partially understood condition? The only treatment methodology that can possibly successfully span these gaps in medical science is a systematic and orderly interdisciplinary approach [52]. Interdisciplinary treatment is defined (here) as a dedicated, coherent, coordinated, specially trained group of relevant professionals that meet regularly to plan, coordinate care, and adapt to treatment eventualities. Less desirable (but more accessible) is multidisciplinary treatment (a single practitioner coordinates all the various specialties).

Even the identification and measurement of the pain, the principal symptom of CRPS, is problematic. The defining characteristic (and critical diagnostic criterion) is “continuing pain that is disproportionate to any inciting event” [6]—pain deemed “disproportionate” [3] in intensity and duration according to the (subjective) opinion of the diagnosing physician. The problem is that different types of physicians may have distinct impressions of what level of pain is disproportionate. This necessary, yet biased, assessment of pain is confounded by the patient’s outlook; although pain is clearly a necessary and central component of a CRPS patient’s condition, its report is always a personal, private, and entirely subjective experience. Any number of factors can affect pain report, including culture, memory of past pain experiences, the meaning and context of the pain, personality type, affective state, and many other functional variables [53, 54]. Furthermore, pain report is behavioral: filling out a visual analog scale is a behavior, and any such behavior can be affected by a range of psychosocial/operant features. Unfortunately, only the subjective experience of pain is quantifiable. Limited by this subjectivity of both physician and patient, the most pragmatic assessment of pain must be based upon the patient’s complete context: biologic, psychologic, and sociologic. Obviously, the only treatment methodology that can treat all these aspects effectively is, again, the interdisciplinary approach.

It is critical to identify and aggressively treat all spheres of the pain experience. Obsessing with only the biomedical sphere often dooms the clinician and patient to failure, especially in chronic CRPS. Psychological factors and comorbidity are equally important, often modifiable, treatment targets in CRPS and can help ensure optimal outcomes (detailed below). The psychological spheres of the pain experience can now be identified through the many psychometric, quantified measures that have been created and that have demonstrated efficacy in psychological assessment [54–56].

Psychological features are sometimes critically important diagnostic components to identify and aggressively treat. Subjective but quantifiable psychometric scores are also often employed as secondary outcomes in research. CRPS is not a psychological disorder, however, and it is therefore usually illogical to designate psychometric outcomes as primary benchmarks of improvement in CRPS treatment. Thus, solely treating psychological aspects of a patient’s CRPS is also doomed to fail. Both pain intensity and the psychological sequelae/co-morbidities of pain are recognized, fundamental elements in understanding the whole patient, yet the subjective character of these elements and their measurement deem them less suitable for research or for interpreting clinical outcomes. Applying interventions solely as a means of trying to reduce pain ratings is a strategy that is equally doomed to fail. More objective clinical benchmarks and outcomes should be identified if possible—standards upon which clinical decisions may be made and success may be measured. Thus, ideally, treatment of CRPS should rely upon an intuitive, measurable, and step-wise functional restoration algorithm as the pivotal feature of treatment of CRPS [1, 26, 57].

Functional restoration has historically and empirically been considered a critical and necessary component of interdisciplinary pain management programs for CRPS. This contention has been codified by two large international consensus-building conferences [1, 58]. Baron and Wasner concluded that physiotherapy is “of utmost importance” [59], and Birklein et al. argued that rehabilitation techniques should always be employed for the “obvious reasons” outlined in these manuscripts [60, 61]. In a Dutch multidisciplinary evidence-based guideline for treatment of CRPS [2], physical therapy is reported to have beneficial effect with regard to functional restoration and ability to cope with the complaint, and therefore should form a part of the standard treatment for CRPS. Furthermore, the Initiative on Methods, Measurement, and Pain Assessment in Clinical Trials (IMMPACT) has concluded that physical functioning is a “core domain” in the assessment of pain treatment efficacy, second only to pain assessment [62, 63].

Functional restoration emphasizes physical activity (“reanimation”), desensitization and normalization of sympathetic tone in the affected limb, and involves a steady progression from the most gentle, least invasive interventions, to the ideal of complete rehabilitation (such as return to work/studies) in all aspects of the patient’s life (see Figure 2). Although the benefits of functional restoration may be obvious to experienced clinicians, the evidence required to buttress these empirical impressions remains to be collected. The systematically collected research data needed to determine which aspects of treatment demonstrate efficacy, which specific components of a functional restoration program yield positive outcomes, as well as which modalities should be delivered, when, and for how long, are currently unavailable [58, 64].

### Evidence

The data supporting functional restoration and reanimation in CRPS management are currently modest but credible. It is important to note that in a 1997 meta-analysis, Kingery noted that “CRPS trials tended to use less subjects and were less likely to use placebo controls, double blinding, or perform statistical tests for differences in outcome measures” (than neuropathic pain) (level 1) [51]. Early, uncontrolled work by several investigators focused on preliminary concepts of quantifying different facets of function and biometrics in “Reflex Sympathetic Dystrophy” (RSD, aka CRPS I) [19, 65–68]. In a pivotal 1988 paper, Davidoff et al. conducted a prospective uncontrolled study in RSD that determined three key concepts: that objective functional components and biometric data could be quantified longitudinally, that these components were reactive enough to display change over time (in response to a functional restoration-based interdisciplinary program), and that they were associated with improvements in subjective outcomes (decreased pain) (level 3) [57]. These initial studies supplied the primary rationale for a reliance on functional measures as the basis for assessing success in the treatment of RSD/CRPS. In an open label sample of musculoskeletal pain patients, Baker et al. convincingly illustrated the value of quasi-quantitative and psychometric measures in estimating functional outcome (level 3) (although this may not generalize to CRPS completely) [69].

Various uncontrolled studies suggest that CRPS patients benefited from certain physiotherapeutic modalities, including stress loading and isometric techniques (level 4) [45]. Oerlemans et al. conducted a prospective controlled study of 135 CRPS patients with pain located in an upper extremity, and she reported that both physical therapy (PT) and occupational therapy (OT) proved valuable in managing pain, restoring mobility, and reducing impairment (level 2) [64, 70]. Daly and Bialocerkowski reported in a well-performed meta-analysis (level 1) good quality evidence that pain management physical therapy combined with medical management is more effective than control therapy, based largely on the Oerlemans et al. study [71]. In their prospective assessment of 145 patients, Birklein et al. found that pain was notably less for patients undergoing PT (level 3) [60]. In another study of 28 children meeting the IASP criteria for CRPS, 92% reduced or eliminated their pain after receiving exercise therapy (level 3) [72].

Both functional restoration and reanimation may have beneficial effects for the CRPS patient. Limb immobilization is recognized as a possible cause and/or perpetuating factor in many cases of CRPS [6]. Motor abnormalities (dys-coordination, dystonia, weakness, and tremor) in CRPS [73, 74] are also one of the diagnostic features in the new 2012 IASP diagnostic criteria. Additionally, the role of pathological involvement of local muscle spasm, reactive bracing, and disuse in the face of severe pain in relation to the perpetuation of the syndrome should not be misjudged or underestimated; these secondary phenomena can cause severe pain and disability, and all must be assessed and actively treated in an interdisciplinary-based functional restoration or “normalization” program.

Normalized movement may also be a key aim in avoiding or reversing some of the more understated, higher central changes linked with the syndrome, usually categorized under the rubric of “altered central processing” and “neglect” [73]. Moseley et al. expands on this hypothesis and suggests that the elements of CRPS indicate a central mismatch of afferent input and central representation (level 3) [75], and that graded motor imagery may “repair this dynamic central mismatch” [74]. In their meta-analysis, Daly and Bialocerkowski found good to very good evidence for the efficacy of graded motor imagery physical therapy in combination with medical management for upper and lower extremity CRPS, resulting in clinically relevant and long-lasting pain reduction (level 2) [71]. In a novel experiment using mirrors, sensory mismatch was demonstrated to produce sensory disturbances in normal volunteers [76] and has also been employed in a controlled pilot to successfully treat CRPS I (level 3) [77]. These positive effects of mirror therapy were confirmed in a randomized controlled trial with 48 stroke patients with CRPS (level 2) [78], finding significant reduction of patient and enhanced upper limb motor function compared to control treatment. See Smart, Wand, and O’Connell for a review [79].

In addition to these findings, positive results have been described in small sample, open label studies aimed at sensorimotor “retuning” (desensitization techniques combined with motor tasks), and proprioceptive feedback enhancement (using vibratory stimulation). These studies have shown reduction in pain and normalization of proprioception in CRPS patients (level 3) [80–82]. Desensitization resulted in normalization of cortical organization in CRPS patients in the study by Pleger et al., apparent restoring normal brain function in the SI and S2 regions of the sensory cortex [80]. These interesting and mechanistically-relevant studies provide a theoretical rationale for the more pedestrian physical and occupational therapeutic methodologies of simple functional restoration, graded exposure, and ordered normalization of movement patterns.

In addition to the reversal of immobilization, the elimination of operantly-learned movement phobia (“kinesiophobia”) presented by so many of our patients may supply another rationale for establishing “functional restoration” as a fundamental requirement, and provide a primary role for co-treatment using physical and cognitive behavioral psychological therapies. Evidence regarding exposure-based therapies that target kinesiophobia is summarized in the psychological therapy section below. While additional outcome data are needed, clinical experience has indicated that approaches that target kinesiophobia produce substantial positive reinforcement in our clinics (level 4). The benefit of a pragmatic integration of graded, supported “exposure” to normalized movement into functional restoration programs for CRPS may be self-evident but requires further research.

The traumas usually identified in the etiology of CRPS most likely begin with peripheral nociceptive stimulation, and this “nociceptive barrage” may eventually create and sustain the central sensitization that is indicated by the sensory/psychophysical changes associated with the syndrome. It is hypothesized that normalization of activity will adjust and normalize the afferent input and its processing; for example, an increased functional input on large fiber tracts may modulate or partly obstruct the activity on small fiber tracts, according to Melzack’s original gate theory of pain [83]. Blood flow and nutrition to the area may be improved by local activity in the affected part, and processes such as osteopenia (i.e., “Sudeck’s atrophy”) may also be reversed by reactivation. Animal research supports this concept, as does human research on the effects of experimental limb immobilization. Healthy individuals experiencing casting of a limb for 28 days developed several clinical features associated with CRPS (e.g., skin temperature changes, hyperalgesia, altered hair growth, movement-induced pain) [84]. Such findings have been corroborated in Guo et al.’s rat research in which casting led to autonomic disturbance and allodynia [85]. Intuitively, normalizing function in CRPS patients should reverse changes wrought by immobilization

These studies all support the traditional functional/physiotherapeutic rationale, although there is currently no level 1 or 2 evidence specifically for interdisciplinary treatment for CRPS. An observational cohort study of 49 patients with CRPS participating in a comprehensive interdisciplinary pain program demonstrated short-term functional improvements. Further, the majority of worker’s compensation patients within this cohort (14 out of 16) returned to work (level 3) [86]. It is important to note two meta-analyses (level 1) that have shown that an interdisciplinary approach improves symptoms in patients with chronic pain: Flor et al. (which included “RSD” among other diagnoses) and Guzmán et al. [42, 43]. More details are presented in the sections below. While interdisciplinary management has potential for CRPS benefits based on the information summarized above, it remains to be definitively proven that this approach is as effective in CRPS as in other chronic pain conditions. Two recent studies suggest that altered brain function in CRPS patients (greater susceptibility to altered body representations, less efficient tactile-spatial learning) could potentially be a barrier to optimal response to functional interventions that are the core of interdisciplinary management (level 3) [87, 88]. At present, this possibility remains speculation.

### Principles of Functional Restoration

####  

##### Concerns with the Malibu Guidelines

In order to expedite reanimation and normalization of use of the affected extremity, functional restoration should efficiently supply a range of interventional and non-interventional treatment methods. In an effort to explore the creation of a stepwise functional restoration through a physiotherapeutic algorithm, a consensus-building symposium was held in Malibu in 1987. As noted above, the core principles of the algorithm generated by this group include patient motivation, desensitization, and reactivation facilitated by pain relief; the use of pharmacologic and/or interventional procedures to treat specific signs and symptoms; and cognitive behavioral psychotherapeutic techniques. As a result of the conference, the symposium members produced a white paper about the purpose and usefulness of an assortment of functional restoration treatment designs; they also recommended formal treatment guidelines (level 3) [1].

The “Malibu” guidelines created some new problems. First, although these guidelines recognize several specific interventions to be applied (physical, pharmacologic, anesthesiologic, and psychologic), they offer no recommendations regarding optimal sequence or duration of these various interventions. Second, the original Malibu guidelines stressed the concept of time contingency, that is, the implication that all “patients should progress through each treatment level in 2 weeks or less” [1], which has proven to be far too rigid and unrealistic in this complex syndrome. Third, the guidelines assert that drugs, sympathetic blocks and psychotherapy should be reserved and used only in cases where progress in the functional restoration-based algorithm has not been achieved. They fail to recognize the frequent necessity of providing medications, blocks, and psychological support *from the beginning* (and not “reserve” these interventions until after a patient has “failed to progress”). In our experience, it is more often than not the case that multiple interventions are required to get a patient started adequately in a functional restoration process.

##### The Minneapolis Conference

Because of these and other issues, a second expert panel (the Minneapolis Group) revisited the Malibu guidelines in August 2001, along with the pertinent literature up to that time. In response to clinical evidence suggesting that sequencing and timing of the treatment guidelines could be improved (e.g., under certain circumstances, concurrent rather than linear utilization of interdisciplinary interventions provided optimum treatment), the Minneapolis group recommended the use of concurrent “pathways,” which were still built upon the original domains of rehabilitation, pain management, and psychological treatment. Additionally, the Minneapolis group liberalized the use of analgesic modalities, deemphasized time-contingency, while preserving the focus on function [58].

Both the Malibu and the Minneapolis groups emphasized the pivotal importance of functional restoration. Both acknowledged that pain management was important, but because pain reports can be heavily influenced by psychosocial state and reinforcement contingencies, pain was considered secondary to function as an outcome due to the latter’s more objective nature. Both groups recognized, however, that pain would logically drive the type, quality, intensity, and pace of other interventions used to achieve the primary, functional outcomes. The next sections provide a detailed examination of each therapy directly involved in functional restoration.

### Interdisciplinary versus Multidisciplinary

Although interdisciplinary treatment programs are clearly the sine qua non of CRPS treatment (holistic, planned team treatment with special training of all modalities; meeting frequently to assess plan, progress/problems and re-plan as a team), this level of intensity is often unavailable except in large urban or academic centers. Payors often consider these interdisciplinary programs to be “too expensive” (although in actuality, our urban 4 week program costs 1/3 to 1/2 as much as a single spinal cord stimulator implant, and this doesn’t consider maintenance, re-implant with lead failure etc.) and opt for less effective, but better understood single modalities. Whatever the rationale for interdisciplinary unavailability, the next best option is a multidisciplinary approach. This approach is traditionally characterized by a single lead practitioner (usually a physician or psychologist) organizing and arranging for specialty training and coordination of local resources, and then referring the patient to these separate modalities. Unfortunately, this is labor intensive (and poorly reimbursed) for the knowledgeable lead practitioner, as the only coordination is via the reports of the isolated modalities (e.g., no real “team”). However, in the context of a multifactorial disease such as CRPS, and the uncertainty as to which modalities are “going to work,” either of these options is far superior to an unidisciplinary/unimodal approach.

### Occupational Therapy

Occupational therapists are the ideal therapeutic leaders in the functional restoration process, as they are trained in the bio-psycho-social principles of disease and are primary in functional assessment and treatment [3, 89]. The Occupational Therapist (OT) role begins by evaluating current functional use of the affected extremity. For instance active range of motion is measured using a goniometer, and edema is gauged with either circumferential measurement or a volumeter [3]. Emphasis is placed on assessment of coordination/dexterity, skin temperature/vasomotor changes, pain/sensation, and use of the extremity during activities of daily living (ADL). While the OT evaluation process has remained consistent over the past few decades, the OT treatment of CRPS specifically has undergone a shift.

Research has expanded the interventional focus to include the early stages of movement (activation of premotor and primary motor cortices) through graded motor imagery or mirror visual feedback (MVF) therapy. Ramachandran first described the use of mirrors to decrease pain or positional discomfort in those suffering from phantom limb pain (level 3) [90]. McCabe et al. expanded the study of MVF treatment to determine its efficacy specifically in persons with CRPS (level 3) [77]. This work illustrated the benefits of this technique in those with early and intermediate CRPS; however, MVF demonstrated no significant effect with chronic CRPS [77].

In an effort to target those with longstanding CRPS, Moseley et al. designed a graded motor imagery (GMI) program to sequentially activate the premotor and primary motor cortices through limb laterality recognition, motor imagery, and lastly mirror therapy [75]. This program appeared to be particularly useful, in that, the premotor cortex may be activated without setting off other cortical networks involved with movement [75]. The mechanisms that underlie any benefits of MVF and GMI are still somewhat unclear. Many researchers believe that this process is partially influenced by forced attention to the affected extremity, decrease in kinesiophobia, increase in large fiber inhibition, and the reconciliation of sensorimotor incongruence [91]. The protocols outlined by McCabe for MVF and Moseley for GMI can be considered loose treatment parameters, but both emphasize the importance of a patient-centered approach that is guided by the clinical observation of presenting symptoms and response to treatment [75, 91].

MVF therapy, as outlined by McCabe [91], first asks the patient to close their eyes and describe both the affected and unaffected limb (i.e., size, location, and any perceived differences), followed by imagined movements of both extremities. The movements for the program are focused on painful joints and those that are just proximal and distal to the joint. The participant is then invited to look at the mirrored limb without movement in order to try to achieve ownership. The recommended frequency and duration of the home program will vary to some degree. However, the overall emphasis is on short sessions (no more than 5 minutes) occurring frequently (5–6 times throughout the day) [91]. Moseley’s GMI program extends over a 6-week period (2 weeks spent in each phase of treatment) and begins with limb laterality recognition using pictures. Secondly, the participant views a picture of an extremity and is asked to imagine moving into that position. The third and final stage involves viewing the reflected image of the unaffected extremity moving through different planes of movement [75]. This process is available on mobile applications like Recognise^TM^ Apps and often used in CRPS treatment programs. Both researchers identify contraindications to these programs, including the inability to establish ownership of the mirrored extremity, increase in pain, and any increase in movement disorders.

While the theoretical underpinnings of these techniques are still under examination, use of both GMI and MVF in CRPS treatment has been increasing [75, 91]. The ultimate value of these approaches remains to be proven in definitive trials. The seminal GMI study was only a small (n = 13) randomized controlled trial comparing efficacy of the intervention described above (n = 7) to a “standard treatment” control group (level 2) [75]. Despite the small sample, results indicated that the GMI intervention resulted in significantly greater improvements in pain intensity than did standard treatment. Although intriguing, somewhat larger follow-up studies found that this graded motor imagery intervention failed to improve pain, and in one study, appeared to *increase* pain and edema (level 2) [92]. Another novel intervention based on a similar rationale as GMI has also shown promise in initial work. In a well-controlled pilot study, virtual reality feedback that paralleled active movements of the extremities (“virtual body swapping”) was examined as a CRPS intervention, and resulted in short-term improvements in body perception disturbance, although did not significantly alter pain (level 3) [93]. As for GMI, the potential therapeutic value of this type of intervention in clinical care remains to be proven in more definitive clinical trials. It is of note, MVI and GMI therapies were developed subsequent to creation of the Malibu and Minneapolis algorithms.

Following the implementation of MVF or GMI, the next treatment objectives for CRPS are to minimize edema, normalize sensation, promote normal positioning/decrease muscle guarding, and increase functional use of the extremity in order to increase independence in all areas—work, leisure, and ADL [48]. In severe cases of CRPS, functional splinting may be appropriate to promote improved circulation/nutrition to the area as well as to facilitate more normal tissue length/positioning during the rehabilitation process, although possible symptom exacerbation due to continuous splinting should be closely monitored [94]. The next steps in treating CRPS are to initiate gentle active movements. manage edema, and institute preliminary desensitization techniques [1]. Edema is managed [level 4] using specialized garments and manual edema mobilization [3]. Superficial or surface desensitization techniques are implemented to assist with normalizing sensation to the affected area [level 4].

The OT then introduces a stress loading program to initiate active movement and compression of the affected joints [45, 46]. Though stress loading may initially produce increased symptoms in the extremity, after several days a decrease in pain and swelling will usually begin to be evident. General use of the affected extremity during daily tasks is strongly encouraged throughout the rehabilitation process [45]. Stress loading consists of two principles: scrubbing and carrying [45]. Scrubbing consists of moving the affected extremity in a back/forth motion while weight bearing through the extremity [45, 46]. The scrubbing can be accomplished using a scrub brush and is usually done with the patient in a quadruped (for upper extremity involvement) or elevated sitting (for lower extremity involvement) position. Positions can be modified to facilitate maximal performance and compliance. For example, upper extremity scrubbing can be done in a standing position or a handled scrub brush can be used for persons with CRPS [94–96]. The amount of weight placed through the affected extremity and the duration of the activity are gradually increased. The Dystrophile^®^ is a patented device designed to assist with maintaining consistent weight bearing and compliance with scrubbing by activating a light when the patient has reached the preset load. However, this device does not hold any proven advantage over a simple scrub brush.

Carrying is the second component in stress loading. In the upper extremity, weight loading continues with small objects carried in the hand, soon progressing to a handled bag, which can be loaded with increasingly heavy weights. The weight should be carried throughout the day whenever the patient is standing or walking [45, 46]. The lower extremity can be loaded in a variety of ways. Walking is an important loading technique if care is taken to ensure weight bearing through the affected leg during gait, especially when an assistive device is used. Increased weight bearing can be accomplished with verbal/physical cueing or by having the patient carry a weighted object on the affected side. Loading can also be facilitated by engaging the patient in activities that promote weight shifting/balance (e.g., ball toss) or by placing the non-affected foot onto a small footstool during static standing tasks [3]. While stress loading appears to demonstrate utility in the clinical setting (level 4), further study is needed to demonstrate its efficacy relative to other functional weight bearing interventions.

Once the patient is actively engaged in an edema management and stress loading program, treatment can progress toward increasing functional use of the extremity. As the pain and edema decrease, the patient will be better able to tolerate and participate in active range of motion, coordination/dexterity, and strengthening tasks [3]. Proprioceptive Neuromuscular Facilitation (PNF) patterns are often well tolerated during the rehabilitation process. PNF promotes “response of the neuromuscular mechanism through stimulation of the proprioceptors” [97]. PNF patterns are spiral and diagonal combinations of motion that “permit maximum elongation of related muscle groups so that the stretch reflex can be elicited throughout the “pattern”” [97]. These patterns, similar to normal movement patterns, facilitate strength and balance while increasing ability to perform ADL.

The overall role of the OT during CRPS rehabilitation is to guide the patient through a program designed to minimize pain and edema while maximizing functional use of the extremity [3]. As CRPS varies greatly in severity and duration, it is very important for the therapist to competently upgrade/downgrade programs according to therapeutic response as well as maintain enthusiasm in support and encouragement of the patient during the rehabilitation process.

The vocational counselor and OT should work closely together (see below) when assessing return to work goals, especially when potential to return to a specific job is being assessed. Services including job site analysis and job-specific reconditioning or work hardening, work capacity evaluation, transferable skills analysis, and a formal functional capacities evaluation should be considered [98]. Allowing the patient an opportunity to participate in a trial work period before providing final release for work is often an excellent way to observe his/her ability to return to work and perform job duties as well as further assess work behaviors. Return to work can be therapeutic from a psychological perspective, assuming the work activities will not aggravate the problem and increase long-term pain [99]. Provision of release for work should be coordinated by the vocational counselor. Information included should be gathered from all disciplines including occupational therapy, physical therapy, and the physician. It should include detailed instructions when releasing patients to limited duty, or prescribing a job change. Functions to be considered and potentially modified include: lifting, pushing, pulling, crouching, walking, using stairs, bending at the waist, climbing, awkward and/or sustained postures, tolerance for sitting or standing, hot and cold environments, data entry and other repetitive motion tasks, sustained grip, tool usage, and vibration factors. Releases for sedentary or light duty should always list specific physical limitations. In situations where a job is not available, vocational counseling, evaluation, and job placement services should be considered to assist patients with addressing return to work goals as soon as possible (all level 4; see below)

### Physical Therapy

Physical therapy (PT) clearly plays a critical role in functional restoration, and PT activities are designed to complement those of occupational, recreational, and vocational therapy. According to the experienced Mayo Clinic pain management group, “Physical therapy is the cornerstone and first line treatment for CRPS” [100]. The physical therapist can help patients increase their range of motion, flexibility, and later strength, through the use of gentle progressive exercise. The physical therapist tries to improve all functional tasks, such as gait training (in lower extremity CRPS), and coordinates/collaborates on all OT, recreational, and vocational goals.

An ongoing discussion concerns the distinction between pain-contingent physical therapy and time-contingent physical therapy approaches. lt is generally accepted that PT should be executed within the bounds of the patients’ tolerance [101] and never when the affected limb is insensate (such as immediately after a block) or with CRPS Type II patients who present with pronounced hypoesthesia. Inappropriately aggressive PT can trigger extreme pain, edema, distress, and fatigue, and may in turn exacerbate the inflammatory and sympathetic symptoms of CRPS; it is therefore to be avoided. Use of assistive or range of motion devices, prolonged application of ice, and inactivity may also aggravate CRPS. Physical therapists must teach patients with CRPS that they will experience pain both when they exercise too much *and* when they exercise too little. Patients must therefore be taught to seek the “happy medium,” and it is the physical therapist's responsibility to help them find that therapeutic ground and help them to steadily advance toward a more functional and active lifestyle. In a series of RCTs, Oerleman’s group has shown that PT (and to a lesser extent OT) improves pain ratings and “active mobility” compared to patients receiving only counseling (from a social worker) in upper extremity CRPS cohorts (level 2) [64, 70]. The principal objective of the physiotherapeutic treatment protocol as investigated by Oerlemans et al. is to enable the patient to gain the greatest possible degree of control over his or her symptoms while relentlessly pursuing goal of reanimating the affected part. A specific set of questions, standardized assessment scales, and objective tests carried out during observed tasks and physical examinations are used to gain an impression of the degree of segmental dysregulation and the extent to which pain can be managed. The treatment program is set up on the basis of the information obtained. It comprises a number of physiotherapy interventions such as support, exercise therapy, improving skills, and relaxation therapy. The key components of this protocol are increasing the degree of control over the pain, improving the way the patient copes with the syndrome, treating any dysregulation, and improving skills; for example, by training and practice in compensatory skills, and posture and movement instruction.

Efforts to improve mobility can start as soon as pain levels have become more tolerable to the patient. The emphasis is on self-determined, active, and functional movement. Attention needs to be paid throughout the entire course of treatment to maintaining as normal a posture and movement pattern as possible and to preventing negative compensatory changes to adjacent joints and muscles (for example, changes brought about by contraction). This is supported by The European Pain Federation task force who recently created standards for the diagnosis and management of CRPS. They submit that early appropriate graded exercises may preserve limb function and shorten the disease course [102]. As described above, GMI may be integrated as one component of a comprehensive PT intervention. EFIC has created a free web site to assist the design of the physical therapy program: https://crps.europeanpainfederation.eu/#/.

The value of PT in pediatric CRPS also has substantial empirical support. In children with CRPS, a randomized controlled trial of PT (combined with cognitive-behavioral therapy) provided once per week vs. three times per week revealed significant improvements in both groups on five measures of “pain and function,” with sustained benefit in “the majority” of subjects (level 2) [72]. In a prospective case series following 103 children with CRPS, “intensive PT” (aerobic, hydrotherapy and desensitization) supplemented by “psychological counseling” (in 77%) was “effective in initially treating childhood CRPS and is associated with a low rate of long-term symptoms or dysfunction” (level 3) [103].

The physical therapist should instruct the patient in the avoidance of physical stressors as much as possible (i.e., the stress of extended inactivity and bed rest on one extreme, and the stress of excessive exercise on the other). Alongside the goal of a gradual increase of strength and flexibility the therapist should encourage pacing and include rest breaks and relaxation techniques as well. PT goals can also be achieved with the use of devices, including foam rubber balls succeeded by spring-grip strengtheners for the upper extremity, and Swiss balls, foam rolls, and anti-gravity resistive equipment (such as a Pilates reformer) for the lower extremity. These devices help to gradually introduce a variety of weight-bearing/strengthening techniques.

Preliminary data suggest that graded exposure therapy to exercises the patient may perceive as “harmful” can lead to a reduction of disability as a consequence of the reduction of pain related fear of movement (level 3) [104]. The program developed by Vlaeyen and colleagues, consists of an educational program explaining the “fear-avoidance model” (pain leading to catastrophic thoughts, leading to avoidance and more pain and disability), combined with a tailored exercise program aimed at activities most feared by the patient.

Taking a “gradual” loading approach perhaps a step too far is the so-called “Pain Exposure Therapy” as described by van de Meent et al. [105]. This program consists of progressive-loading exercises tailored to specific body functions using regular physical therapy techniques such as passive and active exercises to mobilize joints and muscle stretching. The physical therapist thereby mainly acts as instructor, rewarding functional progression and providing schedules for exercises and activities at home. Contrary to most PT interventions, this approach is time contingent, and pain severity is not used as a guideline to increase or reduce therapeutic activities. Preliminary support for this intervention includes pilot data with regard to pain reduction and decrease of functional limitations in a case series of 20 patients (level 3) [105]. This is controversial. Clinical trial outcomes for pain exposure therapy are detailed further in the psychological interventions section below.

Mat exercises provide strengthening of both the affected extremity and the associated postural muscles in a non-weight-bearing approach. Particularly valuable mat exercises include movement therapies such as the Feldenkrais technique (level 4). Feldenkrais teaches and encourages gentle, active motions within the patient’s available range to increase body awareness and promote appropriate movement patterns. A fundamental aspect of mastering proper movement patterns is the relearning of proprioception. The physical therapist can help patients achieve mastery by teaching them neuromuscular proprioception exercises, advancing them as they gain proficiency.

Related to re-establishing body awareness in CRPS patients, behavioral programs including graded sensorimotor retuning exercises may provide decrease of pain and improvement of tactile discrimination sense, perhaps coinciding with the restoration of symmetrical cortical limb representation in the SI and SII regions of the brain [80]. This pain contingent intervention, aimed at reestablishing proprioceptive abilities and desensitization, has shown preliminary efficacy in a cohort of six CRPS patients (level 3) [80]. Likewise, in a small pilot study comparing 7 CRPS patients receiving low amplitude 80–100 Hz vibratory stimulation of the affected extremity combined with regular physical therapy compared to a control group receiving only standard physical therapy (n = 4), Gay et al. found more pronounced improvement in pain severity and range of motion in the experimental group (level 3) [81]. According to the authors, the mechanism of action could be related to activation of cortical areas involved with motor command and movement representation.

Clinical experience indicates that that many (if not most) patients with advanced CRPS will present with myofascial pain syndrome of the supporting joint. Assertive treatment of myofascial pain is a critical component of successful treatment (level 4) and is principally the purview of the physical therapist. Some schools of thought propose that the myofascial pain syndrome must be treated first, and if successfully treated, the CRPS will perhaps resolve [106]. However, this provocative assertion remains unproven.

Aquatic therapy can be especially valuable to CRPS patients because of hydrostatic principles and “the buoyancy effect” [107]. Hydrostatic pressure provides a mild compressive force around the extremity that may help decrease the edema that is widespread in CRPS. Aquatic therapy also provides an outstanding opportunity for introducing lower extremity weight bearing, and the buoyancy it provides may be especially useful for early restoration of functional activities such as walking. When conducting aquatic therapy, care must be taken to maintain water temperature, because excessively cold or hot water may temporarily exacerbate the CRPS. Water therapy may allow early participation in progressive PT, as nearly all exercises that are executed on land can be executed in the water, where the water adds resistance without adding full stress/weight to the joints. This of course is groundwork to full weight bearing, particularly in the lower extremity.

Hands-on techniques such as gentle massage and myofascial release can sometimes offer effective relief from the myofascial pain. Massage is often mentioned, but although it has not been studied in a controlled manner (level 4 evidence only), clinical experience indicates it may help decrease edema in certain cases but must be gentle and careful. Although peer-reviewed evidence is lacking, electrostimulation modalities have also demonstrated some efficacy in our clinical experience, but ultrasound therapy has appeared less effective. Contrast baths are another possible, if controversial, treatment option for CRPS patients. Based on the clinically accepted principles of alternating heat and cold, mild contrast baths can in principle be beneficial in early CRPS cases to facilitate improved circulation in the affected extremity by alternating vasodilation with vasoconstriction. However, the vasomotor changes in advanced cases of CRPS do not allow for the desired response, and the immersion of the limb in cold water may exacerbate CRPS symptoms. Contrast baths for advanced cases of CRPS are therefore not recommended, and it is noted that there is little empirical support for this approach in CRPS of any duration. While clinical experience indicates that PT can benefit effective CRPS management, most evidence regarding efficacy of specific PT techniques comes from studies representing level 3–4 evidence. This low level of evidence has been verified by CRPS-specific meta-analyses [108, 109]. More high-quality studies are needed to determine the best evidence-based PT management approach for CRPS patients.

### Recreational Therapy

Although lacking published outcome studies to support its use, we believe for completeness that it is important to address recreational therapy as a potential component of CRPS care. The following is based solely on our clinical experience with multidisciplinary CRPS treatment that includes a recreational therapy component.

Because recreational therapy employs enjoyable activities, the recreational therapist is frequently the first clinician to succeed in getting the CRPS patient to initiate increased movement in the affected part, a primary goal of successful treatment. The incentive of returning to a favorite pastime is often an appropriate tool to break through the “kinesiophobia” and bracing that often attend CRPS [110]. Through the use of modifications, adaptive equipment, and creative problem solving (e.g., using large handled gardening equipment for gardeners, bowling with the non-dominant hand for bowling fans, and substituting biking in place of running for athletes, etc.), a patient can find fulfillment in previously lost or new recreational activities. Recreational therapy re-establishes the patients’ ability and freedom to determine their own leisure lifestyle choices. The increased social contact engendered by these activities will, in turn, heighten the patients’ chances of remaining active within the community after treatment.

With a bit of advanced planning, recreational therapy can complement PT and OT treatment goals. For instance, a recreational therapist could reinforce an OT scrubbing protocol by instructing a patient to use an affected upper extremity to sand wood in a recreational project. Such planned convergence of goals affords the patient the twofold satisfaction of creating something and simultaneously accomplishing therapy goals [111]. For example, a patient who is engaged in a desensitization program and who also enjoys gardening can be assigned “horticulture therapy” (i.e., the use of the hands to work soil).

Additionally, recreational therapy can promote flexibility and range of motion. The recreational therapist should plan activities that patients find inherently enjoyable, because patients are more willing to take on fine-motor grasping and releasing tasks for longer periods of time if they are engaged (e.g., beading a necklace, holding a watering can, playing a card game, practicing on a keyboard etc.). A recreational therapist must be creative, because a happily engaged patient will be more inclined to fulfill therapy goals when engaged in fun activities like putting golf balls, playing balloon volleyball, or shooting pool.

In addition to advocating new leisure skills, recreational therapy concentrates on reintroducing the patient to stable community involvement. During structured community outings, the CRPS patient can focus on carrying and loading a bag (i.e., “loading”) with the affected limb [45, 46]. This task can be accomplished with a water bottle, shopping bag, or purse. Other tasks can involve weight bearing and follow through with gait training on unlevel surfaces within a realistic community setting. Identified and achieved appropriately, successfully completed tasks can increase patient self-confidence and promote the incorporation of these learned skills both at home and within other therapy sessions.

In summary, recreational therapy may help combat kinesiophobia and promotes increased movement, although this view is not supported by systematic research. Recreational therapists work closely with other disciplines to achieve the therapeutic goals of CRPS patients, and they implement creative tactics that achieve those goals while giving patients more decision-making freedom and more fun. Most significantly, recreational therapy can reintroduce balanced leisure activities into the lives of the patients whose conditions may have discouraged such behavior, with resulting psychological and quality of life benefits. Unfortunately, Therapeutic Recreation is often not or not well reimbursed, and OT or PT practitioners will need to be familiar with this role.

### Vocational Rehabilitation

The following section addresses the potential role of the Vocational Rehabilitation (VR) counselor in optimizing CRPS treatment outcomes, and as was the case for recreational therapy, is based entirely on our clinical experience with a multidisciplinary CRPS treatment program including VR as an intervention component. To our knowledge, formal studies of VR as a specific CRPS intervention are absent from the literature. The VR counselor helps prepare the CRPS patient for a possible return to work, or the “ultimate” functional restoration. VR involves restoring a patient (if possible) to their original vocational activities as expediently and as safely as possible. Counselors use information from medical, occupational, educational, financial, and labor market fields to make return-to-work assessments. Vocational counseling addresses benefits of work and accommodations, as well as job modifications and the utilization of pain management techniques. The VR specialist can also help each patient to identify with the role of worker and assist in creating a plan for a return to work.

If possible, the VR counselor should understand all of the physical demands of the job before addressing return-to-work issues. Review of job description and consultation with employer, supervisor, employee health nurse, or other human resource specialist, and work site visit (when appropriate) are steps recommended to address specific job duties, especially when determining ability to provide a full duty release [99], or when recommending specific restrictions and modifications. The VR specialist also provides job and job site analyses, and uses that information to coordinate job-specific reconditioning or work hardening, work capacity evaluation, transferable skills analysis, and a functional capacities evaluation [98]. The VR counselor must determine whether or not a patient can return to the original job. The counselor must also consider the alternatives of returning the patient to either a modified version of the previous job or an alternate job with the same employer, or whether a new job placement referral will be needed when return-to-work with the previous employer is not an option. The VR counselor and occupational therapist should work closely together when assessing return-to-work goals, especially when assessing the possibility of returning to a specific job.

The VR specialist must possess a thorough understanding of the prior job description, requirements, and, occasionally, the required vocational testing and targeted retraining of the CRPS patient who intends to return to work. Initially working with the OT, the VR specialist assesses a patient’s work activities and provides a simulation of them for the patient in a controlled clinical environment. In the final steps of the VR process, the specialist can provide work capacities, along with functional capacities and targeted work hardening in order for the patient to return to gainful employment. Competent VR requires a proficient specialist capable of maintaining a methodical, informed, and experienced approach in order to grasp and successfully navigate the Byzantine social and medico-legal quagmires in which CRPS patients may find themselves. As with all the interdisciplinary specialists, the VR specialist must sustain ongoing communication with the others on the team and keep the team informed of each patient’s individual vocational situation and progress.

VR specialists regularly encounter hurdles to appropriate return-to-work functions. Firstly, health factors are often presumed to have the greatest impact on worker disability, but social scientists have argued that the most important determinants of work status for persons with chronic disease are actually age, education, job satisfaction, and job status in the labor force [112]. Secondly, other factors such as work history, employment in public sector versus private, current work status, lower socio-economic class, level of education, and lack of varied work may also predict work disability for patients with chronic pain [112]. Thirdly, long periods of unemployment or reduced employment activity may impact vocational potential. Chronic pain sufferers may have been out of work for long periods of time before they are referred to a VR specialist, and employers are often reluctant to employ persons who have chronic pain, have been unemployed for long periods of time, or who have workers’ compensation cases [113]. Additionally, the ability to modify the work environment in accordance with limitations has major implications in limiting the extent of the disability and/or preventing reinjury or new injuries [112].

Although VR is frequently the final step of rehabilitation therapy, addressing return-to-work issues early is critical so as to set employment as a long-term goal [114]. Allowing the patient an opportunity to participate in a trial graduated time/effort work period before providing final release for work is often an excellent way to observe his/her ability to return to work and perform job duties, and it also provides an opportunity to further assess work behaviors and capacity. In addition, the initial graded increase of time and effort spent at work greatly alleviates significant patient anxiety and thus improves chances of successful return-to-work. Return-to-work can be a form of therapy, provided the work activities do not exacerbate the problem or increase long-term pain.

The VR counselor should coordinate the provision of release for work by assembling information from all disciplines. Releases for sedentary or light duty should always list specific physical limitations, and the releases for limited duty should include comprehensive instructions. When preparing a release for work form, the VR specialist must take into account the abilities of the patient, including: lifting, pushing, pulling, walking, crouching, using stairs, using tools, bending at the waist, maintaining awkward and/or sustained postures, maintaining a sustained grip, tolerating extended sitting or standing, tolerating extensive data-entry functions and other repetitive motion tasks, tolerating hot and cold environments, and tolerating any severe vibrational factors. Any number of these factors may require modification of the work environment, particularly in chronic or severe CRPS. As is the case for RT, the value of VR in CRPS management is based solely on clinical experience rather than systematic research.

### Other Therapeutic Interventions of Note

Hyperbaric oxygen therapy was assessed in a medium sized randomized control trial (RCT) and produced a significant decrease in pain and edema versus “normal air” (level 2) [115]. While intriguing, these findings of increased oxygenation having clinical benefits seem somewhat contrary to other work suggesting that CRPS may be adversely influenced by elevated oxidative stress [116–118]. These findings require replication, and cost-benefit considerations of this therapy will also be important to consider, given the expense of the equipment required. Acupuncture is mentioned in many treatment reviews. There are two RCTs that evaluate acupuncture treatment in CRPS patients. One was very small (n = 14) and failed to show a significant difference in outcomes [119]. The other had a larger sample size (n = 96) and reported improvement in pain severity and motor function of affected limbs 40 days after treatment in patients with CRPS type I following stroke (level 2) [120]. However, considerably more research is needed to fully demonstrate the utility of acupuncture in CRPS. Finally, there are also case studies that suggest that chiropractic manipulation may reduce pain and enhance range of motion and function in CRPS patients (level 4) [121].

In summary, because the symptoms of CRPS patients encompass all of the bio-psycho-social complexities of chronic pain, the best hope of helping our patients is the adoption of a systematic, stable, empathetic and, above all, interdisciplinary approach that addresses those symptoms (or if impractical due to availability, a multidisciplinary approach). That functional restoration can and should be the central intervention and outcome standard in CRPS is a theory that must be tested. Until then, the interdisciplinary approach for treating patients with CRPS remains the most pragmatic, helpful, and cost-effective therapeutic approach available today.

## Pharmacotherapy

For the past 150 years, multiple drug treatments for CRPS have been tried. One of the first drugs mentioned was laudanum (tincture of opium) by Weir-Mitchell (who coined the term causalgia) and his use of the” new invention,” the hypodermic syringe, to perform cocaine nerve blocks [122–125]. Unfortunately, most medications used clinically to manage CRPS have not yet been tested adequately in high quality, double-blinded, randomized, controlled trials (RCTs). This absence of multiple trials to document efficacy of many pharmacotherapy agents is attributable to many factors, including previous lack of uniformly accepted diagnostic criteria (preventing generalization across studies), the low prevalence of this rare disease causing difficulties in recruitment, as well as lack of funding for trials using promising older agents without patent protection to provide financial incentives [27, 126]. Fortunately, with the Food and Drug Administration’s designation of CRPS as a Rare Disease in the past decade, industry interest in conducting definitive trials for CRPS therapeutics has increased (www.orpha.net; www.clinicaltrials.gov).

The resourceful clinician will extrapolate from RCTs, meta-analyses, and systematic reviews concerning treatments for related neuropathic conditions [127, 128] and ultimately utilize empirical drug trials in each patient, based on consideration of what mechanisms seem most germane. However, repeated trials of ultimately ineffective drugs can also lead to patient frustration and disengagement [124]. CRPS differs from other neuropathic pain syndromes by having additional tissues and systems involved, including the microcirculation, bone, and inflammatory pathways [129]. In fact, although qualities of CRPS pain are often neuropathic, by currently accepted standards the condition does not fulfill criteria for neuropathic pain [130]. Reliable data now show variable involvement of central sensitization [131], motor abnormalities [132], and sympathetic efferent features [131] at different times and in different individuals suffering from CRPS [26, 129]. It is very likely that there will never be a single medication that will effectively treat all patients with this multi-factorial disease [26, 129].

Medications trialed specifically for CRPS include calcitonin and bisphosphonates, and several immune modulating drugs. Treatments better studied in neuropathic pain include tricyclic anti-depressants, gabapentin and pregabalin, carbamazepine, opioids, clonidine, nifedipine, α-adrenergic antagonists, lidocaine patches, and topical capsaicin. This section summarizes the outcomes from the few CRPS trials, as well as the pertinent trials for related neuropathic pains. As with most treatments, drug therapy works best when prescribed in conjunction with functional restoration and treatment of other comorbid conditions (please see above).

### General Considerations

- To help avoid unrealistic expectations, patients should be told that while there is no treatment proven to cure CRPS or reduce symptoms in all patients, the drugs that patients will receive during their treatment have been shown to help with CRPS for some patients.
- Recognize that CRPS may be more than just a single unitary condition. Early CRPS (up to 6-18 months duration) can respond differently to interventions than persistent CRPS [133]. Importantly, patients diagnosed with early CRPS likely will naturally improve [27, 30] and drugs or nerve-blocks that are effective even for only a few months may bridge the time to natural recovery. Approximately 15% of early CRPS patients fail to recover [27], and an early cold limb may be a poor prognostic sign [134].
- Understand that most patients will over time develop analgesic tolerance to available drugs whereas side effects often continue.
- Available drugs are not thought to “cure” the condition.
- Monotherapy is best, to minimize adverse effects, cost, and patient non-compliance, but rational polypharmacy is often needed, particularly to address the various CRPS symptoms (and disease subtypes). This of course should comprise rational combinations of different classes of medications rather than multiple medications from the same class.
- The choice of medications should include cost considerations and other patient needs.
- Drugs that simultaneously treat multiple symptoms are desirable, for example, tricyclic antidepressants are relatively effective in RCTs for relieving neuralgic pain and also effective for anxiety, depression, and insomnia [27, 128].
- Traditionally, as needed (PRN) drug intake was considered inferior to scheduled drug intake. However, recognition that patients often develop tolerance to regular analgesic drug intake has changed this consideration somewhat. Some patients may benefit from taking certain drugs as and when required, for example after a bad day, or in anticipation of a difficult night. More research is needed to clarify this question.
- Reasonable treatment outcomes should be agreed upon in partnership with the patient before treatment starts (e.g., a pain reduction of two points on a 0–10 scale, improvement in specific functional activities). If these targets are not achieved, or if initial beneficial effects later lessen, the drug treatment should then be reconsidered.

### Anti-Inflammatory Drugs

Given their anti-inflammatory mechanism of action, nonsteroidal anti-inflammatory drugs (NSAIDs), COX-2 inhibitors, corticosteroids, and free-radical scavengers are potentially useful for addressing pain that may be related to the inflammatory component of CRPS. However, CRPS inflammation may be largely neurogenic (initiated by inflammatory mediators from the terminals of afferent nociceptors), and no drugs have proven effective for this type of inflammation [51]. Many patients may have spontaneous improvement in inflammatory features over time [31]—even those patients whose pain does not get better.

This class of drugs would appear to be potentially useful for both prophylaxis and rescue, although this has not been directly evaluated in clinical trials. NSAIDs inhibit cyclooxygenase and prevent the synthesis of prostaglandins, which mediate inflammation and hyperalgesia and thus may inhibit nociceptive processing [135, 136]. Our clinical experience finds NSAIDs effective for some CRPS patients (level 4 evidence). In addition to treating CRPS, NSAIDs have also been used to treat other neuropathic pain conditions, particularly when associated with inflammation (level 3 evidence) [51, 135–139]. CRPS usually affects distal extremities, while more proximal muscle groups, such as in the shoulder, frequently hurt (in response to persistent guarding of the limb) without directly being affected by CRPS. This type of myofascial pain/muscle pain may respond to NSAIDs.

Research support for NSAID utility in CRPS is lacking, with one study showing no analgesic value in treating CRPS [140]. Specific NSAIDs may be more useful than others. Ketoprofen, for example, may have substantial anti-bradykinin and anti-prostacyclin effects in addition to the typical anti-prostaglandin effect [141]. Inhibitors selective for cyclooxygenase-2 (e.g., celecoxib) have not been tested in CRPS, although are reported anecdotally to be of some use (level 4 evidence) [142–144]. Highly publicized concerns of cardiac risk somewhat limit the widespread use of COX-2 inhibitors [144]. We note that acetaminophen is currently not recommended for treatment of chronic disease due to the potential for liver toxicity with regular or high dose use (www.fda.gov/acetaminophen).

### Immune Modulation

Some evidence (level 2) suggests that very early initiation (after trauma) of steroid treatment (approximately 30 mg/day for 2–12 weeks, followed by a taper) may be effective [145, 146]. A systematic review [145] evaluated one of these trials and found it to be low quality. Given the data, a short course of steroids may be indicated in early CRPS with prominent inflammation, but longer courses are unproven [138] and there are numerous, serious contraindications to chronic steroid use [147] In persistent CRPS, intermediate-dose steroids (1 g orally in total, taken over 2 weeks) are rarely effective and this treatment often causes side effects [148]. High-dose pulsed treatment (3x1g iv) as used in autoimmune conditions has not been evaluated. A single intrathecal steroid injection was shown ineffective [149].

A randomized controlled trial (level 2) of low-dose (0.5 g/kg) intravenous immunoglobulin (IVIG) treatment for persistent CRPS indicated efficacy [150]. However, a subsequent larger trial was negative [151] Although there is some anecdotal evidence for the efficacy of high-dose IVIG this has not been formally tested. The TNF-alpha blocker, infliximab, was ineffective in a preliminary RCT (level 3) which was stopped early [152]. Treatment with lenalidomide which has strong anti-TNF activity was also not effective, having been assessed in one of the largest RCTs (level 2) in persistent CRPS conducted to date [153, 154]. A small, open-label randomized trial (level 3) of mycophenolate (1.5 g BID) suggested efficacy [155] but a larger trial would be needed to confirm these findings. Plasma exchange has been reported effective (level 4) in several case series [156] and consequently CRPS has been added to the list of possible indications by the international plasma exchange society (ASFA) [157]. In our clinical experience (level 4), plasma exchange treatment, although providing pain improvement in some cases, requires use of long repeat-exchange cycles (e.g., eight exchange treatments over 4 weeks) and is unfortunately complicated regularly by pain increase at the venous access site. A trial of low-dose naltrexone, an opioid-antagonist drug that has potential immune modulatory properties is currently recruiting (clinicaltrials.gov). Finally, in a small, preliminary trial (level 3), 4/7 included patients with CRPS appeared to have pain reduction after treatment with the epidermal growth factor inhibitor Cetuximab (2/7 had pain relief after placebo) [158].

Since autoantibodies have increasingly been implicated in CRPS pathophysiology [159–162], it is possible that a combination of several of these drugs may be useful, but definitive trials are currently lacking. Emerging immune treatment strategies include a reduction of the autoantibody-serum titer and modification of antibody downstream effects. A number of other therapeutic agents developed for the treatment of other antibody-mediated conditions are available for testing in CRPS, (B-cell or plasma-cell targeting drugs, FcRn receptor blockers, level 4) but trials are currently lacking.

### Bisphosphonates

Bisphosphonates have immune-modulatory properties and modulate bone metabolism [163]. One RCT (in patients with very early “CRPS,” [who had an average disease duration of 5 weeks from trauma , ]has shown some efficacy with bisphosphonate treatment level 2) [164], however, these results have not to date been reproduced elsewhere. Although several small bisphosphonate trials reported efficacy in persistent CRPS [165] two recent large pharma-sponsored trials were either not reported or were reported as negative [163] (level 2; see clintrials.gov). Proponents highlight that it is possible that bisphosphonates will improve selected sub-types of CRPS, but given the occurrence of rare serious side effects RCTs are required to confirm such beneficial effects (level 4). It has been hypothesized that bisphosphonates may work best for CRPS characterized by osteopenia (“Sudeck’s Atrophy”); this subset of CRPS patients may perhaps be identified by bone density or triple phase bone scanning abnormalities (level 4). To date, this hypothesis remains unproven.

### Cation-Channel Blockers

Drugs that block entry of sodium or calcium into neurons reduce their action potentials. Most often used as anticonvulsants, several have efficacy in neuropathic pain documented in large RCTs, meta-analysis and systematic reviews (level 1 evidence) [165–169]. Gabapentin, first-line treatment for neuropathic pain, came to the attention of pain specialists in an anecdotal report of efficacy for CRPS [170]. It works at the alpha(2)-delta auxiliary subunit of voltage-dependent calcium channels and well-powered large RCTs have demonstrated its efficacy^,^ in postherpetic neuralgia (PHN) and diabetic peripheral neuropathy (level 2 evidence) [171, 172]. A case series in adults [170] and one pediatric case report [173] suggest efficacy in CRPS (level 4 evidence). The only RCT of gabapentin in CRPS (level 2) was “negative,” however it used a sub-maximal dose (1800 mg/day) [174]. As gabapentin neared the end of its patent-protection a large pharmaceutical firm developed a closely-related compound, pregabalin, with essentially the same mode of action. Its major advantage is that some patients can manage with twice-daily dosing, but relative cost should be considered when deciding between the two [level 2] There are no clinical trial data evaluating pregabalin for CRPS.

Carbamazepine has a traditional place in the treatment of neuropathic pain, and is FDA-approved for trigeminal neuralgia [175, 176]. One preliminary RCT with an experimental design that included several patients with CRPS responsive to spinal cord stimulator treatment (and the SCS off) indicated that 600 mg/day of carbamazepine, taken over 8 days, had some analgesic efficacy [177]. Oxcarbazepine is a similar anticonvulsant that often replaces carbamazepine because it has fewer serious adverse effects (specifically bone-marrow suppression or liver failure); headaches, dizziness, and nausea are the most common adverse effects of oxcarbazepine [178]. Oxcarbazepine has not been studied specifically in CRPS. Phenytoin is an older agent (with many side effects) sometimes considered in neuropathic pain especially in cases that involve “ectopic nerve firing” (level 2), but there are no reported outcomes in CRPS [179–181]. RCTs for lamotrigine have studied its effects on other neuropathic conditions, but not CRPS [155]. There is anecdotal evidence (level 4) for efficacy of a variety of other anti-convulsants/neuro-modulators (levetiracetam, topiramate) in CRPS, but no compelling clinical trial evidence supporting their use at this time.

### Augmentation of Monoamines

Tricyclic and heterocyclic drugs that augment descending inhibition by blocking presynaptic re-uptake of monoaminergic neurotransmitters (particularly norepinephrine) are unsurpassed in efficacy for neuralgia [128, 182]. Although originally approved for depression (and anxiety), this indication has been supplanted in our patients by use for neuropathic pain [182]. The antidepressant (and anti-insomnia) efficacy of these compounds provides additional benefit for many patients. These are “dirty drugs” with multiple mechanisms including peripheral sodium-channel blockade, which may in fact contribute to efficacy [183]. Their once-daily dosing (preferably with the sedative versions at night) and low cost are added advantages.

A first-line option for neuropathic conditions and headache (level 2 evidence) [184–187] tricyclic/heterocyclic antidepressants (HCAs) are used exclusively as prophylactic agents. Meta-analyses of RCTs support their efficacy for neuropathic pain (level 1) [165, 166, 186]. One study reported that, for every 100 patients with neuropathic pain taking antidepressants, 30 would obtain at least 50% pain relief (i.e., Number Needed to Treat [NNT] of 3 [182, 186]. This is unsurpassed by any other treatment for neuropathic pain; however it is worth noting that all of these previous trials have durations of only weeks to a few months, and in clinical practice many patients may eventually develop tolerance or tachyphylaxis to these drugs [128]. The development of tolerance is less of a challenge in early CRPS where most patients may eventually improve in any case [30]. In established and persistent CRPS, to delay tolerance development some clinicians suggest it is worth considering prescribing these drugs PRN, i.e., aiming primarily to achieve sleep-induction on difficult days.

It is thus useful to be familiar with several tricyclic/heterocyclic drugs, as each possess specific side effects which may be used to patient advantage [187, 188]. For example, an anxious, depressed, thin, insomniac patient may benefit from an anxiolytic, sedative, anti-depressant drug (e.g., doxepin); conversely, an overweight, hyper-somnolent patient with psychomotor retardation may benefit from an antidepressant with more noradrenergic selectivity (e.g., desipramine, which can be activating and can cause weight loss) [185]. Selective serotonin reuptake inhibitors (SSRIs) have not shown any analgesic efficacy (level 4 evidence) [189, 190] but of course are very effective and safe anxiolytic/antidepressant drugs [189, 190]. The NNT for SSRIs in neuropathic pain is much higher than traditional HCAs, for example, 7.7 for citalopram, 2.9 for paroxetine [165].

The tricyclic/heterocyclic drugs are by far the best single agents for managing CRPS. However, these drugs are complicated and have known, expected side effects (some of which can be very useful, such as sedation in insomniac patients, which is nearly ubiquitous; see above). These drugs must be carefully monitored (frequent visits when starting) and started in low dose with methodical, gradual dose increases. The range of ultimate doses is broad, and some patients respond well to low dose; yet once started it is not appropriate to conclude lack of efficacy after a low dose trial. With tricyclics we recommend an EKG before starting at mid-dose range and at ultimate dose due to (rare) interval changes (level 4).

The older (venlafaxine) and newer combined serotonin-norepinephrine reuptake inhibitors (SNRIs) (e.g., milnacipran, duloxetine) are FDA approved for several chronic-pain indications, in addition to major depression. They have not been trialed for CRPS. Venlafaxine, an older SNRI has some anecdotal value for neuropathic pain and perhaps CRPS (level 4 evidence). There is a pressing need for data on comparative efficacy and safety of SNRIs and TCAs in CRPS.

### Opioids

The earliest known expert opinion regarding opioids in CRPS is that of S. Weir Mitchell, who commented that “for the easing of neurotraumatic pain [referring to “Causalgia” most like CRPS type I] the morphia salts … are invaluable.” [123]. His description of the relief which the young soldiers he treated obtained is well worth reading, as it also highlights the issues underpinning the opioid crisis: opioids can work extremely well when taken for short periods; yet many problems arise with longer-term treatment (and patients may find it hard to understand why these drugs should not be available to them long-term). However, outside the battlefield, opioids may in fact be less effective even for short term treatment of CRPS. Only one RCT (level 2) has been conducted in CRPS [177] evaluating controlled-release morphine, and reporting no difference in pain reduction when compared to placebo after 8 days’ use. This trial would not meet today’s quality standards, so the question about short-term efficacy of opioid medication in CRPS remains open. As neuropathic pain does not respond as universally or well as acute nociceptive pain, dose escalation is common, often with no added pain relief but accruing cumulative adverse effects [191–193]. Patients prescribed 100 mg or more of morphine or equivalent have a 9 times greater risk of serious overdose than patients prescribed less than 20 mg of morphine or equivalent daily, even after adjustment for comorbid conditions [194]. There is growing consensus that while at lower doses opioids are a reasonable 2nd or 3rd line treatment option to try, doses should not be escalated freely. Methadone has theoretical advantages for neuropathic pain because of its putative NMDA antagonism, as well as the practical advantage of low cost [195]. Tramadol may be helpful due to its concomitant serotonin/norepinephrine re-uptake inhibition, a potential advantage shared by tapentadol, a potent opioid with noradrenergic reuptake inhibition [196]. Tolerance and long term toxicity are unresolved issues in CRPS patients for the moment, yet it is worth noting that long-term high-dose opioid use can actually worsen allodynia and/or hyperpathia [197–199]. Historically, Mitchell commented on tolerance: “When continuously used, it is very curious that its hypnotic manifestations lessen, while its power to abolish pain continues, so that the patient who receives a half grain or more of morphia may become free from pain, and yet walk about with little or no desire to sleep” [200] reminding us that in some cases low dose opioid can be used successfully, chronically. However, in most cases analgesic tolerance co-occurs with “opioid induced hyperalgesia,” indistinguishable from symptoms of CRPS. Thus there may be a consideration of the use of short-acting opioids for break-through pain (“rescue” dosing), but this has become controversial. Although occasionally taking an extra pill for a pain spike is unlikely to harm, too many patients end up with daily or near-daily use of “rescue” opioids, obviating their purpose and encouraging tolerance to what is effectively a higher daily dose.

The mortality and morbidity of the chronic use of opioids are well known and in a disease that is characterized by hyperalgesia, a drug class that chronically causes hyperalgesia is questionable. It is very important that the risk benefit of such a choice must be continuously assessed.

### NMDA Receptor Antagonists

NMDA receptor antagonists (e.g., MK-801, ketamine, amantadine, memantine, and dextromethorphan) have been evaluated for neuropathic pain, and for CRPS specifically, but toxicity at effective doses has generally been high [201–205]. Ketamine, an NMDA-receptor antagonist has been used topically, orally, intra-nasally, intravenously and intrathecally [206]. Intravenous administration of sub-anesthetic doses has been shown effective in two CRPS RCTs (level 2) assessing either 10 consecutive outpatient infusion treatments [207], or a 4.5 day inpatient treatment with slowly escalating doses [208]. Increasingly, at some centers, low-dose (sub-anesthetic) intravenous Ketamine treatment is provided (level 4), unfortunately in the absence of respectable long-term outcomes data. In our own experience (level 4) tolerance to this treatment can develop, which shortens the time of the full beneficial effect. Additionally, side effects including dysphoria, hallucinations and a drug “high” make this treatment modality unattractive to many patients [209]. High-dose “ketamine coma” is likely associated with very serious side effects and cannot be recommended [210]. There is no clinical trial evidence-base supporting treatment with oral Ketamine. Notwithstanding these results, the cited positive RCTs indicate an important contribution of central sensitization to the CRPS condition which in many patients may be temporarily reversed by treatment with intravenous ketamine [211].

Amantadine has shown some benefit in cancer-related neuropathic pain (level 2) [212] and in chronic neuropathic pain (level 4) [213]. A small RCT assessing oral Memantine together with oral morphine suggested efficacy in a subgroup with persistent upper limb CRPS (40% men, generally low pain intensities) [214] however our subsequent attempt to replicate these results in a more typical group of patients with persistent CRPS provided no indication for any beneficial effect [215].

### Anti-Hypertensives and α-Adrenergic Antagonists

Clonidine is an α_2_-adrenergic agonist used more often in the past to treat CRPS, when “sympathetically maintained pain” was thought to be a more uniform feature than it is now [216] It can be given orally, trans-dermally, or epidurally (level 3 evidence) [217]. Adverse effects include sedation, dizziness, headache, and hypotension [216, 217]. Although a case series showed that transdermal clonidine benefitted local CRPS-induced hyperalgesia and allodynia (level 4 evidence) [218], a systematic review [137] found no convincing support for clonidine (level 1 evidence) [108] and indeed, it is only rarely used for CRPS. Nifedipine, a calcium channel blocker, has a strong mechanistic rationale for managing vasoconstriction (level 4 evidence), and two uncontrolled case series found doses of up to 60 mg/day useful for CRPS [219–221].

Phenoxybenzamine and phentolamine are α-adrenergic antagonists sometimes discussed as third-line agents for CRPS. Two case series provide very preliminary support for efficacy from treatment with phenoxybenzamine [219, 222]. Phentolamine is expensive, in limited supply, and administered by continuous intravenous infusion, and it is not widely used.

### Calcitonin

Calcitonin, a polypeptide hormone produced by the thyroid, appears to have beneficial effects in CRPS independent of its effects on bone. It is usually administered intra-nasally and is without significant adverse effects in normo-calcemic individuals [223]. Calcitonin is one of few CRPS treatments studied in multiple RCTs [224–226], and meta-analysis of a limited number of controlled studies (level 1) demonstrates the value of intranasal doses of 100–300 IU per day for CRPS [227, 228]. Two other clinical trials of calcitonin in CRPS, however, both identified as high-quality studies (level 2) in a systematic review, reported conflicting results [146]. One found improved pain intensity after 100 IU calcitonin thrice daily for 3 weeks; the other reported no improvement after 200 IU calcitonin twice daily for four weeks [224, 226]. Despite the generally positive level 1 type evidence, calcitonin is rarely prescribed, and scarcely available.

### Pharmacotherapy for Other Symptoms in Chronic CRPS

Dystonia, a common movement disorder in CRPS, often requires independent treatment. Dystonia is itself painful and can also worsen pain by impeding tissue perfusion [229]. Treatment is complicated because prolonged tonic postures can allow tendons to shorten into fixed contractures that require (painful, complicating) orthopedic procedures including tendon release or serial casting (see rehabilitation section). Standard treatments for dystonia are usually also prescribed in CRPS, although the mechanisms of dystonia in CRPS and other post-traumatic dystonias are distinct from the dystonias mediated by basal-ganglia dysfunction [229]. Although trihexylphenidate can be considered, baclofen is the current first line option (level 4). It should be prescribed orally at first, but it is sedating and many patients do not tolerate the high oral doses effective for dystonia [229]. If baclofen is effective but poorly tolerated, administration by intrathecal pump is sometimes considered, although pharmacological and mechanical complications are common and the research group that had originally evaluated this intervention has all but abandoned it now (personal communication) [230]. Long-term use of muscle relaxants in CRPS such as benzodiazepines or cyclobenzaprine are empirically ineffective in the long term, as well as poorly tolerated (level 4).

Rare CRPS patients have severe edema in an arm or leg that can painfully distort their tissues and compromise tissue oxygenation and nutrition, potentially leading to skin ulceration, infection (and the extremely rare and extremely controversial) need for amputation in the worst cases [231]. This should be treated with standard treatments, usually limb elevation, regular aerobic exercise to improve circulation, and application of compressive garments if tolerated (see rehabilitation section for detail).

### Emerging Drug Treatment Options

Several emerging treatments are listed above under the immune modulation section. Beyond those agents, there is emerging support for cannabinoids in peripheral and central neuropathic pain, particularly pain associated with multiple sclerosis [232]. Although not yet trialed for CRPS, the emerging trend of state-by-state availability in the USA and legalization in other countries of medical marijuana and cannabinoids improves the feasibility of such a trial [233].

Botulinum toxin type A, used for years to weaken specific muscles in movement disorders and spasticity, works by blocking acetylcholine release at cholinergic synapses [234]. It also inhibits non-cholinergic neurotransmitter (e.g., glutamate) and neuropeptides (e.g., substance P and CGRP) release from primary afferent nerve terminals, providing the rationale for independent evaluation in neuropathic pain [234]. Regional intradermal injections of botulinum toxin improved spontaneous pain, brush allodynia and cold pain thresholds at the painful site of 25 patients with post-traumatic neuralgia [235] and, when used in conjunction with sympathetic blockade with bupivicaine, extended the duration of analgesia in a subset of CRPS patients [236]. (Level 3) These findings await corroboration. Oral CGRP receptor antagonists have been found effective and well-tolerated for acute treatment of migraine [236], and CGRP and other paracrine secretions from nociceptors are thought to perhaps initiate many of the local features of CRPS, so these type drugs should be assessed for CRPS in the future.

### Topical Treatments

Topical treatments must be distinguished from transdermal formulations such as the fentanyl or clonidine patches that deliver systemic medication through the skin. Topical medications remain local, reaching dermal nerve endings, blood vessels, and other cells in the skin. Topical medications are appealing by virtue of their relative lack of systemic effects; rashes and allergies are their only major adverse effect. Topical options to consider for CRPS include the 5% lidocaine impregnated patch, the capsaicin and dimethylsulfoxide (DMSO) (all level 4 for CRPS).

Some clinicians endorse the use Eutectic Mixture of Local Anesthetics (EMLA) for patients with CRPS (level 3 evidence) [237] but it must be applied under an occlusive cover (e.g., plastic food wrap) to maximize penetration. The 5% lidocaine patch is FDA-approved for treating PHN, and is available in generic formulation [238]. It may have efficacy in some local or focal CRPS phenomena such as allodynia (level 4) [239].

Capsaicin, the vanilloid compound in chili peppers, is a highly selective agonist for the Transient Receptor Potential channel, Vanilloid-receptor type 1 (TRPV1) that is expressed on central and peripheral terminals of nociceptive primary sensory neurons [240]. Topical capsaicin causes activation followed by dying-back of nociceptive nerve endings by allowing unchecked cation influx [240]. Use is limited by the painful burning sensation it evokes at the site of application until the site becomes denervated. In an RCT, topical capsaicin showed modest efficacy for PHN (level 2) [241]. A preliminary study of high-dose topical capsaicin plus regional anesthesia for CRPS demonstrated partial efficacy (level 3) [242]. We have found topical capsaicin to be intolerably painful, somewhat messy, and unacceptable to most patients (level 4) [42, 243–246]. In 2009, the FDA approved a high concentration 8% capsaicin patch for treating PHN once every 3 months [247]. It is applied to the painful area for 1 hour *after* topical local anesthesia. Two additional well-powered RCTs (level 2) were positive for high-versus low-dose capsaicin in peripheral neuropathic pain, including in HIV-associated distal sensory polyneuropathy [248, 249]. However, in CRPS, exacerbated pain may render this treatment unsuitable (level 4) [250].

DMSO is a free radical-scavenging agent. In a systematic review (level 1) [251] DMSO (50% cream for 2 months) provided significant CRPS symptom reduction when compared with placebo, however pain intensity was not improved [146].

### Prevention

Analysis and meta-analysis of the first four published studies on the use of vitamin C for prevention of CRPS suggested that vitamin C significantly reduced the likelihood of CRPS developing after limb fracture or surgery [252, 253] with 500 mg vitamin C daily recommended for at least 45 days after injury or surgery [253]. However, a more recent large RCT that used this protocol for the prevention of post-fracture CRPS found that vitamin C was associated with an increased incidence of CRPS at 6 weeks after fracture relative to placebo, with no effect at subsequent time points [254] The potential utility of vitamin C in the prevention of CRPS therefore remains unproven.”

**Figure 3.**
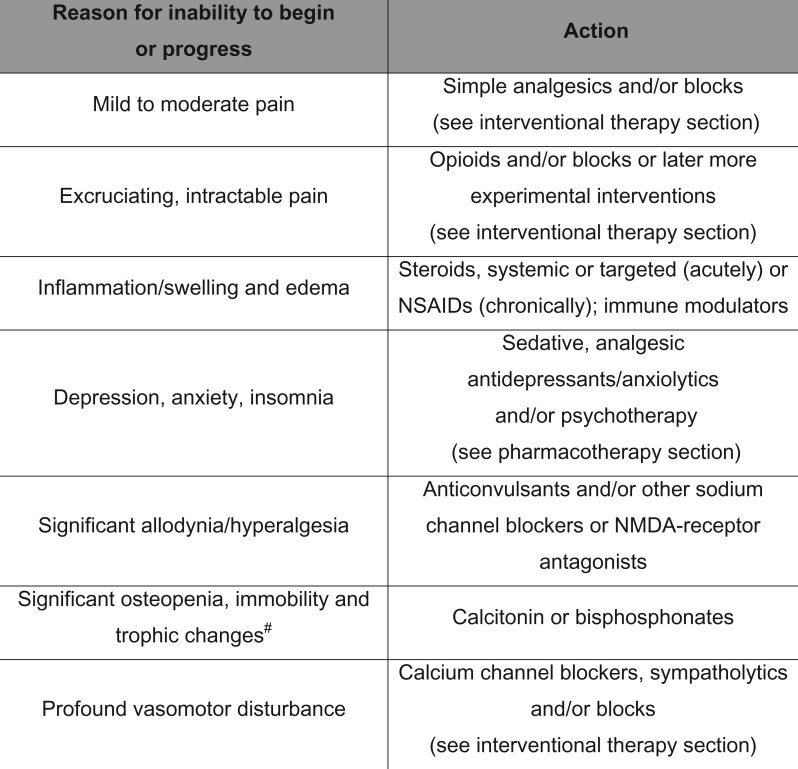
An empiric, consensus-based pharmacotherapy guide (modified by consensus from [3]). The following strategies are suggested for patients who have been diagnosed with CRPS but who cannot begin or progress in the functional restoration algorithm (level 4)*.*It is important to remember that these general suggestions are overruled by individual patient presentation.^#^It is important to note that certain drugs (e.g. calcitonin), may be associated with analgesia as well as the more primary action.

To summarize, there are few therapeutic drug trials in CRPS patients that meet criteria for level 1 or level 2 evidence. Clinicians must thus be guided by the results of RCTs for neuropathic pain, smaller specific CRPS trials, and clinical experience. A methodical and patient-centered empirical approach is essential (see Figure 3). New drugs should be trialed one at a time, up to maximum dose, and discontinued if not clearly helpful or where adverse effects are intolerable. The goal is as much to allow progress in functional restoration and rehabilitation as to relieve pain.

## Psychological Interventions

Clinicians who work with CRPS patients recognize that successful management of the syndrome presents a significant challenge. In the absence of any definitive medical treatment [51, 227] the need for interdisciplinary management of CRPS has been emphasized as above [1, 58, 255]. It is now generally agreed that successful treatment must simultaneously address the medical, psychological, and social aspects of the syndrome [1, 53, 58]. As will be described below, there are several reasons why addressing psychological and behavioral factors may be crucial to successful treatment in patients with CRPS. A rationale for use of psychological interventions in the management of CRPS will first be described. The treatment outcome literature regarding efficacy of psychological interventions for CRPS will then be presented, followed by a brief overview of relevant meta-analytic literature regarding efficacy of such interventions for non-CRPS chronic pain conditions. Finally, an overview of clinical recommendations for psychological care of CRPS patients based on both research literature and clinical experience will be presented.

### Hypothesized Links Between CRPS and Psychological Factors

The rationale for employing psychological interventions in CRPS patients derives generally from their recognized utility in management of non-CRPS chronic pain conditions, and more specifically, from theoretical pathways through which psychological and behavioral factors *might* directly interact with pathophysiological mechanisms believed to underlie CRPS. This latter theoretical rationale suggests the possibility that psychological interventions may not only be palliative in CRPS (which is almost assured) but also could have a *potentially* beneficial impact on underlying pathophysiology of the disorder in the context of interdisciplinary treatment.

One pathway through which psychological factors could influence onset or maintenance of CRPS relates to the role of adrenergic mechanisms in the pathophysiology of CRPS (see Bruehl [256, 257] for a review of pathophysiological mechanisms of CRPS). Diminished sympathetic outflow following peripheral nerve injury is believed to lead to localized upregulation of peripheral catecholaminergic receptors in the affected extremity [258–260]. This upregulation may lead to local hypersensitivity to circulating catecholamines, which in turn leads to excessive vasoconstriction [258–262], accounting for the characteristic cool, blue extremity typically seen in chronic CRPS. Following nerve injury like that which may initially trigger CRPS [263, 264], primary afferent fibers may also become sensitive to adrenergic excitation, leading to increased nociceptive firing in response to sympathetic discharge or circulating catecholamines [265–267]. This catecholamine-induced nociceptive firing in turn is likely to contribute to central sensitization (by maintaining elevated peripheral nociceptive input) which may underlie the allodynia and hyperalgesia associated with CRPS [268, 269]. Central sensitization produces increased pain, which itself may provoke catecholamine release that further stimulates the nociceptive input maintaining the central sensitization, thereby producing a dysfunctional vicious cycle. The impact of catecholamine release in the pathophysiological mechanisms described above is important to recognize given that psychological factors such as life stress and emotional distress (e.g., anxiety, anger, depression) can be associated with increased catecholamine release [270, 271]. For example, greater depressive symptoms were associated with higher levels of plasma epinephrine in a sample of 16 CRPS patients [131]. It is theoretically plausible that psychological factors such as these could, through their impact on catecholamine release, interact with adrenergically mediated pathophysiological mechanisms to contribute to maintenance, or possibly onset, of CRPS.

More recent work suggests that interactions between psychological factors and inflammatory mediators may also be important to consider, given the increasingly recognized role of inflammation in CRPS [256]. For example, laboratory research in healthy individuals indicates that greater pain-related catastrophic thinking, which is common in CRPS patients, is associated with increased pro-inflammatory cytokine activity in response to painful stimuli [272]. Moreover, in CRPS patients, psychological stress has been shown to be associated with alterations in immune function that could impact on inflammatory cytokines hypothesized to contribute to CRPS [273]. Thus, psychological stress, catastrophizing, and negative affect variables associated with an elevated pro-inflammatory state could exacerbate any underlying inflammatory mechanisms contributing to CRPS.

Examination of the historical CRPS literature indicates frequent comments from authors indicating that psychological dysfunction (usually emotional disorders) was assumed to contribute to CRPS in many patients. This assumption often colored physicians’ conceptualization of CRPS patients despite the absence for many years of controlled studies testing these assumptions. Examination of this literature indicates that most studies assessing the role of psychological factors in CRPS have been limited to case series descriptions or cross-sectional psychological comparisons between CRPS patients and non-CRPS chronic pain patients. A 2009 review of this literature concluded that the majority of these studies do not support a role for psychological factors in onset and maintenance of CRPS [274].

Ability to make conclusions about psychological factors *contributing to onset* of CRPS depends on a prospective research design, and unfortunately, well-designed prospective studies are rare in the CRPS literature. A prospective study in 50 post-fracture patients indicated that while occurrence of CRPS was relatively common (18% incidence), personality and depression scores did not differ significantly between those who did and did not develop CRPS [275]. Similar but stronger conclusions can be drawn from a large, well-designed prospective study of 596 consecutive fracture patients, of whom 7% developed CRPS [276]. Neither depression nor stressful life events assessed shortly after fracture predicted eventual development of CRPS. In contrast to these negative findings, other prospective work indicates that higher levels of anxiety prior to undergoing total knee arthroplasty were associated with significantly greater likelihood of a CRPS diagnosis at one month post-surgery, with a similar trend for depression [277]. Subsequent findings in this dataset provide stronger evidence in support of the psychophysiological model described above [33]. Using the extent of CRPS symptoms indexed by the CSS (described above) as the outcome rather than dichotomous diagnosis, increases in depression levels from pre-surgical baseline to 4 weeks post-surgery were found to predict significantly greater extent of CRPS symptoms at both 6- and 12-month follow-up, with similar findings at 6-months for early post-surgical increases in anxiety [33].

The best available literature above is ambiguous. However, even if the psychophysiological model were accurate, this should not be taken to imply that the presence of psychological “risk factors” alone would be either necessary or sufficient to cause CRPS. For example, another prospective study revealed that among 88 consecutive patients assessed shortly after acute distal radius fracture, 14 had significantly elevated life stress but did not develop CRPS, and the one patient who did develop CRPS had no apparent psychological risk factors (i.e., no major life stressors, average emotional distress levels) [278].

Until more definitive prospective studies are available, the question of whether psychological factors affect the development and maintenance of CRPS must be addressed solely on the basis of case reports and retrospective or cross-sectional research designs which do not allow causation to be inferred. Two uncontrolled retrospective case series reported a relationship between onset of CRPS and contemporaneous emotional loss or major life stressors [279, 280]. Similarly, a controlled study regarding the role of life stress in CRPS onset found that 80% of patients in a CRPS sample recalled a stressful life event contemporaneous with the initiating physical trauma, in contrast to only 20% of non-CRPS controls [281]. While one retrospective study indicates that pediatric CRPS patients also recall a higher level of stressful life events at pain onset compared to non-CRPS pediatric pain patients [282] and other work indicates greater rates of Post-Traumatic Stress Disorder diagnosis in adult CRPS patients than in non-CRPS chronic pain patients (with onset predating chronic pain development in 86% of patients) [283]; all of these studies are retrospective in nature. There remain no prospective tests of this life stress hypothesis. In contrast to the positive findings above, one cross-sectional study indicated that while CRPS patients reported stressful life events at a higher rate than in the general population, they reported *fewer* stressful life events than individuals with conversion disorders or affective disorders [284]. Moreover, rates of childhood traumatic experiences were similar between CRPS patients and those with affective (e.g., depression) or conversion disorders. Results of this latter study do not provide strong support for a unique role of stressful life events in CRPS development.

If psychological dysfunction were somehow uniquely involved in onset or maintenance of CRPS, one might also expect increased prevalence of psychiatric disorders or elevated levels of emotional distress in this population. Based on structured interviews, estimates for prevalence of Axis I psychiatric disorders (e.g., anxiety and depressive disorders) in CRPS patients indicate a prevalence ranging from 24% to as high as 46% [285, 286]. It should be noted that only Monti et al. [285] included a non-CRPS chronic pain control group, and these authors reported that Axis I prevalence was not significantly higher in CRPS compared to non-CRPS pain patients. Neither of the studies above documented psychiatric status *prior to* CRPS onset, and therefore cannot address the issue of causality. Possibly arguing against depressive disorders as a unique contributor to CRPS onset is recent work indicating that depression levels in a sample of adult CRPS patients, although higher than in other types of chronic pain, were significantly lower than in patients with Major Depressive Disorder [287]. In summary, there is currently no compelling evidence that psychiatric disorders contribute to development of CRPS, nor is there consistent evidence that CRPS patients suffer from diagnosable psychiatric disorders at a higher rate than do other chronic pain patients. Of course, CRPS patients should be carefully assessed for psychologic disorders, and if found, should be treated definitively for optimal outcomes.

Controlled studies have also addressed the issue of whether CRPS patients are more emotionally distressed than other types of chronic pain patients. Several cross-sectional studies have found that CRPS patients report being more emotionally distressed than non-CRPS pain patients, in terms of depression and/or anxiety levels [287–290]. Other work indicates that patients displaying signs and symptoms of CRPS 6 months following total knee replacement reported significantly higher levels of anxiety than did patients not displaying CRPS, despite the fact that both groups were continuing to experience at least some degree of pain [277].

It is not known whether observed elevations in psychological distress in studies like those above are a cause or result of CRPS pain. Possibly in support of the former causal interpretation are data from a time series diary study indicating that depression levels on a given day were a significant predictor of CRPS pain intensity on the following day [291], a finding common in non-CRPS chronic pain as well (e.g., Burns et al. [292]). The other alternative, however, is that elevated distress sometimes reported in CRPS patients relative to non-CRPS chronic pain patients might be due to the unusual and sometimes severe and dramatic symptomatology of CRPS (e.g., allodynia, hyperalgesia, vasomotor changes, significant edema, motor changes) being more distressing than experiencing more common forms of chronic pain.

Despite results of some studies suggesting that CRPS patients are more distressed than comparable non-CRPS chronic pain patients, several other studies have reported no such differences. For example, work by Ciccone and colleagues provided only partial support for this hypothesis, finding that CRPS patients reported more somatic symptoms of depression than non-CRPS patients with local neuropathy, but displayed no emotional differences relative to low back pain patients [293]. Other studies have found no evidence of elevated distress among CRPS patients compared to low back pain patients [294, 295] or headache patients [294]. These negative results suggest the possibility that rather than CRPS being associated inherently with greater distress, the inconsistent findings regarding this issue may be accounted for by differences in sample selection, pain duration, clinic referral patterns, and specific psychometric measures used across studies. In the absence of additional well-controlled studies, it remains unclear whether the findings suggesting uniquely elevated distress in CRPS patients are an artifact of sample selection.

Whether or not absolute levels of negative affect are elevated in CRPS patients, several studies suggest that negative affect, when present, may have a greater impact on pain intensity in CRPS than in other types of chronic pain [290, 296]. Specifically, correlations between pain intensity on the one hand, and depression, anxiety, anger expressiveness, and acute mental stress on the other hand, have been found to be significantly stronger in CRPS patients than in non-CRPS chronic pain patients [290, 296–299]. These results suggest that even if CRPS patients are not uniquely distressed, the impact of that distress may be unique, possibly due to the hypothesized adrenergic interactions described above. Such findings may also have treatment implications. For example, a small prospective treatment study in CRPS patients indicated that greater baseline anxiety predicted lower subsequent pain relief and functional improvement 6-months or more following treatment using sympathetic blocks (level 3) [300]. Conversely, psychological interventions that reduce distress might be expected to contribute to reductions in CRPS symptoms (e.g., pain, vasomotor changes) and potentially enhance the efficacy of other interventions.

Another important pathophysiological mechanism that may contribute to CRPS is the sometimes dramatic disuse that patients develop in an effort to avoid stimuli that may trigger hyperalgesia and allodynia in the affected extremity. The impact of disuse is demonstrated by an experimental study in 30 healthy individuals who underwent upper extremity casting for 28 days. Compared to non-casted controls, experimental immobilization alone resulted in cold hyperalgesia and skin temperature asymmetry lasting 3 days following cast removal, as well as longer lasting reductions in mechanical pain threshold [84]. That disuse is an issue in CRPS is supported by findings that diminished active range of motion is common even in early CRPS [301], and that CRPS is associated with significantly reduced mobility and impaired ability to use the affected area normally [302]. Significant inverse correlations between CRPS pain intensity and ability to carry out activities of daily living [303] suggest that pain avoidance is a likely reason for CRPS-related activity impairments and disuse. Learned disuse (kinesiophobia), related to fear of pain and reinforced by either avoidance of actual pain or reduced anxiety subsequent to avoiding *anticipated* pain exacerbations, may prevent desensitization and eliminate the normal tactile and proprioceptive input from the extremity that may be necessary to restore normal central sensory processing [1, 45]. Learned disuse may also inhibit the natural movement-related pumping action that helps prevent accumulation of catecholamines, pronociceptive neuropeptides, proinflammatory cytokines and edema in the affected extremity, all of which may impact negatively on CRPS signs and symptoms [265, 304]. Pain-related learned disuse might therefore interact with other pathophysiological mechanisms to help maintain and exacerbate both the pain-related and autonomic features of CRPS [305].

While the contribution of psychophysiological interactions to CRPS is largely speculative, it is theoretically consistent and highlights the importance of addressing psychological factors in the clinical management of CRPS. A vicious cycle in which pain provokes disuse and emotional arousal, both of which in turn further exacerbate the pain, could contribute to maintenance of CRPS. Psychological/behavioral treatments may therefore play an important role in CRPS management by targeting learned disuse and both life stress and negative affect that may contribute to maintenance or exacerbation of the disorder. Consistent with potential benefits of this treatment focus, a prospective study (level 3) in acute CRPS patients found that greater baseline anxiety and pain-related fear predicted worse treatment outcomes in terms of pain and disability over the following 12 months [306], suggesting that early targeting of these issues may have long-term benefits. Psychological treatments can also enhance pain coping skills that ultimately lead to improved functioning and quality of life and increase ability to self-manage pain. In line with this, an electronic diary study showed that in CRPS patients, greater engagement in pain acceptance-based coping (a core tenet of Acceptance and Commitment Therapy [ACT]) was linked to same-day improvements in pain and mood [307]. At minimum, psychological treatments focusing on the issues above are likely to enhance patients’ sense of control over the condition, and thereby reduce fears that may be a barrier to achieving success in functional therapies.

### Efficacy of Psychological Interventions in CRPS Patients

A PubMed literature review reveals a number of studies that have addressed efficacy of psychological interventions for CRPS, although nearly all of these reflect uncontrolled designs that permit only limited conclusions to be drawn. An additional caveat regarding these studies is that the criteria used to diagnose CRPS were often not adequately described and in all likelihood varied substantially across studies. This lack of consistent or specified diagnostic criteria limits the ability to generalize these results to patients diagnosed according to current IASP criteria for CRPS.

A summary of studies specifically reporting on efficacy of psychological treatments for CRPS is presented in Appendix A1 [314–329]. This reveals few randomized controlled trials (RCTs) specifically testing psychological interventions in CRPS patients. Fialka et al. [315] (level 2) randomized 18 CRPS patients to receive either home PT or home PT plus once-weekly autogenic relaxation training for ten weeks. Both groups showed similar improvements in pain, range of motion, and edema, although patients in the PT+Autogenics group demonstrated significantly greater improvements in limb temperature. Although low statistical power due to the small sample limited the ability to adequately evaluate intervention efficacy, these results suggest that relaxation-based interventions may have some benefit in management of CRPS.

Paced breathing in various forms is often a component of biofeedback, relaxation, and newer mindfulness-based interventions for pain. Results of one well-controlled pilot study (level 3) found that while healthy individuals experienced significant improvements in cardiac vagal tone (an index of inhibitory neural tone based on heart rate variability) when engaging in paced breathing, CRPS patients did not, despite achieving comparable reductions in breathing rate [318]. Despite absence of changes in this vagal index with slowed breathing, the pain relevance of vagal inhibitory function in CRPS was shown by its strong inverse association with pain intensity in the CRPS group [318]. Although not definitive due to low statistical power, these pilot findings raise the possibility that physiological alterations associated with CRPS might impair the efficacy of some relaxation-focused interventions.

Although there are no well-controlled trials of traditional biofeedback training (e.g., relaxation training or autogenic training combined with finger temperature or muscle tension biofeedback) for CRPS, there is one study describing a novel use of virtual reality that can be viewed as a form of biofeedback [321]. In a sham-feedback controlled crossover trial (level 2), presentation of a virtual reality image in which the affected limb was flashing visually in synchrony with the heartbeat significantly reduced pain intensity, increased grip strength, and increased vagal cardiac tone (based on heart rate variability) compared to an image of the limb flashing out of synchrony with the heartbeat [321]. While intriguing, it is unclear whether this technique would be pragmatic in a clinical setting due to the technology involved.

Another application of behavioral therapy for CRPS management noted previously is graded exposure therapy, an intervention that directly targets pain-related fears and learned disuse. In an initial trial of this intervention, in vivo graded exposure therapy was used to target fear of movement in eight CRPS patients in a series of well-controlled single subject experiments (level 3 evidence) [104]. This exposure therapy resulted in significant reductions in pain-related fear of movement, with pain, disability, and other symptoms of CRPS also decreasing significantly in parallel fashion. A subsequent RCT (level 2) showed that compared to treatment as usual (pain-contingent therapy), in vivo exposure led to significantly greater improvements in pain intensity, pain catastrophizing, perceived harmfulness of activities, and disability at 6 month follow-up [320]. These results are consistent with findings based on per protocol (but not intent-to-treat) analyses in a separate RCT (level 2) [319]. Recent work suggest that exposure therapy may be more effective for CRPS when it targets a greater variety of feared activities [322].

Results of several published case studies and small case series suggest that the pain of CRPS may also be reduced through use of a variety of other psychological techniques. For example, Barowsky et al. [310] (level 4) reported on a 12-year old CRPS patient in whom ten sessions of thermal biofeedback resulted in resolution of CRPS that had been resistant to previous treatments. Alioto [309] (level 4) reported that an adult chronic CRPS patient experienced a 75% decrease in pain intensity and improved mood following a series of psychological sessions incorporating autogenic relaxation, breathing relaxation, and muscular and temperature biofeedback. Total elimination of pain was reported by this same author in a 16-year old CRPS patient using a similar intervention approach [309]. Dramatic improvements like those above were also noted in an adult chronic CRPS patient described by Blanchard (level 4) [308]. Eighteen sessions of thermal biofeedback training resulted in nearly complete elimination of pain, as well as the ability to raise digital temperature in the affected hand by 1.5 degrees C. This relief was reported to be maintained at one-year follow-up. Autogenic relaxation and imagery training (six sessions) have been reported to result in complete resolution of CRPS-related pain of seven months duration in a 15-year old patient, with these gains reportedly maintained at 18 month follow-up (level 4) [311]. Hypnotic imagery combined with relaxation techniques (over a six to nine month period) has additionally been reported to result in complete resolution of CRPS symptoms in a series of three adult CRPS patients (level 4) [313]. It should be noted that the complete resolution of symptoms described in some case reports using only psychological interventions is likely to be atypical, and fails to recognize the number of less dramatic successes or even treatment failures no doubt encountered by these same authors. While the uncontrolled designs used in the studies described above prevent definitive conclusions from being drawn regarding the efficacy of psychological techniques for CRPS, they clearly support the idea that such techniques can play an important role in effective multidisciplinary treatment.

Other research also supports the value of integrating psychological methods into multidisciplinary CRPS management [64, 103, 316, 317]. Two RCTs examining efficacy of physical therapy for CRPS described previously have included components of psychological treatment in the therapy package [64, 72, 316]. Oerlemans et al. [64, 316] (level 2) tested a physical therapy protocol that included relaxation exercises and cognitive interventions (designed to increase perceived control over pain). This combined intervention was found to produce significantly greater improvements in pain, active range of motion, and impairment levels than were observed in the social work control group [64, 316]. In another RCT of physical therapy, Lee et al. [72] (level 2) examined two different frequencies of physical therapy treatment (once per week versus three times per week) for child and adolescent CRPS patients, with both groups additionally receiving six sessions of cognitive behavioral treatment. Although no attentional control group was available for comparison, both groups were found to improve significantly in terms of pain and function when compared to their pre-treatment baselines. While the multicomponent interventions in both of these studies do not permit conclusions to be drawn specifically regarding the efficacy of psychological interventions, they do suggest that psychological treatment in combination with physical therapy may prove effective in a rehabilitation-focused approach to management of CRPS. This conclusion is also supported by results of a prospective controlled case series examining efficacy of combined therapy for CRPS. A 4-week interdisciplinary pain management program including medical treatment, physical and occupational therapy, and group psychotherapy produced significant improvements in several functional outcomes without any corresponding increases in pain-related anxiety, suggesting how such treatments could potentially work synergistically (level 3) [317]. Although not addressing CRPS specifically, one recent meta-analysis (level 1) suggests that psychological interventions for chronic pain may be more effective when provided in the context of multidisciplinary care than when provided alone [323]. This meta-analysis found that effect sizes for an ACT intervention approach regarding disability and mood were significantly larger when ACT was provided in the multidisciplinary rather than unidisciplinary context.

Uncontrolled trials also support inclusion of psychological interventions in the multidisciplinary treatment package for both pediatric and adult CRPS patients. Wilder et al. [314] (level 3) described a conservative multidisciplinary treatment program used in 70 childhood CRPS patients that incorporated relaxation training and cognitive-behavioral interventions, noting that it resulted in improved pain and functioning in 57% of the sample. Even more impressive results were reported by Sherry et al. [103] (level 3) in a case series of 103 primarily adolescent CRPS patients. Multidisciplinary treatment incorporating conservative medication management, regular active physical therapy, and psychological counseling (for 77% of the sample) reportedly resulted in 92% of this sample achieving symptom-free status [103]. Although no details are provided, Wesdock et al. [312] (level 3) noted that biofeedback was helpful in some cases of short-duration childhood CRPS in the context of multidisciplinary treatment. In an adult CRPS case series with 49 patients (level 3), a multidisciplinary treatment program including individual biofeedback training and both group and individual cognitive behavioral therapy resulted in significant pre-post treatment improvements in mood, catastrophizing, pain coping, pain acceptance, and pain-related disability, as well as improvements in pain intensity that approached significance [86]. A smaller case series (level 3) in 10 adult CRPS patients also suggested benefits of multidisciplinary treatment that incorporated cognitive-behavioral therapy and ACT, showing notable pre-post intervention reductions in specific sensory (18%) and motor/trophic (19%) symptoms of CRPS [324].

Given the nearly complete absence of RCTs of psychological interventions for CRPS, results of a recent review and meta-analysis of cognitive behavioral interventions in other neuropathic pain patients may be informative [325]. Only a single randomized controlled trial of high methodological quality was identified, which demonstrated significant efficacy of cognitive behavioral interventions for reducing neuropathic pain intensity, although this effect was restricted to women (level 2) [326]. However, meta-analysis of all four available controlled trials (level 1) indicated no overall significant effects of cognitive behavioral therapy on neuropathic pain intensity. These results do not provide unambiguous support for the likely efficacy of psychological interventions in CRPS patients, but firm conclusions cannot be drawn due to the limited number of studies available.

In summary, there are only a few small RCTs specifically testing the efficacy of psychological interventions for CRPS, either alone or in the multidisciplinary context. However, the data available do suggest that psychological interventions are likely to be a useful part of a comprehensive interdisciplinary treatment program. The efficacy of such techniques for CRPS would not be surprising, given the strong evidence of their utility in other types of chronic pain. These results will be briefly summarized below.

### Comparative Efficacy of Psychological Interventions in Other Non-CRPS Chronic Pain Disorders

Numerous RCTs have documented the efficacy of various psychological approaches to the management of chronic pain in general, and these have been quantitatively summarized in several published meta-analyses. Treatment approaches examined include many of the same interventions used in the CRPS studies described previously, including relaxation techniques, autogenic training, biofeedback, behavioral therapy, and cognitive behavioral therapy, as well as more recent mindfulness-based and ACT approaches. Results of several meta-analyses clearly document the efficacy of these techniques for non-CRPS chronic pain conditions. For example, a meta-analysis of clinical trials testing progressive muscle relaxation techniques found significant effects in various chronic pain conditions, reflecting a moderate effect size (level 1) [327]. Meta-analysis specifically of autogenic training, another relaxation procedure, also indicated a significant and at least moderate effect size in controlled trials for patients with headache and somatoform pain disorder (level 1) [328]. Significant efficacy for biofeedback training is also indicated by meta-analyses in populations including temporomandibular joint pain and migraine headache patients (both level 1) [329, 330]. More generally, meta-analyses of RCTs across psychological treatment types (various treatments provided both alone and in combination) indicate significant efficacy of this class of techniques for a variety of chronic pain conditions, including low back pain, fibromyalgia, rheumatoid arthritis, and cancer-related pain (all level 1) [331–341]. Results of one available meta-analysis also confirm that cognitive behavioral interventions are significantly effective for children and adolescents with chronic pain (level 1) [342]. Overall, the results of RCTs of psychological treatment approaches consistently indicate at least a moderate benefit, in terms of experienced pain, mood, and function, for patients with a variety of chronic pain conditions. Given the efficacy of these interventions shown for various non-CRPS chronic pain conditions, their utility specifically in the management of CRPS might also be expected. These meta-analytic findings provide additional support, albeit indirect, for the reported efficacy of psychological interventions in CRPS patients described in uncontrolled trials.

### Clinical Recommendations

There is little well-controlled CRPS-specific outcome research on which to base psychological treatment recommendations for the condition. However, clinical experience and available data do suggest several specific strategies that may be helpful.

While there are indications that many cases of acute CRPS may resolve relatively quickly without any need for specific psychological intervention, a low cost and potentially helpful intervention recommended for all *acute or chronic* CRPS patients is comprehensive education about the condition [343]. Specifically, it is recommended that all patients *and their families* receive detailed information early in treatment that addresses the negative effects of disuse, the importance of reactivation, the need for an active self-management approach to treatment, and that provides an explanation of how possible psychophysiological interactions could affect severity of CRPS. Such education may help prevent development of dysfunctional behavior patterns (e.g., elevated distress and severe disuse) that could contribute to the severity, disability, and chronicity of the condition. For more chronic CRPS patients or those who do not respond to limited intervention, individualized psychological evaluation is recommended, followed by focused psychological pain management treatment. An overview of several key issues to address in this assessment and treatment is provided below.

#### Psychological Assessment

Several specific areas of relevance to CRPS management should be addressed in the psychological evaluation, including: 1) presence of comorbid Axis I (or Axis II) psychiatric disorders, 2) cognitive, behavioral, and emotional responses to CRPS, 3) ongoing life stressors, and 4) responses by significant others to the patient’s CRPS. As noted previously, Axis I psychiatric disorders such as Major Depression, Panic Disorder, Generalized Anxiety Disorder, and Posttraumatic Stress Disorder are at least as common in CRPS patients as in other chronic pain patients [285]. The importance of assessing for disorders such as major depression is highlighted by the fact that diminished energy level and motivation related to clinical depression may be a significant barrier to success in active physically-focused treatment modalities (e.g., physical and occupational therapy); also, there are very effective and safe medications available. Identification of specific life stressors and general emotional arousal (depressed, anxious, fearful, or angry mood) even in the absence of clinically diagnosable psychiatric disorder may be equally important given possible psychophysiological interactions hypothesized above.

Research in chronic back pain patients indicates that pain-related disability is more strongly related to *fear* of pain than it is to the level of pain intensity itself [49]. Therefore, assessment of CRPS patients’ fear of their pain is also important. Evidence from studies in chronic back pain patients indicates that pain-related fear contributes to elevated pain intensity and disability in part by leading to chronic guarding, bracing, and disuse in response to fears that movement will lead to increased pain and re-injury [344]. This is particularly important for CRPS patients, in whom disuse may interact directly with the pathophysiology of the disorder, and in whom severe guarding may contribute to secondary proximal myofascial pain that can mimic spreading of the disorder (and further increase fear). Not all activity avoidance in CRPS patients is unreasonable (e.g., avoiding heavy lifting with the affected hand), and therefore the focus should be on identifying activity avoidance that is extreme and unreasonable. For example, some CRPS patients may appear to be experiencing agoraphobia based on their reports of an intense desire to avoid crowded environments. However, further assessment in these cases may reveal that this avoidance is motivated by excessive fears that someone will accidentally make contact with the affected extremity and provoke extreme pain. While patients admit that this is unlikely to occur, the behavior persists. This pattern highlights the fact that activity avoidance and disuse in chronic pain can be operantly-reinforced by the decreased fear that accompanies avoidance of expected pain exacerbations [345].

Assessment of the cognitive impact of CRPS should include thorough exploration of the patient’s beliefs regarding CRPS. Several misconceptions are common among patients, particularly those who have failed previous treatments. For example, patients may believe that CRPS is an untreatable, progressively deteriorating condition, and that it will necessarily spread throughout the body (a belief not supported by empirical studies). Catastrophic cognitions such as these are often a contributor to negative emotional states that may have a deleterious impact on CRPS and responses to treatment [300]. The importance of addressing catastrophic cognitions in CRPS treatment is highlighted by results of a prospective study in non-CRPS neuropathic pain patients, which indicated that level of catastrophizing at study baseline predicted level of pain eight weeks later, independent of baseline pain and depression [346]. Patients may also possess incorrect beliefs regarding the meaning of CRPS pain. Not surprisingly given the intensity and unusual nature of allodynic pain, patients may assume that pain signals damage, and as a corollary, “if it hurts, don’t do it.” Such beliefs may be a primary contributor to pain-related fear, and consequently, exacerbate disuse (kinesiophobia). It is therefore important that patients understand that CRPS pain does not signal tissue damage. Unrealistic beliefs regarding how CRPS treatment should progress may also be problematic. Common misconceptions include beliefs that sympathetic blocks alone are curative, and that treatments that exacerbate pain temporarily cannot contribute to long-term improvements. Invasive and expensive interventional procedures, such as spinal cord stimulation, may prove valuable for some patients in the later stages of treatment. However, excessive focus early in treatment upon invasive interventions viewed as a “quick fix” before patients have participated in a comprehensive interdisciplinary/multidisciplinary program leads to reduced motivation to engage actively in such care, and outcomes are likely to suffer. The importance of considering treatment expectations is underscored by recent qualitative research examining the content of CRPS internet message boards, which found that many CRPS patients have unrealistic expectations regarding likely outcomes of medical interventions for CRPS [347].

#### Psychological Pain Management Interventions

The pain management intervention component of CRPS treatment should include relaxation training (preferably in conjunction with thermal and/or electromyographic biofeedback) and/or mindfulness-based stress reduction, training in cognitive pain coping skills (CBT), related interventions focused on living well with CRPS (i.e., ACT), and behavioral intervention to address disuse and activity avoidance issues, as well as family reinforcement issues. In addition to the above, other targeted cognitive behavioral therapy interventions may be helpful if specific issues are identified during evaluation which may impact on the condition or ability to engage effectively in treatment (e.g., major ongoing life stressors or Axis I psychiatric disorders).

The goal of relaxation training with biofeedback is to increase patients’ ability to control their pain and decrease emotional arousal (and associated sympathetic discharge) that may impact negatively on the condition. Clinical trial data in non-CRPS chronic pain suggest that breathing-focused relaxation, progressive muscle relaxation, relaxing imagery, autogenic training, and mindfulness-based approaches all may prove beneficial. There is no clear evidence of the superiority of any one of these interventions, and thus the specific techniques employed are generally determined by patient and therapist preference. With all relaxation/biofeedback techniques, the key factor determining their clinical efficacy is the degree to which patients practice the techniques at home and integrate them into their pain coping during regular activities on a daily basis.

A second aspect of the pain management treatment component is cognitive intervention. Given the emphasis in consensus guidelines for CRPS management using an active rehabilitation approach [1, 53], it is important to reframe the CRPS patient’s role as that of an active participant in the treatment process rather than a passive recipient of treatment interventions. As part of this active treatment focus, pain exacerbations should be identified as a cue to practice self-management interventions that may help the patient gain control over their situation. As patients learn relaxation skills and begin to understand the cognitive and behavioral aspects of the syndrome, they will have increasing resources for exerting at least some degree of control over their CRPS. Increased sense of perceived control, even if that control is limited in scope, may be an important factor in determining outcomes in chronic pain treatment [e.g., Andrasik and Holroyd [348]). Dysfunctional cognitions may be common in CRPS patients [290], including catastrophic interpretations about symptoms or implications of CRPS for the future, fearful pain-related cognitions like those described above, and unrealistic beliefs about treatment. Such cognitions contribute to elevated distress, which may impact sympathetic outflow and catecholamine release, and potentially aggravate CRPS pain and vasomotor changes. Moreover, in the absence of in vivo reactivation experiments in which constructive (i.e., encouraging rather than catastrophic) self-talk is practiced, fear of pain may prevent improved daily function even in the face of objectively improved capabilities during therapy. It is therefore important that cognitive interventions be employed to help patients learn to identify and modify their specific dysfunctional cognitions regarding reactivation, CRPS, and its treatment.

Evidence in non-CRPS pain conditions also suggests that targeting acceptance of CRPS may enhance pain coping and quality of life in CRPS patients. ACT is considered a next generation CBT intervention, and it focuses on helping patients engage in flexible patterns of behavior that increase engagement in valued life activities despite continuing pain and discomfort [349]. Given the dearth of proven medical interventions for CRPS and its sometimes intractable nature, interventions such as ACT that target learning to live more effectively with the condition are likely to prove valuable.

Given the impact of learned disuse as a potential barrier to reactivation, behavioral interventions targeting this disuse can also be an integral component of the overall treatment program. Reactivation and behavioral goals must necessarily balance disuse concerns with avoiding *severe* pain exacerbations that could potentially contribute to maintenance of CRPS and reinforce learned disuse. *Realistic* pain-limited incremental reactivation is key, with the psychologist and functional therapists coordinating efforts to ensure that appropriate activity goals are set and that problems encountered in this reactivation process (e.g., pain-related fear of movement) are effectively addressed. As noted above, there is some experimental evidence supporting the efficacy of graded in vivo exposure therapy to address pain-related fear in CRPS, with apparent beneficial effects on pain and other CRPS symptoms as well [104].

With regards to family intervention, the most crucial issue to address is the possibility that some family members may be a barrier to reactivation due to solicitous responses and fear of pain exacerbations. Unless detailed education regarding CRPS and disuse issues is provided, family members may consider any activity that increases pain as dangerous to the patient and something to be discouraged. It is therefore important to ensure that family members understand the necessity of reactivation and that this might be associated with transient increases in pain. In contrast, family members may, due to a lack of understanding, incorrectly assume that unusual symptoms such as allodynia are exaggerated, and as a consequence, be less than fully supportive. Adequate positive family support can have a significant impact on ultimate efficacy of treatment. Family members should therefore be guided in how they can best respond to the patient’s pain in a way that encourages and facilitates appropriate reactivation, and helps keep the patient focused on constructive management of the condition. The importance of addressing family issues is highlighted by findings demonstrating that more than half of caregivers of CRPS patients experience negative mood and significant strain, and these factors in turn are associated with greater patient disability [350]. While one might assume that this family distress and strain is a *result* of having to handle greater patient disability, the possibility of bi-directional causal influences must at least be considered.

### Summary of Psychological Considerations

There is no compelling evidence that psychological factors are necessarily involved in the cause of chronic CRPS. However, there are theoretically plausible pathways through which psychological factors in some cases *could* affect the development of CRPS. Psychological factors are usually not the *cause* of the disease, but are very often an *effect* of the disease. There is also no consistent experimental support for the idea that CRPS patients are in any way psychologically unique compared to other chronic pain patients. Once CRPS has developed, however, emotional factors may have a greater impact on CRPS pain intensity than in non-CRPS pain conditions, possibly through the impact of negative affective states on catecholamines. Meta-analytic reviews document the efficacy of various psychological interventions for many types of non-CRPS chronic pain, and suggest that such interventions are likely to be beneficial for CRPS patients as well. Adequate RCTs of psychological interventions in CRPS patients are not available to guide this aspect of CRPS management, although numerous uncontrolled studies suggest the likely utility of several approaches. These approaches include various forms of relaxation training, biofeedback, mindfulness training, and cognitive and behaviorally focused interventions including graded exposure therapy. Successful implementation of these interventions requires recognition of the unique issues in CRPS patients, particularly the pervasive learned disuse often seen in such patients. Clinical experience using techniques like those described above in an integrated multidisciplinary context indicates that many CRPS patients can achieve significant improvements in functioning and ability to control pain.

## Interventional Therapies

Numerous interventional therapies have been described but usually poorly studied. As the mechanisms and pathophysiology of CRPS are multifactorial, this presents unique challenges to treatment due to the dynamic and varied/diverse nature of its clinical symptoms. This section will review the historical evidence for the use of various traditional therapies in the treatment of CRPS, including sympathetic nerve blocks (SNB), intravenous regional anesthetic techniques (IVRA), “other” blocks (including somatic blocks and spinal infusions), neurolytic sympathetic blockade, and implantable therapies (including neuromodulation and targeted drug delivery). Recent publications of randomized controlled trials and their supporting evidence for the interventional treatment of CRPS have come from the field of neuromodulation, and in particular, dorsal root ganglion stimulation. This is an advanced form of spinal cord stimulation used to treat focal neuropathic pain, and studies published in 2017 [351] and 2019 [352] allowed this therapy to emerge as important later stage considerations. Traditional spinal cord stimulation is an FDA-approved treatment for chronic pain, initially introduced to market in 1967 [353].

Included in this review will be several topical reviews and meta-analyses identified in a 2020 PubMed search that provide an update from the previous edition. The Cochrane Database for Systematic Review will be used to highlight quality studies, to explore newer and existing treatments, and to facilitate some aspects of clinical decision-making.

When patients are not making notable improvements in function with conservative exercise therapy, more invasive treatment may be considered to mitigate the status and progression of chronic CRPS. The Malibu algorithm is discussed above [58]. A traditional treatment strategy in certain clinics is to initiate regional nerve blocks in conjunction with structured exercise therapy early in the treatment. Progression to neuromodulation may be considered if the patient has significant limitations or functional deterioration during treatment, and spinal cord stimulation and dorsal root ganglion stimulation may prove to be effective and long-term strategies to in a sub-set of patients (level 2).

### Sympathetic Nerve Blocks

Sympathetic blockade with local anesthetics has long been a traditional part of the armamentarium of regional nerve blocks utilized to treat CRPS. Several decades ago, the prevailing opinion as proposed by Livingston was that the disorder causing the symptoms and physical exam findings of CRPS were due to an abnormal upregulation of the sympathetic nervous system [354] (although this is questionable [257]). Therefore, sympathetic nerve blocks were historically thought to be a necessary step in managing pain caused by CRPS and to facilitate progress in the interdisciplinary algorithm.

Multiple mechanisms have been postulated in the development of CRPS, including the involvement of nociceptor/peripheral and regional sensitization, central sensitization, the somatosensory, sympathetic, and motor systems [129]. Autonomic signs of CRPS include skin temperature and color asymmetry, local inflammation andedema, that contribute to pain out of proportion to the initial injury [354, 355]. Sympathetic nerve blocks have historically been considered an important procedure both in the diagnosis (i.e., Sympathetically Maintained Pain; SMP) and treatment of CRPS [356]. In the subgroup of CRPS patients with SMP (responsive to sympathetic blocks), there is some evidence suggesting the coupling of sympathetic nerves with several types of afferent nerve fiber types in the peripheral and central nervous system [357] completing feed-back and feed-forward loops in CRPS (level 4).

Sympathetic nerve blockade (SNB) is performed at the transverse process at the level of Chassaignac's tubercle (the sixth cervical vertebral body) for upper extremity CRPS, and for lower extremity CRPS it is performed at the second and third lumbar vertebral body. The pain relief following SNB generally outlasts the effects of the local anesthetic and may be long lasting in some cases [358] (level 2), [359] (level 4). In addition to these anatomic local anesthetic blocks, other sympatholytic procedures, including intravenous (IV) phentolamine; IV regional anesthetic blocks (Bier blocks) with either lidocaine, bretylium, clonidine, reserpine, or guanethidine; and epidural infusion have been described [217, 360–363].

Sympathetic nerve blocks lack high quality evidence to support a definitive role in the treatment of CRPS. Previously, it was felt that at least one SNB was necessary in order to classify CRPS as SMP or sympathetically independent pain (SIP) [364, 365] with the simple pragmatic goal of determining if sympathetic blocks should be part of the treatment regimen. This procedure is now usually performed with fluoroscopy; after performing these blocks there are often differences between clinical assessment (pain and function) and the observed clinical success of the SNB (vasomotor changes) secondary to varying degrees of sympatholysis [366]. Thus, the role of this block is largely empiric. Although currently out of favor, these blocks may be clinically important in a subset of SMP cases if these blocks mitigate pain, improve function, and provide a less painful “window of opportunity” for rehabilitation techniques.

A systematic review (level 1) by Cepeda et al. was published in 2002, which reviewed all available literature regarding local anesthetic sympathetic nerve blockade from 1916 through 1999 [367]. The general conclusion of this systematic review was that SNB was not effective. These older reports tend to be relatively imprecise and performed on heterogeneous/nonspecific cohorts [367].Although the techniques did not show a significant effect by this analysis of general diagnostic groups, it is important to note that a sub-set of patients may respond (level 4). Less than 20% of the articles reviewed by Cepeda critically evaluated the success of their blocks [366].

A significant confounding factor is a lack of consensus on defining “a successful sympathetic block.” There are several studies available to clarify relevant issues. Price et al. performed a comparative study of local anesthetic versus saline stellate ganglion or lumbar sympathetic blocks in seven CRPS patients in a double-blind crossover fashion [358]. Onset of analgesic effect occurred within 30 minutes in *both* groups, with the local anesthetic group (lidocaine/bupivacaine mixture) having a significantly greater duration of relief (mean of 3 d, 18 h vs 19 h) [358], thus showing at least short-term analgesic efficacy of local anesthetic sympathetic blockade for CRPS (level 2). Bonelli et al. performed a randomized trial of stellate ganglion block versus “active control” (in the form of guanethidine IV regional block) [56]. They found significant improvement in both groups, with no significant difference between the SGB and IVRA guanethidine groups (level 3), although this finding is difficult to interpret given the absence of a non-block control condition.

Raja et al. undertook a blinded prospective trial of IV phentolamine infusion versus local anesthetic sympathetic blockade in 20 patients (10 upper and 10 lower extremity SMP patients). They found a high correlation between analgesia with SNB and IV phentolamine infusion and concluded that either technique could distinguish between SMP and SIP (level 3) [368].

In a observational study (level 3) of 54 stellate ganglion blocks, Malmqvist et al. [369] defined a strict sympathetic block success criterion of development of: (1) Horner’s syndrome (2) Increase in skin temperature >34C (3) Increased skin blood flow >50% by laser Doppler flowmetry (4) abolished skin resistance response in ulnar distribution and (5) abolished skin resistance response radial; 4 of 5 defined “success.” Only 15 of 54 blocks included in this study met this strict criterion for a successful block [369], which perhaps indicates a relatively high rate of partial or incomplete sympathetic blockade in clinical practice. This upper limb CRPS study concluded that Horner’s syndrome was not always associated with an increase in skin temperature, initial low palmar skin temperature blood flow was associated with greater increase of the five defined parameters aforementioned above, injection towards the transverse process of C7 promoted a better block than toward C6, and high concentration of local anesthetic improved the success of the sympathetic block. Schurmann et al. showed the clinical difficulty regarding correlation of limb temperature elevation, Horner’s syndrome, and complete sympathetic block as measured by a complex experimental design in a large group of CRPS type I patients [366]. This study demonstrated that even in the case of significant limb temperature elevation, the sympatholysis may be incomplete, with the same holding true for the Horner’s syndrome. Additionally, even in patients with “complete sympatholysis,” the rate of analgesia obtained following the stellate ganglion block was little higher than 50%, clearly demonstrating subgroups of SIP and SMP within this group of 33 CRPS type I patients.

There is some evidence for the efficacy of the classic SGB and LSB in an apparent subset of subjects (level 3) as above. Apart from possible efficacy as an intervention, a secondary reason these blocks remain in most CRPS treatment algorithms is the clinical differentiation of SMP from SIP and, thus, to provide a rationale for a course of sympathetic blockade and perhaps (controversially) neuro-ablation in this subset of CRPS patients with SMP. The empirical utility of the SGB or LSB when used in a short series *in conjunction* with active reanimation physiotherapy is advocated based on consensus recommendations (level 4) [58]. Surprisingly there is very little good evidence for LSB therapy. Carroll et al performed a study (level 3) involving the combination of bupivacaine plus botulinum toxin versus bupivacaine alone in a trial including 9 patients undergoing LSB for CRPS. These authors found that addition of botulinum toxin prolonged the duration of analgesia from a mean of 10 days to 71 days [236].

### Other Blocks and Infusion Techniques

There are numerous case reports supporting the use of brachial plexus blockade in the CRPS literature (level 4). Indications for continuous brachial plexus infusion include peri-operative, post-trauma, post-operative pain relief, vascular compromise, intractable pain from CRPS I and II, and phantom limb pain (level 4). The brachial plexus is an ideal location for a continuous regional technique, because of its well-defined peri-vascular compartment and the close approximation of the large number of nerves supplying the upper extremity. Catheters have been kept in place in the same position for as long as three weeks (level 4) [370]. The brachial plexus catheter may be connected to a constant infusion of local anesthetic, opioid, clonidine, and other adjuvants (level 4). Sympatholysis can still be maintained for up to 2–3 weeks with 0.1 to 0.2% ropivacaine in a reliably anchored catheter (level 4) [371].

Wang et al. reported placement of an axillary catheter in a patient with severe CRPS II 30 days post carpal tunnel release (level 4) [372]. These authors started with a concentration of bupivacaine of 0.1% at 2.5 mL/hour and noted a dense motor and sensory block with excellent analgesia. Within one day, they decreased the infusion to 0.05% bupivacaine, stopped the basal infusion, and allowed a 1 mL patient-controlled dose every 15 minutes. The patient had continued analgesia with resolution of the motor block, allowing active physical therapy, and with the catheter left in place for 1 week. The complications of a continuous brachial plexus infusion are similar to those of a brachial plexus block plus the infectious risks of a long-term catheter. These complications include bleeding, infection, intravascular injection, intrathecal injection, pneumothorax, and phrenic nerve paralysis (level 4).

Epidural infusion is an alternative therapy to provide pain control, by allowing one to vary local anesthetic concentration and infusion dose to be titrated to the desired effect (level 4). Adjuvant medications, such as clonidine with the addition of opioids, can be added to provide additional spinal analgesia and to potentiate the degree of relief (level 4). The most commonly used combination of epidural medications today includes clonidine with bupivacaine. Opioids can be added to the mix if the pain relief is inadequate, or if the local anesthetic concentration required to produce pain relief also prohibits ambulation or full participation in the physiotherapy program (level 4). The primary benefit of continuous regional analgesia is that one is able to effectively titrate to the necessary degree of relief and promote active physical therapy as tolerated (level 4). Furthermore, with patient-activated bolus programming, these continuous regional techniques allow patients to self-administer small boluses for optimal analgesia as the pain levels fluctuate (level 4). Either before or after a strenuous exercise program, patients may experience elevations in pain, swelling, or allodynia. The ability for patients to readily self-administer extra doses of medication within certain pre-programmed parameters will improve patient satisfaction and optimize pain control (level 4). Rauck et al. performed a randomized, blinded, placebo-controlled trial (level 2) utilizing epidural clonidine [217]. They randomized 26 patients with CRPS to receive daily epidural infusions (for 3 consecutive days) of clonidine 300 or 600 mcg, or placebo. [217] If patients responded to the clonidine with analgesia (and did not respond to placebo), they were placed on an open label infusion for a mean of 32 days at a mean dose of 32 mcg/hour. All patients had substantial relief with both the 300 and 700 microgram doses. Of the 26 patients, 19 elected to receive continuous infusions of clonidine for an average of 43 days with an average dose 32 ± 6 micrograms per hour. Seventeen of nineteen patients had statistically significant improvement in pain. Side effects were dizziness, dry mouth, mouth sores, and nausea. Six of 19patients developed catheter related infection, and one developed meningitis [217].

Cooper et al. studied 14 patients in a prospective open label trial and demonstrated improved pain relief and range of motion in patients receiving an epidural bupivacaine-opioid mixture for an average of 4 days (level 3) [373]. Thirteen of fourteen patients had significant improvement, with 11 of the 14 achieving “resolution of their CRPS” (by the end of the trial) with no activity restrictions. Konig et al. studied 26 patients by using continuous cervical epidural analgesia of bupivacaine (0.25%) for seven days coupled with physical therapy (level 3) [374]. Eighty-three percent of patients had “improvement in pain.” Edema, sweating abnormalities, and dysfunction of the hand responded particularly well. Sixty-three percent of patients considered their condition to be acceptable whereas only 8% were completely pain free. Reduction in pain medications usage was also noted. Finally, Bucheit and Crews described a single case report where continuous epidural infusion markedly improved range of motion (level 4) [375].

The reported rates of infection in epidural catheters used to treat CRPS are as high as 31% [217]. Thus, epidural catheters meant for longer-term use should be performed as minor surgical procedures that require standard surgical sterility techniques. Catheters should be tunneled under the skin and away from the midline entrance point to the spine to minimize the colonization by bacteria that is inherently a greater risk with extended duration infusions. Standard catheter dressings, such as those required for extended central venous catheters, should be followed and dressings should be changed weekly (level 4). The hallmarks of an epidural abscess include the triad of back pain, sensorimotor loss, and loss of bowel and bladder function. Epidural abscesses may have earlier prodromal symptoms such as fever, neck pain, or photophobia [376]. Careful attention to early symptoms is paramount for early diagnosis. A previous study has demonstrated a catheter related infection rate of 19 out of 350 patients. All of these patients were treated with antibiotics and catheter removal, and none required surgical intervention [376].

Intrathecal analgesia has been studied to a lesser extent when compared to epidural analgesia. Lundborg reported a series of three patients with refractory CRPS, who did not have a favorable clinical response to intrathecal bupivacaine. In spite of initial analgesia, all patients demonstrated a progression of their CRPS (level 4) [377]. In a small subset of patients (n = 7) with refractory CRPS and severe dystonia, van Hilten et al. demonstrated analgesia and functional restoration after a bolus of intrathecal baclofen injected in a double-blind fashion followed by intrathecal infusion (level 3 evidence for IT baclofen in *dystonic* CRPS) [230].

Some have adopted epidural infusion techniques as next line therapy for patients failing intermittent blocks with some evidence for efficacy with epidural clonidine (level 4).The ease of this procedure, along with level 3 evidence supporting epidural clonidine infusion as outlined above, makes this a favorable next line therapy. Some centers have utilized the plexus infusions described above, but the epidural techniques are more common (level 4). The major risk associated with these infusion techniques is the rate of infection, which remains to be defined by further prospective study on infusion techniques in CRPS patients. Intrathecal baclofen infusion by implanted drug delivery systems was recommended in patients with a dystonic component to their CRPS (level 3), but van Hilten et al stopped using intrathecal baclofen infusions due to what they deemed as unacceptable side effects (personal communication). Intrathecal infusion for CRPS without dystonia has only limited supporting literature [230].

### Neurolytic Sympathetic Procedures

Surgical sympathectomy has been utilized to treat SMP and other hyperactive sympathetic syndromes (including hyperhidrosis and Raynaud’s phenomenon among others) since 1889. Historically, this was an important treatment for “RSD” [378, 379]. These surgical techniques were performed in an open operation, but recently both upper and lower extremity sympathectomy are being done via endoscopy with a minimally invasive technique, as initially described in the 1950s and recently “re-discovered” in a small prospective case series (level 3) [356]. More recently, radiofrequency techniques have been described in a large case series (level 3) [378].

Kim et al. reviewed the available literature for surgical sympathectomy (level 1) and found an initial failure rate of up to 35%, usually ascribed to poor patient selection [379]. Other possibilities for failure to achieve analgesia include incorrect diagnosis, inadequate resection, reinnervation, and contralateral innervation (level 4). In light of the difficulty of clinically assessing adequacy of sympathetic blockade based on clinical criterion, it is easy to understand the difficulty in assessing the local anesthetic sympathetic block’s predictive value for surgical sympathectomy [366]. The ablative sympathectomy techniques have been available for many years, but as yet, no high quality evidence exists to support their use and these techniques have fallen out of favor due primarily to an imbalance of efficacy versus significant adverse effects (level 4). Another significant problem with ablative sympathectomy is the recurrence of former symptoms and post sympathectomy neuralgia 6 months to two years post sympathectomy [380]. These post ablative neuralgic syndromes hypothetically may respond to re-resection or spinal cord stimulation but this has never been conclusively demonstrated. The reported incidence of post sympathectomy neuralgia is up to 44% in a series of open sympathectomy for causalgia [380].

Wilkinson reports the largest series of percutaneous Radio Frequency (RF) lesioning of the thoracic T-2 distribution sympathetic outflow (RF sympathectomy) with over 350 procedures performed for hyperhidrosis (not specifically for CRPS). Of these patients , 86% showed signs of sustained sympathectomy at a three-year follow-up, although there was no assessment of clinical analgesic or functional outcomes (level 3 evidence for *interruption of sympathetic activity* in a prolonged fashion with RF lesioning techniques) [381]. Wilkinson reported difficulty with lumbar percutaneous RF techniques due to variability of the lumbar anatomy versus the thoracic ganglion. He also reported a low rate of post-procedure neuralgic syndromes (around 5%); although, this was published in a non-peer-reviewed book chapter (level 4) [381]. There are no published randomized controlled data available on efficacy of pulsed RF sympathetic ganglion techniques for CRPS.

Sympathetic ablation techniques have been advocated for CRPS for many years, mainly by surgeons. In general, neurodestructive techniques to treat chronic pain syndromes are rarely recommended, because they may aggravate pain and cause deafferentation syndromes or post sympathectomy neuralgia [381]. The same holds true for neurolytic blocks utilizing alcohol or phenol, which have largely been relegated to the terminally ill [380]. The ability to control the size of the lesion with radiofrequency ablative techniques is better than neurolysis (level 4). Obviously, both techniques are less invasive than surgical ablation. The exact role of RF ablation sympathectomy or periodic blockade is uncertain because of a lack of evidence.

### Neurostimulation

The Melzack and Wall gate theory was first described in the literature in 1965, and this was the first mentioned hypothetical rationale for the mechanism of action of spinal cord stimulation and the central transmission of pain [83]. The dorsal horn of the spinal cord works to regulate transmission of signals from the periphery to the central nervous system and centers of the brain [83]. This theory was developed around the delivery of electrical energy from spinal cord stimulator electrodes to the spinal cord causing a preferential stimulation of large afferent fibers with concomitant blockade of smaller C and A-delta nerve fibers [83].

A PubMed search for CRPS and SCS from 2000 to 2021 revealed 118 published manuscripts and of those, eight were randomized controlled trials and three were meta-analyses. A summary of these publications can be found in Appendix A2. Results of key studies identified in this review are detailed in context below.

Failure to progress in an interdisciplinary model/functional restoration algorithm and more intensive non-invasive therapies may warrant consideration of treatment with spinal cord stimulation or dorsal root ganglion stimulation. Conventional SCS stimulation offers an opportunity to inhibit the nociceptive pathways at the level of the dorsal column of the spinal cord, while DRG stimulation modulates pain signal pathways at the level of the dorsal root [382]. Data demonstrates pain reduction, improved quality of life and function, as well as a reduction in opioid pharmaceuticals when spinal cord stimulation is employed in the setting of failed conservative therapy [383]. There are several studies that have shown spinal cord stimulation to be safe and effective for the treatment of chronic pain from CRPS [352].

Kemler et al. published the first prospective, randomized trial to compare spinal cord stimulation (SCS) placed in the dorsal epidural space to conservative therapy (physical therapy) for CRPS [384]. (level 2). In this study, 36 patients with “reflex sympathetic dystrophy” (type I CRPS; of duration 6 months or longer) were assigned to receive a “physical therapy program” (undefined, and variable; making the active control problematic) together with spinal cord stimulation, whereas 18 patients were assigned to receive PT alone. In 24 of the 36 patients randomized to SCS, the trial was deemed successful and permanent implantation was performed. At a 6-month follow-up assessment, the patients in the SCS group retained a reduction in pain, and a significant percentage graded the global perceived effect as “improved.” However, there were no clinically significant improvements in functional status. The authors concluded that in the short-term, SCS reduces pain and improves the quality of life for patients with CRPS involving the upper extremities. The improvements in pain ratings, global perceived effect, and overall health related quality of life, although modest, were significant and partially sustained for two years follow-up as published in a subsequent manuscript (level 2) [385]. Further analysis of this patient subgroup has revealed no difference in outcomes for cervical versus lumbar SCS in terms of effectiveness or complication rate [386].

Kriek et al. in 2018 investigated the effects of spinal cord stimulation on various immunomodulating cytokines, chemokines, and growth factors, including interleukin (IL)-2, IL-4, IL-5, IL-6, IL-10, IL-12, IL-13, IL-15, IL-17, tumor necrosis factor (TNF)-alpha, and interferon (IFN)-gamma. The design of this study was a multicentered, randomized control trial that examined the effects of SCS of various waveforms in patients with CRPS (level 2) [387]. Thirteen patients were included in the analysis of skin blister fluid. Those who consented to this study required a baseline skin blister fluid test before proceeding to a two week trial stimulation period with a 40 Hz standard tonic SCS. If the trial was successful then those patients received an implantable pulse generator and received 40 Hz frequency stimulation for the next 3 months. At follow up, these patients received successive skin blister fluid testing which investigated the effects of various frequencies and waveforms, including standard 40 Hz, 500 Hz, 1200 Hz, burst, and placebo stimulation. At the end of the crossover period, patients preferred stimulation settings were chosen and continued for another 3 months, where the final skin blister fluid test was performed. This study suggested a systemic attenuation of T-cell activity, and IP-10 chemokine over time. Reduction in vascular endothelial growth factor and platelet derived growth factor were decreased after SCS, most probably due to increased peripheral tissue oxygenation.

In 2010, Van Eijs et al. published a randomized control trial (level 2) in which 36 CRPS patients were selected based on the diagnosis of CRPS type 1 [388]. Twenty-four of those patients responded to SCS and proceeded to implantation of a permanent device. Using the Semmes-Weinstein psycho-physical test brush evoked allodynia was assessed by transiently stroking the skin of the subject’s hands and feet at nine sites. If this procedure was “painful” these patients were noted as having brush allodynia. After 1 year, 20 out of 24 (83%) of the SCS implanted patients maintained significant pain reduction. The results also demonstrated that the presence of brush evoked allodynia may be a negative predictor for successful SCS treatment.

Dorsal root ganglion (DRG) therapy was brought to market in the United States in 2016.The “ACCURATE” study was the first randomized, controlled multicenter trial of a device that compared DRG stimulation to conventional SCS in the setting of chronic intractable pain of the lower limbs attributed to CRPS (level 2) [351]. Patients with a six-month history of chronic intractable pain of the lower limbs associated with CRPS type 1 or 2 diagnosed by the Budapest Criteria were randomized to either DRG stimulation or conventional SCS in a 1:1 ratio. The temporary trial phase ranged anywhere from 3 days to 30 days. The average trial duration for DRG stimulation was 5.8 days (SD 2.8 days), and was also 5.8 days for the SCS group (SD 5.1 days). Patients with a successful trial, which was defined by at least a 50% reduction in VAS for lower limb pain relief and freedom from any new neurological complaints, were implanted with a permanent device. These patients were followed for 12 months, and the results of the primary endpoint revealed that subjects using DRG stimulation had a higher rate of treatment “success” (81.2%) when compared to conventional SCS (56.7%). While pain relief was noted to be greater with the DRG group the authors also hypothetically concluded that patients with DRG therapy also may have had “improved quality of life and psychological disposition.”

In 2020, Mekhail et al. published a subgroup analysis from the previously published ACCURATE study [351, 389]. A retrospective analysis was conducted with 61 patients, who received a DRG neurostimulator implant. The outcomes of patients with paresthesia-free stimulation was compared to those who experienced paresthesia, measured at 1, 3, 6, 9, and 12 month follow ups. The percentage of patients with paresthesia free pain relief increased from 16.4% at 1 month to 38.3% at 12 months (level 3). Authors concluded that paresthesia based neurostimulation is not required for pain relief and is not congruent with trial success.

Randomized controlled studies of SCS for CRPS demonstrate “weak recommendations” as stated by Dworkin et al. [390]. The benefit of “shared decision making” should be emphasized with the patient by explicitly sharing the risks, benefits, and alternatives to this therapy. Dworkin et al, suggests reserving SCS therapy for patients who did not respond adequately to non-invasive treatments and sympathetic nerve blocks or for patients where nerve blocks are not appropriate [390]. In 2020, Huygen et al. published a meta-analysis (level 1), which identified 217 patients with a permanent DRG implant at 12 months follow-up [391]. Using a 10 point Numeric Rating Scale (NRS) the analysis of pooled data overall showed a weighted mean pain score of 3.4, with 63% of patients demonstrating ≥ 50% pain relief. This study included efficacy sub-type analyses for CRPS type 1, causalgia (type II) and low back pain resulting in a mean NRS reduction in pain intensity of 4.9, 4.6, and 3.9, respectively. Thus, there is evidence supporting SCS and DRG for the treatment of CRPS, but high-quality and corroboratory evidence is needed.

### IV Regional Anesthetic Blocks (IVRA)

Intravenous regional anesthesia involves the infusion of pharmacological agents to the tourniqueted limb affected by CRPS [392]. Numerous IVRA medications, alone and in combination, have been reported to have efficacy in treating CRPS. IVRA with guanethidine, lidocaine, bretylium, clonidine, droperidol, ketanserin, or reserpine have been described and reviewed critically by Perez et al. [51], Forouzanfar et al. [145], and Kingery [227].

Perez et al. undertook a meta-analysis (level 1) of the highest quality trials (blinded, with re-evaluation of included trials, statistical methodology; and utilizing trials meeting strict inclusion criteria such as randomization, blinding, sample size, dropout rate), finding 11 acceptable trials of “sympathetic suppressors,” with 9 being IVRA studies and 6 concerning guanethidine in particular [227]. Perez et al. applied a quantitative analysis of effect size, which compares the difference in pain relief between experimental and control groups, with a correction factor applied for trial size. This method has become acceptable in meta-analysis to analyze aggregate treatment effect from numerous studies. Their aggregate analysis showed a *lack of proven effect* of IVRA, and, more specifically, a lack of proven effect of guanethidine IVRA (thus level 1 evidence for *lack* of proven effect of these therapies), although subgroups may have responded well.

Several quality studies have also reported a negative outcome of the IVRA intervention (no better than placebo). Ramamurthy et al. performed a double blind, crossover, controlled outcome study with 60 CRPS I patients randomized to receive IVRA blocks every four days for a total of four blocks with either guanethidine (one, two, or four guanethidine blocks) or a placebo with 0.5% lidocaine. After the first block, placebo response was higher than guanethidine, and six months after the last block (up to four), 35% of patients had significant pain relief with no difference between placebo and guanethidine arms (level 2 evidence for *lack* of effect of guanethidine over placebo) [393]. Confounding factors in this study include the fact that the “placebo” group received an IVRA using local anesthetic (0.5% lidocaine) and a tourniquet (which may itself confer some type of analgesic effect following the block); thus in reality, the “placebo” control may be considered an active treatment comparison group.

Jadad et al. used an enriched trial design and prospectively enrolled patients who reported pain relief with open label guanethidine IVRA, to a double-blind treatment phase with crossover design. No differences between guanethidine and placebo were seen, and this study was terminated early for side effects (level 2 evidence for *lack* of effect) [394]. Blanchard et al. compared the effects of IVRA with guanethidine versus reserpine versus saline. This was a crossover design, changing to another agent if inadequate analgesia occurred with a block (level 3). Only 21 patients were studied, but no differences between treatment types were discernable at short-term follow-up [363]. The placebo saline infusion was done with a tourniquet in similar fashion to the active drug block; thus, this does not control for a tourniquet induced effect on the extremity (e.g., tourniquet-induced analgesia, compression-induced alteration of local cytokines), leading to methodological problems with the “control” group for most IVRA studies as above [227]. Rocco et al. did a small randomized, double-blind, active controlled trial of reserpine and guanethidine (at different times) versus lidocaine alone in IVRA [395] (level 3). They noted significant relief following the block with no difference between the reserpine, guanethidine, or “control” (lidocaine) group.

The notable exception to these negative trials was Hord et al., who found a positive response with bretylium in a prospective randomized double-blind fashion versus lidocaine (level 2) [361]. Bonelli et al. as above compared IVRA guanethidine to SGB in a cohort of 19 “RSD” patients, [66] and demonstrated “comparable efficacy.” Overall, the evidence supporting efficacy of IVRA is of low quality. The use of bretylium, phentolamine, clonidine, lidocaine, and ketorolac, alone and in combination with the IVRA modality all lack high quality evidence to support efficacy. As our understanding of the peripheral alterations in cytokines in CRPS are clarified, the IVRA technique may eventually define targeted pharmacotherapy using this technique [396]. Bretylium is unavailable in the USA.

### Other IV Infusions

An infusion of phentolamine, a short acting alpha-adrenergic blocking agent, has been postulated as a test for SMP [360]. Arner reported a critical analysis of the use of phentolamine infusion followed by IVRA guanethidine to assess the clinical response to the phentolamine infusion and assess the positive predictive value of the phentolamine infusion on success of a subsequent IVRA guanethidine block [360]. Arner reported the results by patient subgroups, specifically, adults with causalgia and RSD versus children with causalgia and RSD. In adults, Arner found that approximately 50% obtained positive analgesia with IVRA phentolamine infusion and a very strong correlation to a good response to guanethidine. In children, 37 of the 47 obtained markedly positive analgesia to phentolamine infusion and a strong correlation to an excellent response to IVRA guanethidine (32/37 excellent response). Arner concluded that phentolamine caused no complications and provided “diagnostic” information as to the presence of SMP and prognostic information about subsequent response to guanethidine (level 3 evidence for IV phentolamine) [360]. A major weakness of the Arner study was the lack of a control or placebo group. By contrast, Verdugo et al. found that neither placebo, phentolamine, nor phenylephrine infusions resulted in any significant changes in pain, QST testing, regional blood flow, or hyperalgesia, and that there was no difference between groups in a prospective, single blinded, non-randomized study (level 3 evidence for *lack* of effect of phentolamine) [397].

A critical evaluation of IV infusion of lidocaine was undertaken by Wallace et al. in a randomized, double-blind trial [398]. They studied 16 patients with CRPS I or II with three different levels of lidocaine infusion (1, 2, and 3 mcg**/**mL and placebo infusion), during which the patients underwent spontaneous and evoked pain assessment and detailed quantitative psychophysical testing. During the lidocaine (but not placebo) infusion, the patients showed evidence of a decrease in pain response to cold stimuli, a decreased response to cold or touch allodynia in previously allodynic areas, and a decrease in spontaneous pain (but only at the highest serum infusion level). Thus, the predominant effect was decreased pain in response to cool stimuli more so than with mechanical or spontaneous pain. There was no effect on pain induced by punctate stimuli (level 2 evidence for short-term decrease in pain response to IV lidocaine infusion).

In summary, as suggested by the work of Arner, IV phentolamine infusion has been used largely as a diagnostic tool to differentiate SIP from SMP [360]. IV phentolamine and IV lidocaine techniques have fallen out of favor in clinical practice.

### Motor Cortex Stimulation

Of all therapies, ranging from minimally invasive to interventional, motor cortex stimulation (MCS) is the most invasive form of treatment for pain conditions, including CRPS. The mechanism of action for MCS is the modulation of pathologic hyperactivity in the thalamic relay nuclei [399]. Deafferentation results in the loss of inhibitory control of the nociceptive neurons, and MCS has been shown to normalize this disinhibition to a greater degree than somatosensory cortex stimulation (level 4). Similar to SCS procedures, a trial is required prior to the implantation of MCS. Risks are surprisingly rare but complications may occur consistent with other chronically implanted hardware in functional neurosurgery (level 4).

**Figure 4.**
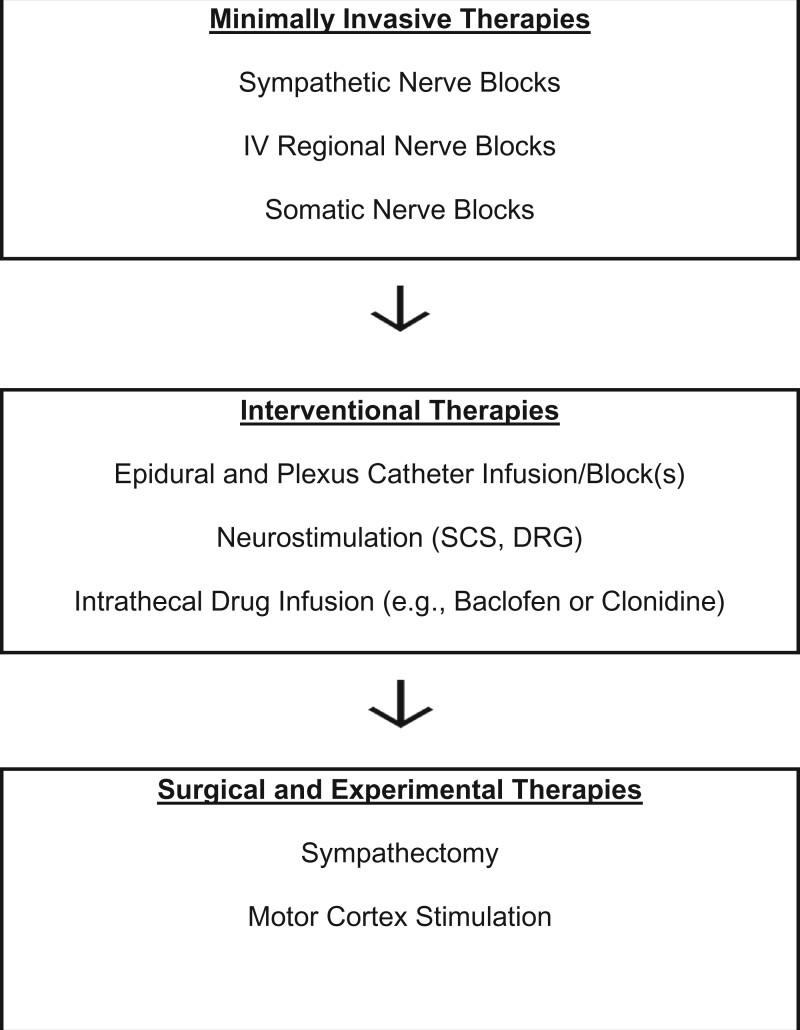
Consensus based, empiric Interventional Pain Treatment Algorithm for CRPS (modified from [58]).

According to the spirit of the Malibu treatment scheme (Figure 2) [58] simpler, less invasive, less dangerous and less expensive interventional techniques should be tried before invasive, experimental and much more expensive techniques (Figure 4 and Appendix A2).

Inadequate or partial response to any mentioned therapy may lead to a stepwise progression down through modalities always in conjunction with other non-interventional treatments.

In this 5th edition of these diagnostic and treatment guidelines, we find progress evident in diagnosis, clinical outcome measures, and evidence-based treatments compared to prior versions. Nonetheless, the conclusion in prior guidelines that “we need more high quality research regarding CRPS interventions” still stands. There are few interventions with efficacy that has been convincingly demonstrated, and until such data are available, reliance on the standard clinical principles of interdisciplinary pain and symptom management will be necessary. While evidence for efficacy of most CRPS interventions remains weak in CRPS patients as a group, we suggest that going forward there may be value in exploring intervention efficacy within empirically-identified CRPS subtypes (e.g., warm vs cold CRPS) to determine whether a precision medicine approach to CRPS management could enhance outcomes. In each clinical situation the specific risk, benefit and expense of any intervention must be carefully and continuously considered.

## Notes

In Memoriam: Roberto S.G.M. Perez, RPT, PhD, Department of Anesthesiology, VU University Medical Center, Amsterdam, the Netherlands.

Funding sources: Publication support sponsored by the Reflex Sympathetic Dystrophy Syndrome Association (RSDSA).

Disclosure and conflicts of interests: R. Norman Harden: Consultant for Takeda, Neumentum, BDSI/Colleagium and NeuroBo pharmaceuticals; Candida S. McCabe: none to report; Andreas Goebel: Produces medico-legal reports for the Court, and has consulted and has received consultancy fees from Novartis on behalf of Liverpool University; Michael Massey: Medical Director for Tampa Bay Orthopedic Surgery Group and Independent Medical Review Expert for Maximus. No financial disclosures; Tolga Suvar: Consultant for Medtronic, Averitas Pharmaceuticals; Sharon Grieve: none to report; Stephen Bruehl: Paid consultant for NeuroBo pharmaceuticals. All authors received a stipend to write these semi-systematic reviews from the RSDSA.

Supplement sponsorship: This article is for a supplement entitled “Complex Regional Pain Syndrome: Practical Diagnostic and Treatment Guidelines, 5th Edition” sponsored by the Reflex Sympathetic Dystrophy Syndrome Association (RSDSA).

## Appendix

**Table A1.** Studies examining psychological/behavioral interventions for CRPS

Author	Design and Sample	Psychological Intervention	Outcome
Blanchard 1979 [308]	Case Report	Thermal biofeedback	Complete resolution of symptoms
	n = 1 adult		
Alioto 1981 [309]	Case Report	Autogenic and breathing relaxation, thermal and muscular biofeedback	75–100% reduction in pain
	n = 2 adult/adolescent		
Barowsky et al. 1987 [310]	Case Report	Thermal biofeedback	Complete resolution of symptoms
	n = 1 child		
Kawano et al. 1989 [311]	Case Report	Autogenic relaxation, imagery	Complete resolution of symptoms
	n = 1 adolescent		
Wesdock et al. 1991 [312]	Case Series	Biofeedback	Helpful in some cases, particularly in CRPS of shorter duration
	n = 36 child/adolescent		
Gainer 1992 [313]	Case report	Hypnotic imagery, relaxation training	Complete resolution of symptoms
	n = 3 adult		
Wilder et al. 1992 [314]	Case Series	Multidisciplinary treatment including relaxation training and CBT	Significantly improved pain and function in 57% of patients
	n = 70 child/adolescent		
Fialka et al. 1996 [315]	Randomized Trial	PT (n = 9), PT+ autogenics (n = 9)	Pain improved in both groups equally. Skin temperature more improved in autogenics group.
	n = 18 adult		
Sherry et al. 1999 [103]	Case series	Multidisciplinary treatment including psychotherapy for 77% of sample	Complete symptom resolution in 92% of sample at end of treatment, 88% symptom-free at 2 year follow-up
	n = 103 child/adolescent		
Oerlemans et al. 1999, 2000 [64, 316]*	Randomized Trial	PT including relaxation training and cognitive interventions (n = 44), OT (n = 44), Social Work Control (n = 47). All patients received standard medical care.	Significantly greater improvements at 1 year follow-up for PT group than Controls on pain, temperature, active range of motion, and overall impairment scores
	n = 135 adult		
Lee et al. 2002 [72]	Randomized Trial	PT 1 X week + CBT (n = 14), PT 3	Pain and function improved significantly pre-post for both groups. Recurrence rate = 50%.
	n = 28 child/adolescent	X week + CBT (n = 14)	
Singh et al. 2004 [317]	Prospective Case Series n = 12 adult	4-week outpatient interdisciplinary treatment program including group psychotherapy	Function improved significantly pre-post treatment without corresponding increases in anxiety
de Jong et al. 2005 [104]	Series of prospective single-subject experiments n = 8 adult	Intensive graded exposure therapy targeting pain-related fear	Pain-related fear was significantly reduced, with corresponding decreases in pain intensity, disability, and other CRPS symptoms
Bartur et al. 2014 [318]	Quasi-experimental n = 20 adult	Paced slow breathing in CRPS patients (n = 10) and healthy controls (n = 10)	Paced breathing enhanced vagal tone as indexed by heart rate variability indexes in healthy controls, but not in CRPS patients.
Barnhoorn et al. 2015 [319]	Randomized trial	Pain exposure therapy (n = 28)	Pain exposure therapy produced significantly greater improvements in pain, disability, and active range of motion.
	n = 56 adult	Pain-contingent therapy (n = 28)	
Den Hollander et al. 2016 [320]	Randomized trial	Pain exposure therapy (n = 18)	Pain exposure therapy produced significantly greater improvements in pain and disability
	n = 35 adult	Pain-contingent therapy (n = 17)	
Solcà et al. 2018 [321]	Crossover trial	Virtual reality (VR) image of the affected limb flashing visually either in synchrony or asynchrony with the heartbeat	Synchronous VR resulted in significantly reduced pain, and improved motor function and vagal tone (heart rate variability) in CRPS patients but not controls.
	n = 48 adult	CRPS (n = 24)	
		Healthy controls (n = 24)	

Studies are listed in order of date of publication. CBT = Cognitive-Behavioral Therapy, PT = Physical Therapy, OT = Occupational Therapy.

* Both Oerlemans et al. studies were based on the same sample.

**Table A2.** Studies examining SCS intervention for CRPS

Author	Design and Sample	Outcome
Kumar et al. 1997 [406]	Non-randomized level 3	All 12 patients experienced pain relief by McGill Pain Questionnaire and Visual analog scale, eight patients exhibited excellent pain relief, four demonstrated good relief
	n = 12	
Bennett et al. 1999 [404]	Retrospective level 3	70% satisfaction for 1 lead, 91% satisfaction for 2 leads
	n = 101	
Kemler et al. 1999 [405]	Retrospective level 3	Of the 23 patients, 13 patients (57%) remained successfully treated with SCS
	n = 23	
Kemler et al. 2000 [384]	RCT level 2	Improved pain and quality of life, successful in 56% of patients in 6 months
	n = 36	
Kemler et al. 2004 [385]	RCT level 2	Successful in 63% of patients, 2-year follow-up of Kemler et al. 2000
	n = 36	
Kemler et al. 2008 [401]	RCT level 2	Patient satisfaction test at the 5-year mark showed patient satisfaction, but no difference between SCS and active placebo
	n = 20	
van Eijs et al. 2010 [388]	RCT level 2	SCS has an 81% chance of reducing pain for greater than 1 year if allodynia is absent before stimulation is started
	n = 36	
Deer et al. 2017 [351]	RCT level 2	The percentage of subjects receiving >50% pain relief and treatment success was greater in the DRG arm (81.2%) than in the SCS arm (55.7%) at 3 months
	n = 152	
Kriek et al. 2017 [402]	RCT level 2	Pain reduction and patient satisfaction was achieved with both standard and non-standard frequencies of SCS, although more patients favored non-standard
	n = 40	
Kriek et al. 2018 [387]	RCT level 2	After SCS both pro- and anti- inflammatory cytokines were reduced from the interstitial fluid blisters from the skin
	n = 17	
Deer et al. 2019 [403]	RCT level 2	DRG allows for focal stimulation compared to SCS with less paresthesia in the DRG group, 3.1% vs 9.1%
	n = 61	
Mekhail et al. 2020 [389]	Retrospective level 3	16.4% of patients with paresthesia free at 1 month increased to 38.3% at 12 months
	n = 61	
Taylor RS 2006 [400]	Meta-analysis level 1	A total of 25 studies were identified for CRPS, finding 67% of implanted patients with CRPS type 1 or 2 achieved pain relief of 50% or more with SCS
Dworkin et al. 2013 [390]	Meta-analysis level 1	Due to lack of high quality RCTs, weak recommendations can be made for (1) epidural injections for herpes zoster, (2) steroid injections for radiculopathy, (3) SCS for FBSS, (4) SCS for CRPS type 1
Huygen et al. 2020 [391]	Meta-analysis level 1	7 studies were reviewed, DRG demonstrated a reduction in pain for CRPS type 1, causalgia, and back pain with a mean reduction in pain intensity 4.9, 4.6, and 3.9 respectively

Studies are listed in order of date of publication. CRPS = Complex Regional Pain Syndrome, SCS = Spinal Cord Stimulation, RCT = Randomized Control Trial, DRG = dorsal root ganglion therapy, VAS = visual analog scale.

## References

[1] Stanton-Hicks M , Baron R, Boas R, et al Complex regional pain syndromes: Guidelines for therapy. Clin J Pain 1998;14(2):155–66. 9647459 10.1097/00002508-199806000-00012

[2] Perez RS , Zollinger PE, Dijkstra PU, et al; CRPS I Task Force. Evidence based guidelines for complex regional pain syndrome type 1. BMC Neurol 2010;10:20. 20356382 10.1186/1471-2377-10-20 PMC2861029

[3] Harden RN , Oaklander AL, Burton AW et al; Reflex Sympathetic Dystrophy Syndrome Association. Complex regional pain syndrome: Practical diagnostic and treatment guidelines, 4th edition. Pain Med 2013;14(2):180–229. 23331950 10.1111/pme.12033

[4] Stanton-Hicks M , Jänig W, Hassenbusch S, Haddox JD, Boas R, Wilson P. Reflex sympathetic dystrophy: Changing concepts and taxonomy. Pain 1995;63(1):127–33. 8577483 10.1016/0304-3959(95)00110-E

[5] Janig W , Stanton-Hicks M. Reflex Sympathetic Dystrophy: A Reappraisal. Seattle: IASP Press; 1996.

[6] Merskey H , Bogduk N. Classification of Chronic Pain: Descriptions of Chronic Pain Syndromes and Definitions of Pain Terms, 2nd edition. Seattle: IASP Press; 1994.

[7] Merikangas KR , Frances A. Development of diagnostic criteria for headache syndromes: Lessons from psychiatry. Cephalalgia 1993;13(Suppl 12):34–8. 8500145 10.1177/0333102493013S1208

[8] Harden RN , Bruehl S, Perez RS, et al Validation of proposed diagnostic criteria (the “Budapest Criteria”) for Complex Regional Pain Syndrome. Pain 2010;150(2):268–74. 20493633 10.1016/j.pain.2010.04.030 PMC2914601

[9] Harden RN , Bruehl S, Galer BS. Complex regional pain syndrome: Are the IASP diagnostic criteria valid and sufficiently comprehensive? Pain 1999;83:211–9. 10534592 10.1016/s0304-3959(99)00104-9

[10] Bruehl S , Harden RN, Galer BS, et al External validation of IASP diagnostic criteria for Complex Regional Pain Syndrome and proposed research diagnostic criteria. International Association for the Study of Pain. Pain 1999;81(1):147–54. 10353502 10.1016/s0304-3959(99)00011-1

[11] Diehr P , Diehr G, Koepsell T, et al Cluster analysis to determine headache types. J Chron Dis 1982;35(8):623–33. 7096526 10.1016/0021-9681(82)90014-5

[12] Drummond PD , Lance JW. Clinical diagnosis and computer analysis of headache syndromes. J Nerol Nerosurg Psychiatr 1984;47(2):128–33. 10.1136/jnnp.47.2.128 PMC1027680 6707652

[13] Bruehl S , Lofland KR, Semenchuk EM, Rokicki LA, Penzien DB. Use of cluster analysis to validate IHS diagnostic criteria for migraine and tension-type headache. Headache 1999;39(3):181–9. 15613212 10.1046/j.1526-4610.1999.3903181.x

[14] Maes M , Maes L, Schotte C, et al A clinical and biological validation of the DSM-III melancholia diagnosis in men: Results of pattern recognition methods. J Psychat Res 1992;26(3):183–96. 10.1016/0022-3956(92)90022-g 1432845

[15] Bruehl S , Ohrbach R, Sharma S, et al Approaches to Demonstrating the Reliability and Validity of Core Diagnostic Criteria for Chronic Pain. J Pain 2016;17(Suppl 9):T118–T131. 27586829 10.1016/j.jpain.2015.10.014

[16] Wilson PR , Low PA, Bedder MD, Covington EC, Rauck RL. Diagnostic algorithm for complex regional pain syndromes. In: Janig W, Stanton-Hicks M, eds. Progress in Pain Research and Management. Seattle: IASP Press; 1996: 93–105.

[17] Galer BS , Butler S, Jensen MP. Case report and hypothesis: A neglect-like syndrome may be responsible for the motor disturbance in Reflex Sympathetic Dystrophy (complex Regional Pain Syndrome-1). J Pain Sym Manage 1995;10(5):385–91. 10.1016/0885-3924(95)00061-3 7673771

[18] Schwartzman RJ , Kerrigan J. The movement disorder of reflex sympathetic dystrophy. Neurology 1990;40(1):57–61. 2296383 10.1212/wnl.40.1.57

[19] Kozin F , Ryan LM, Carerra GF, Soin JS, Wortmann RL. The reflex sympathetic dystrophy syndrome III: Scintigraphic studies, further evidence for the therapeutic efficacy of systemic corticosteroids, and proposed diagnostic criteria. Am J Med 1981;70(1):23–30. 6109448 10.1016/0002-9343(81)90407-1

[20] Gibbons JJ , Wilson PR. RSD Score: Criteria for the diagnosis of reflex sympathetic dystrophy and causalgia. Clin J Pain 1992;8(3):260–3. 1421741

[21] Galer BS , Bruehl S, Harden RN. IASP diagnostic criteria for complex regional pain syndrome: A preliminary empirical validation study. Clin J Pain 1998;14(1):48–54. 9535313 10.1097/00002508-199803000-00007

[22] Goebel A , Birklein F, Brunner F, et al The Valencia consensus-based adaptation of the IASP CRPS diagnostic criteria. Pain 2021;162(9):2346–8. 33729210 10.1097/j.pain.0000000000002245 PMC8374712

[23] Harden RN , Bruehl S, Stanton-Hicks M, Wilson PR. Proposed new diagnostic criteria for complex regional pain syndrome. Pain Med 2007;8(4):326–31. 17610454 10.1111/j.1526-4637.2006.00169.x

[24] De Takats G. Reflex dystrophy of the extremities. Arch Surg 1937;34:939.

[25] Schwartzman RJ , McLellan TL. Reflex sympathetic dystrophy: A review. Arch Neruol 1987;44(5):555–61. 10.1001/archneur.1987.00520170081028 3495254

[26] Bruehl S , Harden RN, Galer BS, Saltz S, Backonja M, Stanton-Hicks M. Complex regional pain syndrome: Are there distinct subtypes and sequential stages of the syndrome? Pain 2002;95(1-2):119–24. 11790474 10.1016/s0304-3959(01)00387-6

[27] de Mos M , Huygen FJ, van der Hoeven-Borgman M, Dieleman JP, Ch Stricker BH, Sturkenboom MC. Outcome of the complex regional pain syndrome. Clin J Pain 2009;25(7):590–7. 19692800 10.1097/AJP.0b013e3181a11623

[28] Veldman PH , Reynen HM, Arntz IE, Goris RJ. Signs and symptoms of reflex sympathetic dystrophy: Prospective study of 829 patients. Lancet 1993;342(8878):1012–6. 8105263 10.1016/0140-6736(93)92877-v

[29] Bickerstaff DR , Kanis JA. Algodystrophy: An under-recognized complication of minor trauma. Br J Rheumatol 1994;33(3):240–8. 8156286 10.1093/rheumatology/33.3.240

[30] Zyluk A. The natural history of post-traumatic reflex sympathetic dystrophy. J Hand Surg [Br] 1998;23(1):20–3. 10.1016/s0266-7681(98)80211-8 9571473

[31] Bruehl S , Maihöfner C, Stanton-Hicks M, et al Complex regional pain syndrome: Evidence for warm and cold subtypes in a large prospective clinical sample. Pain 2016;157(8):1674–81. 27023422 10.1097/j.pain.0000000000000569

[32] Dimova V , Herrnberger MS, Escolano-Lozano F, et al Clinical phenotypes and classification algorithm for complex regional pain syndrome. Neurology 2020;94(4):e357–‐e367. 31874923 10.1212/WNL.0000000000008736

[33] Harden RN , Bruehl S, Perez RSGM, et al Development of a severity score for CRPS. Pain 2010;151(3):870–6. 20965657 10.1016/j.pain.2010.09.031

[34] Harden RN , Maihofner C, Abousaad E, et al A prospective, multisite, international validation of the Complex Regional Pain Syndrome Severity Score. Pain 2017;158(8):1430–6. 28715350 10.1097/j.pain.0000000000000927

[35] de Mos M , de Bruijn AGJ, Huygen FJPM, Dieleman JP, Stricker BHC, Sturkenboom MCJM. The incidence of complex regional pain syndrome: A population-based study. Pain 2007;129(1-2):12–20. 17084977 10.1016/j.pain.2006.09.008

[36] Grieve S , Manns S, Glanville V, Llewellyn A, McCabe C. A survey of questionnaire outcome measures currently used in Complex Regional Pain Syndrome clinical trials. Br J Pain 11(2, S1).

[37] National Institute for Health (NIH) NIH Clinical Research Trials and You. 2020. Available at: https://www.nih.gov/health-information/nih-clinical-research-trials-you/list-registries. Accessed January 13, 2020.

[38] Psoter KJ , Rosenfeld M. Opportunities and pitfalls of registry data for clinical research. Paediatr Respir Rev 2013;14(3):141–5. 23707656 10.1016/j.prrv.2013.04.004

[39] Williamson PR , Altman DG, Blazeby JM, et al Developing core outcome sets for clinical trials: Issues to consider. Trials 2012;13:132. 22867278 10.1186/1745-6215-13-132 PMC3472231

[40] Grieve S , Perez RSGM, Birklein F, et al Recommendations for a first Core Outcome Measurement set for complex regional PAin syndrome Clinical sTudies (COMPACT). Pain 2017;158(6):1083–90. 28178071 10.1097/j.pain.0000000000000866 PMC5438049

[41] Brunner F , Heitz C, Kissling R, et al German translation and external validation of the Radboud Skills Questionnaire in patients suffering from Complex Regional Pain Syndrome 1. BMC Musculoskelet Disord 2010;11:107. 20515455 10.1186/1471-2474-11-107 PMC2893083

[42] Flor H , Fydrich T, Turk DC. Efficacy of multidisciplinary pain treatment centers: A meta-analytic review. Pain 1992;49(2):221–30. 1535122 10.1016/0304-3959(92)90145-2

[43] Guzmán J , Esmail R, Karjalainen K, Malmivaara A, Irvin E, Bombardier C. Multidisciplinary rehabilitation for chronic low back pain: Systematic review. BMJ 2001;322(7301):1511–6. 11420271 10.1136/bmj.322.7301.1511 PMC33389

[44] Harden RN , Swan M, Costa BR BJ, King AL, Interdisciplinary Management. In, Harden RN. ed. *Complex Regional Pain Syndrome: Treatment Guidelines*, 3rd edition. Milford, CT: RSDSA Press; 2006:12–24.

[45] Carlson LK , Watson HK. Treatment of reflex sympathetic dystrophy using the stress-loading program. J Hand Ther 1988;1(4):149–54.

[46] Watson HK , Carlson L. Treatment of reflex sympathetic dystrophy of the hand with an active “stress loading” program. J Hand Surg [Am] 1987;12(5):779–85. 10.1016/s0363-5023(87)80069-2 3655243

[47] Graham A , Ryan CG, MacSween A, et al Sensory discrimination training for adults with chronic musculoskeletal pain: A systematic review. Physiother Theor Pract 2020;1–19. 10.1080/09593985.2020.1830455 33078667

[48] Swan M. Treating CRPS: A Guide for Therapy. Milford, CT: RSDSA Press; 2004.

[49] Crombez G , Vlaeyen J, Heuts P, Lysens R. Pain-related fear is more disabling than pain itself: Evidence on the role of pain-related fear in chronic back pain disability. Pain 1999;80(1-2):329–39. 10204746 10.1016/s0304-3959(98)00229-2

[50] Crombez G , Vervaet L, Lysens R, Baeyens F, Eelen P. Avoidance and confrontation of painful, back-straining movements in chronic back pain patients. Behav Modif 1998;22(1):62–77. 9567737 10.1177/01454455980221004

[51] Kingery WS. A critical review of controlled clinical trials for peripheral neuropathic and pain complex regional pain syndromes. Pain 1997;73(2):123–39. 9415498 10.1016/S0304-3959(97)00049-3

[52] Harden RN. The rationale for integrated functional restoration. In: Wilson PR, Stanton-Hicks M, Harden RN, eds. *CRPS: Current Diagnosis and Therapy*, Vol. 32. Seattle: IASP Press; 2005.

[53] Bruehl S , Steger H, Harden R. Assessment of complex regional pain syndrome. In: Turk D, Melzack R, eds. *Handbook of Pain Assessment*. New York: The Guilford Press; 2001.

[54] Fordyce WE , Fowler RS, Lehmann JF, et al Operant conditioning in the treatment of chronic pain. Arch Phys Med Rehabil 1973;54(9):399–408. 4729785

[55] Turk D , Melzack R. The measurement of pain and the assessment of people experiencing pain. In: Turk D, Melzack R, eds. *Handbook of Pain Assessment*. New York: Guilford Press; 2001.

[56] Bradley L , McKendree-Smith N. Assessment of psychological status using interviews and self-report instruments. In: Turk D, Melzack R, eds. *Handbook of Pain Assessment*. New York: Guilford Press; 2001.

[57] Davidoff G , Morey K, Amann M, Stamps J. Pain measurement in reflex sympathetic dystrophy syndrome. Pain 1988;32(1):27–34. 3340421 10.1016/0304-3959(88)90020-6

[58] Stanton-Hicks M , Burton A, Bruehl S, et al An updated interdisciplinary clinical pathway for CRPS: Report of an expert panel. Pain Pract 2002;2(1):1–16. 17134466 10.1046/j.1533-2500.2002.02009.x

[59] Baron R , Wasner G. Complex regional pain syndromes. Curr Pain Headache Rep 2001;50:114–23. 10.1007/s11916-001-0079-x 11252145

[60] Birklein F , Riedl B, Sieweke N, Weber M, Neundorfer B. Neurological findings in complex regional pain syndromes–analysis of 145 cases. Acta Neurol Scand 2000;101(4):262–9. 10770524 10.1034/j.1600-0404.2000.101004262x./

[61] Birklein F , Sittl R, Spitzer A, Claus D, Neundörfer B, Handwerker OH. Sudomotor function in sympathetic reflex dystrophy. Pain 1997;69(1):49–54. 9060012 10.1016/s0304-3959(96)03242-3

[62] Turk DC , Dworkin RH, Allen RR, et al Core outcome domains for chronic pain clinical trials: IMMPACT recommendations. Pain 2003;106(3):337–45. 14659516 10.1016/j.pain.2003.08.001

[63] Revicki D , Ehreth J. Health-related quality of life assessment and planning for the pharmaceutical industry. Clin Ther 1997;19(5):1101–15. 9385497 10.1016/s0149-2918(97)80063-x

[64] Oerlemans HM , Oostendorp RA, de Boo T, Goris RJ. Pain and reduced mobility in complex regional pain syndrome I: Outcome of a prospective randomised controlled clinical trial of adjuvant physical therapy versus occupational therapy. Pain 1999;83:77–83. 10506674 10.1016/s0304-3959(99)00080-9

[65] Glynn CJ , Basedow RW, Walsh JA. Pain relief following post-ganglionic sympathetic blockade with I.V. guanethidine. Br J Anaesth 1981;53(12):1297–302. 7317248

[66] Bonelli S , Conoscente F, Movilia PG, Restelli L, Francucci B, Grossi E. Regional intravenous guanethidine versus stellate ganglion blocks in reflex sympathetic dystrophy: A randomized trial. Pain 1983;16(3):297–307. 6350994 10.1016/0304-3959(83)90118-5

[67] Poplawski Z , Wiley A, Muñoz J. Post-traumatic dystrophy of the extremeties. J Bone Joint Surg 1983;65:642–55. 6189840

[68] Driessen JJ , Werken C, Nicolai JPA, Crui JF. Clinical effects of regional intravenous guanethide (ismelin) in reflex sympathetic dystrophy. Acta Anesth Scand 1983;27(6):505–9. 10.1111/j.1399-6576.1983.tb01996.x 6666530

[69] Baker J , Fiedler R, Ottenbacher K, Czyrny J, Heinemann A. Predicting follow-up functional outcomes in outpatient rehabilitation. Am J Phys Med Rehabil 1998;77(3):202–12. 9635555 10.1097/00002060-199805000-00004

[70] Oerlemans H , Goris J, de Boo T, Oostendorp R. Do physical therapy and occupational therapy reduce the impairment percentage in reflex sympathetic dystrophy? Am J Phys Med Rehabil 1999;78:533–9. 10574168 10.1097/00002060-199911000-00007

[71] Daly AE , Bialocerkowski AE. Does evidence support physiotherapy management of adult Complex Regional Pain Syndrome Type One? A systematic review. Eur J Pain 2009;13(4):339–53. 18619873 10.1016/j.ejpain.2008.05.003

[72] Lee BH , Scharff L, Sethna NF, et al Physical therapy and cognitive-behavioral treatment for complex regional pain syndromes. J Pediatr 2002;141(1):135–40. 12091866 10.1067/mpd.2002.124380

[73] Galer B , Jensen M. Neglect-like symptoms in complex regional pain syndrome: Results of a self-administered survey. J Pain Symptom Manage 1999;18(3):213–7. 10517043 10.1016/s0885-3924(99)00076-7

[74] Jänig W , Baron R. Experimental approach to CRPS. Pain 2004;108(1-2):3–7. 15109501 10.1016/j.pain.2004.01.005

[75] Moseley GL. Graded motor imagery is effective for long-standing complex regional pain syndrome: A randomised controlled trial. Pain 2004;108(1):192–8. 15109523 10.1016/j.pain.2004.01.006

[76] McCabe C , Haigh R, Halligan P, Blake D. Generating sensory disturbance in healthy controls. Rheumatology 2003;242:63.

[77] McCabe C , Haigh R, Ring E, Halligan P, Wall P, Blake D. A controlled pilot study of the utility of mirror visual feedback in the treatment of complex regional pain syndrome (type 1). Rheumatology (Oxford) 2002;42(1):97–101. 10.1093/rheumatology/keg041 12509620

[78] Cacchio A , De Blasis E, De Blasis V, Santilli V, Spacca G. Mirror therapy in complex regional pain syndrome type 1 of the upper limb in stroke patients. Neurorehabil Neural Repair 2009;23(8):792–9. 19465507 10.1177/1545968309335977

[79] Smart KM , Wand BM, O’Connell NE. Physiotherapy for pain and disability in adutls with complex regional pain sysndrome (CRPS) types I and II (Review). Cochrane Databsae Syst Rev 2016;(2):Art. No.CD010853. 10.1002/14651858.CD010853.pub2 PMC8646955 26905470

[80] Pleger B , Tegenthoff M, Ragert P, et al Sensorimotor retuning [corrected] in complex regional pain syndrome parallels pain reduction. Ann Neurol 2005;57(3):425–9. 15732114 10.1002/ana.20394

[81] Gay A , Parratte S, Salazard B, et al Proprioceptive feedback enhancement induced by vibratory stimulation in complex regional pain syndrome type I: An open comparative pilot study in 11 patients. Joint Bone Spine 2007;74(5):461–6. 17693114 10.1016/j.jbspin.2006.10.010

[82] David M , Dinse HR, Mainka T, Tegenthoff M, Maier C. High-frequency repetitive sensory stimulation as intervention to improve sensory loss in patients with Complex regional. Pain Syndrome I. Front. Neurol 2015;6:242. 26635719 10.3389/fneur.2015.00242 PMC4648023

[83] Melzack R , Wall PD. Pain mechanisms: A new theory. Science 1965;150(3699):971–9. 5320816 10.1126/science.150.3699.971

[84] Terkelsen AJ , Bach FW, Jensen TS. Experimental forearm immobilization in humans induces cold and mechanical hyperalgesia. Anesthesiology 2008;109:297–307. 18648239 10.1097/ALN.0b013e31817f4c9d

[85] Guo TZ , Offley SC, Boyd EA, Jacobs CR, Kingery WS. Substance P signaling contributes to the vascular and nociceptive abnormalities observed in a tibial fracture rat model of complex regional pain syndrome type I. Pain 2004;108:95–107. 15109512 10.1016/j.pain.2003.12.010

[86] McCormick ZL , Gagnon CM, Caldwell M, et al Short-term functional, emotional, and pain outcomes of patients with complex regional pain syndrome treated in a comprehensive interdisciplinary pain management program. Pain Med 2015;16(12):2357–67. 26178320 10.1111/pme.12817

[87] Vittersø AD , Buckingham G, Halicka M, Proulx MJ, Bultitude JH. Altered updating of bodily and spatial representations after tool-use in complex regional pain syndrome. Pain 2020;161(7):1609–28. 32102024 10.1097/j.pain.0000000000001845

[88] Brown CA , Scholtes I, Shenker N, Lee MC. Suboptimal learning of tactile-spatial predictions in patients with complex regional pain syndrome. Pain 2020;161(2):369–‐78. 31651574 10.1097/j.pain.0000000000001730

[89] Severens JL , Oerlemans HM, Weegels AJ, van 't Hof MA, Oostendorp RA, Goris RJ. Cost-effectiveness analysis of adjuvant physical or occupational therapy for patients with reflex sympathetic dystrophy. Arch Phys Med Rehabil 1999;80(9):1038–43. 10489005 10.1016/s0003-9993(99)90057-6

[90] Ramachandran VS , Rogers-Ramachandran D. Synaesthesia in phantom limbs induced with mirrors. Proc Biol Sci 1996;263(1369):377–86. 8637922 10.1098/rspb.1996.0058

[91] McCabe C. Mirror visual feedback therapy. A practical approach. J Hand Ther 2011;24(2):170–8. quiz 179. 21106347 10.1016/j.jht.2010.08.003

[92] Johnson S , Hall J, Barnett S, et al Using graded motor imagery for complex regional pain syndrome in clinical practice: Failure to improve pain. Eur J Pain 2012;16(4):550–‐61. 22337591 10.1002/j.1532-2149.2011.00064.x

[93] Hwang H , Cho S, Lee JH. The effect of virtual body swapping with mental rehearsal on pain intensity and body perception disturbance in complex regional pain syndrome. Int J Rehabil Res 2014;37(2):167–‐72. 24552972 10.1097/MRR.0000000000000053

[94] Phillips ME. OT treatment for complex regional pain syndrome. OT Pract 2001.

[95] Phillips ME , Katz JA, Harden RN. The use of nerve blocks in conjunction with occupational therapy for complex regional pain syndrome type I. Am J Occup Ther 2000;54(5):544–9. 11006816 10.5014/ajot.54.5.544

[96] Phillips ME , Katz J, Harden RN. Occupational and block therapies for complex regional pain syndrome. In: Midwest Pain Society–AOTA National Conference, Seattle, WA; 2000.

[97] Voss DE , Ionta MK, Myers BJ, Knott M. *Proprioceptive Neuromuscular Facilitation: Patterns and Techniques* , 3rd edition. Philadelphia: Harper & Row; 1985.

[98] Sanders SH , Harden RN, Benson SE, Vicente PJ. Clinical practice guidelines for chronic non-malignant pain syndrome patients II: An evidence-based approach. J Back Musculoskel Rehabil 1999;13(2-3):47–58. 10.3233/BMR-1995-5204 24572192

[99] State of Colorado Department of Labor and Employment. Reflex sympathetic dystrophy/complex regional pain syndrome medical treatment guidelines, 1998.

[100] Rho RH , Brewer RP, Lamer TJ, Wilson PR. Complex regional pain syndrome. Mayo Clin Proc 2002;77(2):174–80. 11838651 10.4065/77.2.174

[101] Birklein F , Handwerker HO. Complex regional pain syndrome: How to resolve the complexity? Pain 2001;94(1):1–6. 11576739 10.1016/S0304-3959(01)00393-1

[102] Goebel A , Barker C, Birklein F, et al Standards for the diagnosis and management of complex regional pain syndrome: Results of a European Pain Federation task force. Eur J Pain 2019;23(4):641–51. 30620109 10.1002/ejp.1362 PMC6593444

[103] Sherry DD , Wallace CA, Kelley C, Kidder M, Sapp L. Short- and long-term outcomes of children with complex regional pain syndrome type I treated with exercise therapy. Clin J Pain 1999;15:218–23. 10524475 10.1097/00002508-199909000-00009

[104] de Jong JR , Vlaeyen JW, Onghena P, Cuypers C, den Hollander M, Ruijgrok J. Reduction of pain-related fear in complex regional pain syndrome type I: The application of graded exposure in vivo. Pain 2005;116(3):264–75. 15964686 10.1016/j.pain.2005.04.019

[105] van de Meent H , Oerlemans M, Bruggeman A, et al Safety of “pain exposure” physical therapy in patients with complex regional pain syndrome type 1. Pain 2011;152(6):1431–8. 21474244 10.1016/j.pain.2011.02.032

[106] Travell JG , Simons DG. *Myofascial Pain and Dysfunction: The Trigger Point Manual. The Upper Extremities* . Baltimore, MD: Williams & Wilkins; 1983.

[107] Hall J , Swinkels A, Briddon J, Mccabe CS. Does aquatic exercise relieve pain in adults with neurologic or musculoskeletal disease? A systematic review and meta-analysis of randomised controlled trials. Arch Phys Med Rehabil 2008;89(5):873–83. 18452734 10.1016/j.apmr.2007.09.054

[108] O'Connell NE , Wand BM, McAuley J, Marston L, Moseley GL. Interventions for treating pain and disability in adults with complex regional pain syndrome. Cochrane Database Syst Rev 2013;2013(4):CD009416. 10.1002/14651858.CD009416.pub2 PMC6469537 23633371

[109] Smart KM , Wand BM, O'Connell NE. Physiotherapy for pain and disability in adults with complex regional pain syndrome (CRPS) types I and II. Cochrane Database Syst Rev 2016;2:CD010853. 26905470 10.1002/14651858.CD010853.pub2 PMC8646955

[110] Russ RP. The Disease. Vol. 2005. Arlington Heights, IL: ACOFP Press; 2003.

[111] Ghai B , Dureja GP. Complex regional pain syndrome: A review. J Postgrad Med 2004;50(4):300–7. 15623978

[112] Teasell RW , Bombardier C. Employment related factors in chronic pain and chronic pain disability. Clin J Pain 2001;17(Suppl 4):S39–45. 11783830 10.1097/00002508-200112001-00010

[113] Fordyce WE. Forward. In: Barber J, ed. *Psychological Approaches to the Management of Pain*. New York: Brunner/Mazel, Inc.; 1982: 5–10.

[114] Dent GL. *Return to Work…by Design* . Stockton, CA: Dennison Press; 2001.

[115] Kiralp MZ , Yildiz S, Vural D, Keskin I, Ay H, Dursun H. Effectiveness of hyperbaric oxygen therapy in the treatment of complex regional pain syndrome. J Int Med Res 2004;32(3):258–62. 15174218 10.1177/147323000403200304

[116] Taha R , Blaise GA. Update on the pathogenesis of complex regional pain syndrome: Role of oxidative stress. Can J Anaesth 2012;59(9):875–‐81. 22798149 10.1007/s12630-012-9748-y

[117] Baykal T , Seferoglu B, Karsan O, Kiziltunc A, Senel K. Antioxidant profile in patients with complex regional pain syndrome type I. Int J Rheum Dis 2014;17(2):156–8. 24576270 10.1111/1756-185X.12140

[118] Guo TZ , Wei T, Huang TT, Kingery WS, Clark JD. Oxidative stress contributes to fracture/cast-induced inflammation and pain in a rat model of complex regional pain syndrome. J Pain 2018;19(10):1147–56. 29715519 10.1016/j.jpain.2018.04.006 PMC6163064

[119] Korpan MI , Dezu Y, Schneider B, Leitha T, Fialka-Moser V. Acupuncture in the treatment of posttraumatic pain syndrome. Acta Orthop Belg 1999;65(2):197–201. 10427802

[120] Wang WY , Wan FM, Ding SQ. [Clinical observation of *Jingu* three-needle therapy combined with *Xingnao Kaiqiao* acupuncture on complex regional pain syndrome after stroke]. Zhongguo Zhen Jiu 2019;39(12):1262–6. 31820599 10.13703/j.0255-2930.2019.12.002

[121] Shearer HM , Trim A. An unusual presentation and outcome of complex regional pain syndrome: A case report. J Can Chiropr Assoc 2006;50(1):20–6. 17549166 PMC1839976

[122] Bonica JJ. The Management of Pain. Philadelphia: Lea and Feibiger; 1953

[123] Mitchell SW. *Injuries of the Nerves and Their Consequences* . Philadelphia: J.B. Lippincott & Co; 1872.

[124] Harden RN. Pharmacotherapy of complex regional pain syndrome. Am J Phys Med Rehabil 2005;84(Suppl 3):S17–28. 15722780

[125] Leriche R. De la causalgie envisagee come une nevrite du sympathique et son traitement per la denudation et l'excision des plexus nerveux periarteriels. Presse Med 1916;24:178–80.

[126] Haddox JD , Van Alstine D. Pharmacolgic therapy for reflex sympathetic dystophy. Phys Med Rehabil 1996;10:297–307.

[127] Beydoun A. Neuropathic pain: From mechanisms to treatment strategies. J Pain Symptom Manage 2003;25(Suppl 5):S1–3. 15176413 10.1016/s0885-3924(03)00063-0

[128] Finnerup NB , Attal N, Haroutounian S, McNicol E, et al Pharmacotherapy for neuropathic pain in adults: A systematic review & meta-analysis. Lancet Neurol 2015;14(2):162–73. 25575710 10.1016/S1474-4422(14)70251-0 PMC4493167

[129] Harden RN , Baron R, Janig W. Preface. In: Harden RN, Baron R, Janig W, eds. *Complex Regional Pain Syndrome*, Vol. 22. Seattle: IASP Press; 2001:xi–xiii.

[130] Treede RD , Jensen TS, Campbell JN, et al Neuropathic pain: Redefinition and a grading system for clinical and research purposes. Neurology 2008;70(18):1630–5. 18003941 10.1212/01.wnl.0000282763.29778.59

[131] Harden RN , Rudin NJ, Bruehl S. Increased systemic catecholamines in complex regional pain syndrome and relationship to psychological factors: A pilot study. Anesth Analg 2004;99:1478–85. 15502052 10.1213/01.ANE.0000132549.25154.ED

[132] Galer B , Harden R. Motor abnormalities in CRPS: A neglected but key component. In: Harden R, Baron R, Janig W, eds. *Complex Regional Pain Syndrome*, Vol. 22. Seattle: IASP Press; 2001:135–40.

[133] Goebel A , Barker C, Turner-Stoke L, et al Complex Regional Pain Syndrome in Adults, 2nd edition. RCOP; 2018.

[134] Vaneker M , Wilder-Smith OH, Schrombges P, et al Patients initially diagnosed as ‘warm’ or ‘cold’ CRPS 1 show differences in central sensory processing some eight years after diagnosis: A quantitative sensory testing study. PAIN 2005;115(1):204–11. 15836983 10.1016/j.pain.2005.02.031

[135] Geisslinger G , Yaksh T. Spinal actions of cyclooxygenase isoenzyme inhibitors. In: Devor M, Rowbotham M, Wiesenfeld-Halin Z, eds. *Proceedings of the 9th World Congress on Pain*. Seattle, WA: IASP Press; 2000:833–55.

[136] Rico H , Merono E, Gomez-Castresana F, Torrubiano J, Espinos D, Diaz P. Scintigraphic evaluation of reflex sympathetic dystrophy: Comparative study of the course of the disease under two therapeutic regimens. Clin Rheumatol 1987;6(2):233–7. 3621842 10.1007/BF02201029

[137] Birklein F , Ajit SK, Goebel A, et al Complex regional pain syndrome - phenotypic characteristics and potential biomarkers. Nat Rev Neurol 2018;14(5):272–84. 29545626 10.1038/nrneurol.2018.20 PMC6534418

[138] Parry GJ , Kozu H. Piroxicam may reduce the rate of progression of experimental diabetic neuropathy. Neurology 1990;40(9):1446–9. 2392233 10.1212/wnl.40.9.1446

[139] Dray A. Inflammatory mediators of pain. Br J Anaesth 1995;75(2):125–31. 7577246 10.1093/bja/75.2.125

[140] Pappagallo M , Rosenberg A. Epidemiology, pathophysiology, and management of complex regional paidrome. Pain Practice 2001;1(1):11–20. 17129280 10.1046/j.1533-2500.2001.01003.x

[141] Daffonchio L , Rossoni G, Clavenna G, et al Protective activity of ketoprofen lysine salt against the pulmonary effects induced by bradykinin in guinea-pigs. Inflamm Res 1996;45(5):259–64. 8737750 10.1007/BF02259613

[142] Christensen K. The reflex dystrophy syndrome response to treatment with systemic corticosteroids. Acta Chir Scand 1982;653–5. 6763435

[143] Boughton-Smith NK , Whittle BJ. Stimulation and inhibition of prostacyclin formation in the gastric mucosa and ileum in vitro by anti-inflammatory agents. Br J Pharmacol 1983;78(1):173–80. 6402044 10.1111/j.1476-5381.1983.tb09378.x PMC2044797

[144] Hennekens C , Borzak S. Cyclooxygenase-2 Inhibitors and most traditional non-steroidal Anti-inflammatory drugs cause similar moderately increased risks of cardiovascular disease. JCPT. 10.1177/1074248407312990 18287589

[145] Braus DF , Krauss JK, Strobel J. The shoulder hand syndrome after stroke: A prospective clinical trial. Ann Neurol 1994;36(5):728–44. 7526774 10.1002/ana.410360507

[146] Forouzanfar T , Koke A, van Kleef M, Weber W. Treatment of complex regional pain syndrome type 1. Eur J Pain 2002;6(2):105–22. 11900471 10.1053/eujp.2001.0304

[147] Yasir M , Goyal A, Bansal P, Sonthalia S. Corticosteroid adverse effects. In: StatPearls. Treasure Island (FL): StatPearls Publishing; 2021 . PMID 30285357 30285357

[148] Barbalinardo S , Loer SA, Goebel A. The treatment of longstanding complex regional pain syndrome with oral steroids. Pain Med 2016;17(2):337–43. 26814238 10.1093/pm/pnv002

[149] Munts AG , Van der plas AA, et al Efficacy and safety of a single intrathecal methylprednisolone bolus in chronic complex regional pain syndrome. Euro J Pain 2010;14(5):523–8. 10.1016/j.ejpain.2009.11.004 20018535

[150] Goebel A , Baranowski A, Maurer K, Ghiai A, McCabe C, Ambler G. Intravenous immunoglobulin treatment of the complex regional pain syndrome: A randomized trial. Annals of Internal Medicine 2010;152(3):152–8. 20124231 10.7326/0003-4819-152-3-201002020-00006

[151] Goebel A , Bisla J, Carganillo R, Frank B, Gupta R, et al Low-dose intravenous immunoglobulin treatment for long-standing complex regional pain syndrome: A randomized trial. 2017. Ann Intern Med 2017;167(7):476–83. 28973211 10.7326/M17-0509

[152] Dirckx M , Groeneweg G, Wesseldijk F, Stronks DL, Huygen FJPM. Report of a preliminary discontinued double-blind, randomized, placebo-controlled trial of the anti-TNF-α chimeric monoclonal antibody infliximab in complex regional pain syndrome. Pain Pract. Isuue 2013;13(8):633–40. 10.1111/papr.12078 23692303

[153] Manning DC , Alexander G, Arezzo JC, et al Lenalidomide for Complex Regional Pain Syndrome Type 1: Lack of Efficacy in a Phase II Randomized Study. Journal of Pain 2014;15(12):1366–76. 25283471 10.1016/j.jpain.2014.09.013

[154] McCleane GJ. Lamotrigine in the management of neuropathic pain: A review of the literature. Clin J Pain 2000;16(4):321–6. 11153788 10.1097/00002508-200012000-00008

[155] Goebel A , Jacob A, Sacco P, et al Mycophenolate for persistent complex regional pain syndrome, a parallel, open, randomised, proof of concept trial. Scandanavian Journal of Pain 2018;18(1):29–37. 10.1515/sjpain-2017-0154 29794285

[156] Aradillas E , Goebel A, Schwartzman RJ, et al Plasma Exchange Therapy in Patients with Complex Regional Pain Syndrome. Pain Physician 2015;18(4):383–94. 26218942

[157] Schwartz J , Padmanabhan A, Aqui N. Guidelines on the use of therapeutic apheresis in clinical practice-evidence-based approach from the writing committee of the American Society for Apheresis: The seventh special issue. J Clin Apher 2016;149–62. 27322218 10.1002/jca.21470

[158] Kersten C , Cameron MG, Bailey AG, et al Relief of neuropathic pain through epidermal growth factor receptor inhibition: A randomized proof-of-concept trial. Pain Med 2019;20(12):2495–505. 31106835 10.1093/pm/pnz101

[159] van der Laan L , Veldman P, Goris JA. Severe complications of reflex sympathetic dystrophy: Infection, ulcers, chronic edema, dystonia, myoclonus. Arch Phys Med Rehabil 1998;79(4):424–9. 9552109 10.1016/s0003-9993(98)90144-7

[160] Helyes Z , Tekus V, Szentes N, et al Transfer of complex regional pain syndrome to mice via human autoantibodies is mediated by interleukin-1-induced mechanisms. Proc Natl Acad Sci USA 2019;116(26):13067–76. 31182576 10.1073/pnas.1820168116 PMC6600930

[161] Guo T , Wei T, Tajerian M, et al Complex regional pain syndrome patient immunoglobulin M has pronociceptive effects in the skin and spinal cord of tibia fracture mice. PAIN 2020;161(4):797–809. 31815913 10.1097/j.pain.0000000000001765 PMC7269192

[162] Brunner F , Schmid A, Kissling R, et al Biphosphonates for the therapy of complex regional pain syndrome I–systematic review. Eur J Pain 2009;13(1):17–21. 18440845 10.1016/j.ejpain.2008.03.005

[163] Vannala V , Palaian S, Shankar PR. Therapeutic dimensions of biphosphonates: A clinical update. Int J Prev Med 2020;11:166. 33312475 10.4103/ijpvm.IJPVM_33_19 PMC7716604

[164] Varenna M , Adami S, Rossini M, et al Treatment of complex regional pain syndrome type I with neridronate: A randomized, double-blind, placebo-controlled study. Rheumatology 2013;52(3):534–42. 23204550 10.1093/rheumatology/kes312

[165] Sindrup SH , Jensen TS. Efficacy of pharmacological treatments of neuropathic pain: An update and effect related to mechanism of drug action. Pain 1999;83(3):389–400. 10568846 10.1016/S0304-3959(99)00154-2

[166] Collins SL , Moore RA, McQuay H, Wiffen P. Antidepressants and anticonvulsants for diabetic neuropathy and postherpetic neuralgia: A quantitative systematic review. J Pain Symptom Manage 2000;20:449–58. 11131263 10.1016/s0885-3924(00)00218-9

[167] McQuay H , Carroll D, Jadad AR, Wiffen P, Moore A. Anticonvulsant drugs for management of pain: A systematic review. BMJ 1995;311(7012):1047–52. 7580659 10.1136/bmj.311.7012.1047 PMC2551361

[168] Wiffen P , Collins S, McQuay H, Carroll D, Jadad A, Moore A. Anticonvulsant drugs for acute and chronic pain. Cochrane Database Syst Rev 2000;CD001133. 10.1002/14651858.CD001133 10908487

[169] Hord ED , Oaklander AL. Complex regional pain syndrome: A review of evidence-supported treatment options. Curr Pain Headache Rep 2003;7(3):188–96. 12720598 10.1007/s11916-003-0072-7

[170] Mellick GA , Mellick LB. Reflex sympathetic dystrophy treated with gabapentin. Arch Phys Med Rehabil 1997;78(1):98–105. 9014968 10.1016/s0003-9993(97)90020-4

[171] Rowbotham M , Harden N, et al Gabapentin for the treatment of neuralgia: A randomized controlled trial. JAMA 1998;280(21):1837–42. 9846778 10.1001/jama.280.21.1837

[172] Backonja M , Beydoun A, Edwards KR, et al Gabapentin for the symptomatic treatment of painful neuropathy in patients with diabetes mellitus. JAMA 1998;280(21):1831–6. 9846777 10.1001/jama.280.21.1831

[173] Wheeler DS , Vaux KK, Tam DA. Use of gabapentin in the treatment of childhood reflex sympathetic dystrophy. Pediatr Neurol 2000;22(3):220–1. 10734253 10.1016/s0887-8994(99)00139-3

[174] Van, de V , Goossens VJ, Kemler MA, et al Screening of patients with Complex Regional Pain Syndrome for antecedent infections. Clin J Pain 2001;17(2):110–4. 10.1097/00002508-200106000-00002 11444711

[175] Burchiel KJ. Carbamazepine inhibits spontaneous activity in experimental neuromas. Exp Neurol 1988;102(2):249–53. 3181365 10.1016/0014-4886(88)90101-x

[176] Rull JA , Quibrera R, González-Millán H, Castañeda OL. Symptomatic treatment of peripheral diabetic neuropathy with carbamizepine: Double-blind crossover study. Diabetologia 1969;5(4):215–20. 4902717 10.1007/BF01212087

[177] Harke H , Gretenkort P, Ladleif HU, Rahman S, Harke O. The response of neuropathic pain and pain in complex regional pain syndrome I to carbamazepine and sustained-release morphine in patients pretreated with spinal cord stimulation: A double-blinded randomized study. Anesth Analg 2001;92(2):488–95. 11159256 10.1097/00000539-200102000-00039

[178] Simon S. Oxcarbazepine: A review. Seizure 2000;9:75–9. 10845729 10.1053/seiz.2000.0391

[179] Chadda V , Mathur M. Double blind study of the effects of diphenylhydantoin sodium on diabetic neuropathy. J Assoc India 1978;26:403–6. 365857

[180] Matzner O , Devor M. Na+ conductance and the threshold for repetitive neuronal firing. Brain Res 1992;597(1):92–8. 1335824 10.1016/0006-8993(92)91509-d

[181] Yaari Y , Devor M. Phenytoin suppresses spontaneous discharge in rat sciatic nerve neuromas. Neuroscince Lett 1985;4:117–22. 10.1016/0304-3940(85)90339-8 4047470

[182] Sindrup SH , Jensen TS. Pharmacologic treatment of pain in polyneuropathy. Neurology 2000;55(7):915–20. 11061244 10.1212/wnl.55.7.915

[183] Woolf CJ , Max MB. Mechanism-based pain diagnosis: Issues for analgesic drug development. Anesthesiology 2001;95(1):241–9. 11465563 10.1097/00000542-200107000-00034

[184] Max MB. Thirteen consecutive well-designed randomized trials show that antidepressants reduce pain in diabetic neuropathy and postherpetic neuralgia. Pain Forum 1995;4(4):248–53.

[185] Max MB , Kishore-Kumar R, Schafer SC, et al Efficacy of desipramine in painful diabetic neuropathy: A placebo-controlled trial. Pain 1991;45(1):3–9. 1861872 10.1016/0304-3959(91)90157-S

[186] McQuay HJ , Tramer M, Nye BA, Carroll D, Wiffen PJ, Moore RA. A systematic review of antidepressants in neuropathic pain. Pain 1996;68(2-3):217–27. 9121808 10.1016/s0304-3959(96)03140-5

[187] Watson CPN , Chipman M, Reed K, Evans RJ, Birkett N. Amitriptyline versus maprotiline in postherpetic neuralgia: A randomized, double-blind, crossover trial. Pain 1992;48(1):29–36. 1738571 10.1016/0304-3959(92)90128-X

[188] Max MB , Lynch SA, Muir J, Shoaf SE, Smoller B, Dubner R. Effects of desipramine, amitriptyline, and fluoxetine on pain in diabetic neuropathy. N Engl J Med 1992;326(19):1250–6. 1560801 10.1056/NEJM199205073261904

[189] Sindrup SH , Bjerre U, Dejgaard A, Brøsen K, Aaes-Jørgensen T, Gram LF. The selective serotonin reuptake inhibitor citalopram relieves the symptoms of diabetic neuropathy. Clin Pharmacol Ther 1992;52(5):547–52. 1424428 10.1038/clpt.1992.183

[190] Sindrup SH , Gram LF, Brosen K, Eshoj O, Mogensen EF. The selective serotonin reuptake inhibitor paroxetine is effective in the treatment of diabetic neuropathy symptoms. Pain 1990;42(2):135–44. 2147235 10.1016/0304-3959(90)91157-E

[191] Cherny NI , Thaler HT, Friedlander-Klar H, et al Opioid responsiveness of cancer pain syndromes caused by neuropathic or nociceptive mechanisms: A combined analysis of controlled, single-dose studies. Neurology 1994;44(5):857–61. 7514771 10.1212/wnl.44.5.857

[192] Portenoy R , Foley K, Inturrisi C. The nature of opioid responsiveness and its implications for neuropathic pain: New hypotheses derived from studies for opioid infusions. Pain 1990;43(3):273–86. 1705692 10.1016/0304-3959(90)90025-9

[193] Dellemijn P. Are opioids effective in relieving neuropathic pain? Pain 1999;80(3):453–62. 10342407 10.1016/S0304-3959(98)00256-5

[194] Dunn KM , Saunders KW, Rutter CM, et al Opioid prescriptions for chronic pain and overdose: A cohort study. Ann Intern Med 2010;152(2):85–92. 20083827 10.1059/0003-4819-152-2-201001190-00006 PMC3000551

[195] Ebert B , Andersen S, Krogsgaard-Larsen P. Ketobemidone, methadone and pethidine are non-competitive N-methyl-D-aspartate (NMDA) antagonists in the rat cortex and spinal cord. Neurosci Lett 1995;187(3):165–8. 7624018 10.1016/0304-3940(95)11364-3

[196] Roulet L , Rollason V, Desmeules J, Piguet V. Tapentadol versus tramadol: A narrative and comparative review of their pharmacological, efficacy and safety profiles in adult patients. Drugs 2021;81(11):1257–72. PMID 34196947 34196947 10.1007/s40265-021-01515-z PMC8318929

[197] Harden RN , Bruehl S, Backonja M-M. The use of opioids in treatment of chronic pain: An examination of the ongoing controversy. J Back Musculoskelet Rehab 1997;9(2):155–80. 10.3233/BMR-1997-9207 24573007

[198] Harden RN , Bruehl S, Siegler J, Cole PA. Pain, psychological status, and functional recovery in chronic pain patients on daily opioids: A case comparison. Journal of Back and Musculoskeletal Rehabilitation 1997;9(2):101–8. 24573003 10.3233/BMR-1997-9203

[199] Mao J , Price D, Caruso F, Mayer D. Oral administration of dexromethorphan prevents the development of morphine tolerance and dependence in rats. Pain 1996;67(2):361–8. 8951930 10.1016/0304-3959(96)03120-x

[200] Mitchell SW , Morehouse GR, Keen WW. Gunshot wounds and other injuries of nerves. 1864. Clin Orthop Relat Res 2007;458:35–9. 17473596 10.1097/BLO.0b013e31803df02c

[201] Eide PK , Jorum E, Stubhaug A, Bremnes J, Breivik H. Relief of post-herpetic neuralgia with the N-methyl-D-aspartic acid receptor antagonist ketamine: A double-blind, cross-over comparison with morphine and placebo. Pain 1994;58(3):347–54. 7838584 10.1016/0304-3959(94)90129-5

[202] Mao J , Price DD, Mayer DJ, Lu J, Hayes RL. Intrathecal MK-801 and local nerve anesthesia synergistically reduce nociceptive behaviors in rats with experimental peripheral mononeuropathy. Brain Res 1992;576(2):254–62. 1325239 10.1016/0006-8993(92)90688-6

[203] Nelson KA , Park KM, Robinovitz E, Tsigos C, Max MB. High dose oral dextromethorphan versus placebo in painful diabetic neuropathy and postherpetic neuralgia. Neurology 1997;48(5):1212–8. 9153445 10.1212/wnl.48.5.1212

[204] Qian J , Brown SD, Carlton SM. Systemic ketamine attenuates nociceptive behaviors in a rat model of peripheral neuropathy. Brain Res 1996;715(1-2):51–62. 8739622 10.1016/0006-8993(95)01452-7

[205] Tal M , Bennett GJ. Dextromorphan relieves neuropathic heat-evoked hyperalgesia. Neurosci Lett 1993;151(1):107–10. 8385757 10.1016/0304-3940(93)90058-s

[206] Hunt JA , Lake MA. Reviewing the physiology, pharmacology and therapeutic uses of ketamine. Nurs Stand 2021;36(9):77–81. PMID 34423608 10.7748/ns.2021.e11737 34423608

[207] Schwartzman RJ , Alexander GM, Grothusen JR, Paylor T, Reichenberger E, Perreault M. Outpatient intravenous ketamine for the treatment of complex regional pain syndrome: A double-blind placebo controlled study. Pain 2009;147(1):107–15. 19783371 10.1016/j.pain.2009.08.015

[208] Sigtermans MJ , Van Hilten JJ, Bauer MC. Ketamine produces effective and long-term pain relief in patients with Complex Regional Pain Syndrome. Pain 2009;10:304–11. 10.1016/j.pain.2009.06.023 19604642

[209] Jayaseekan S , Sachan K, Gupta M, et al Racemic Ketamine 4.5 day infusion treatment of long standing Complex Regional Pain Syndrome- a prospective service evaluation in five patients. Br J Anaesth 2015;115(1):146–7 26089467 10.1093/bja/aev183

[210] Pickering AE , McCabe CS. Prolonged Ketamine infusion as a therapy for Complex regional pain syndrome: Synergism with antagonism? Br J Clin Pharmacol 2014;77(2):233–8. 23701138 10.1111/bcp.12157 PMC3992840

[211] Connolly SB , Prager JP, Harden RN, et al A systematic review of Ketamine for complex regional pain syndrome. Pain Med 2015;16(5):943–69. 25586192 10.1111/pme.12675

[212] Pud D , Eisenberg E, Spitzer A, Adler R, Fried G, Yarnitsky D. The NMDA receptor antagonist amantadine reduces surgical neuropathic pain in cancer patients: A double blind, randomized, placebo controlled trial. Pain 1998;75(2–3):349–54. 9583771 10.1016/s0304-3959(98)00014-1

[213] Eisenberg E , Pud D. Can patients with chronic neuropathic pain be cured by acute administration of the NMDA receptor antagonist amantadine? Pain 1998;74(2–3):337–9. 9520249 10.1016/s0304-3959(97)00198-x

[214] Gustin SM , Schwarz A, Birbaumer N, et al NMDA-receptor antagonist and morphine decrease CRPS-pain and cerebral pain representation. Pain 2010;151(1):69–76. 20630656 10.1016/j.pain.2010.06.022

[215] Goebel A. Morphine and memantine treatment of long-standing Complex Regional Pain Syndrome. Pain Med 2012;13(3):357–8. 22295900 10.1111/j.1526-4637.2011.01317.x

[216] Tracey DJ , Cunningham JE, Romm MA. Peripheral hyperalgesia in experimental neuropathy: Mediation by alpha-2 and renoreceptors on post-ganglionic sympathetic terminals. Pain 1995;60(3):317– 27. 7596628 10.1016/0304-3959(94)00141-z

[217] Rauck R , Eisenach J, Jackson K, Young L, Southern J. Epidural clonidine for refractory reflex sympathetic dystrophy. Anesthesiology 1993;79(6):1163–9. 8267190

[218] Davis KD , Treede RD, Raja SN, Meyer RA, Campbell JN. Topical application of clonidine relieves hyperalgesia in patients with sympathetically maintained pain. Pain 1991;47(3):309–17. 1664508 10.1016/0304-3959(91)90221-I

[219] Muizelaar JP , Kleyer M, Hertogs IA, DeLange DC. Complex regional pain syndrome (reflex sympathetic dystrophy and causalgia): management with the calcium channel blocker nifedipine and/or the alpha-sympathetic blocker phenoxybenzamine in 59 patients. Clin Neurol Neurosurg 1997;99(1):26–30. 9107464 10.1016/s0303-8467(96)00594-x

[220] Prough DS , McLeskey CH, Poehling GG, et al Efficacy of oral nifedipine in the treatment of reflex sympathetic dystrophy. Anesthesiology 1985;62(6):796–9. 4003801 10.1097/00000542-198506000-00017

[221] Ohta S , Tanahashi T, Iida H, et al [A case report of reflex sympathetic dystrophy treated with nifedipine.]. Masui 1989;38(5):679–83. Japanese. PMID2778955 2778955

[222] Ghostine SY , Comair YG, Turner DM, Kassell NF, Azar CG. Phenoxybenzamine in the treatment of causalgia. Report of 40 cases. J Neurosurg 1984;60(6):1263–8. 6726371 10.3171/jns.1984.60.6.1263

[223] Braga PC. Calcitonin and its antinociceptive activity: Animal and human investigations 1975-1992. Agents Actions 1994;41(3-4):121–31. 7942319 10.1007/BF02001904

[224] Bickerstaff DR , Kanis JA. The use of nasal calcitonin in the treatment of post-traumatic algodystrophy. Br J Rheum 1991;30(4):291–4. 10.1093/rheumatology/30.4.291 1863827

[225] Gobelet C , Meier JL, Schaffner W, Bischof-Delaloye A, Gerster JC, Burckhardt P. Calcitonin and reflex sympathetic dystrophy syndrome. Clin Rheum 1986;5(3):382–8. 10.1007/BF02054258 3536262

[226] Gobelet C , Waldburger M, Meier JL. The effect of adding calcitonin to physical treatment on reflex sympathetic dystrophy. Pain 1992;48(2):171–5. 1589234 10.1016/0304-3959(92)90055-G

[227] Perez R , Kwakkel G, Zuurmond W, de Lange J. Treatment of reflex sympathetic dystrophy (CRPS type I): A research synthesis of 21 randomized clinical trials. J Pain Sympt Management 2001;21(6):511–26. 10.1016/s0885-3924(01)00282-2 11397610

[228] Cherot A , Amor B. [Treatment of algodystrophy. A randomized study of 95 cases with 3 treatments: Calsyn 100, Visken, Grisefuline, and Penthonium]. Rev Rhum Mal Osteoartic 1983;50(2):95–7. 6134331

[229] van Hilten JJ. Movement disorders in complex regional pain syndrome. Pain Med 2010;11(8):1274–7. 20704676 10.1111/j.1526-4637.2010.00916.x

[230] van Hilten BJ , van de Beek W-JT, Hoff JI, Voormolen JHC, Delhaas EM. Intrathecal baclofen for the treatment of dystonia in patients with reflex sympathetic dystrophy. N Engl J Med 2000;343(9):625–30. 10965009 10.1056/NEJM200008313430905

[231] Guttmann O , Wykes V. Images in clinical medicine. Complex Regional Pain Syndrome type 1. N Engla J Med 2008;359(5):508. 10.1056/NEJMicm060173 18669429

[232] Rog DJ , Nurmikko TJ, Friede T, Young CA. Randomized, controlled trial of cannabis-based medicine in central pain in multiple sclerosis. Neurology 2005;65(6):812–9. 16186518 10.1212/01.wnl.0000176753.45410.8b

[233] Almog S , Aharon‐Peretz J, Vulfsons S, et al The pharmacokinetics, efficacy and safety of a novel selective-dose cannabis inhaler in patients with chronic pain. A randomized, double-blinded, placebo-controlled trial. Eur J Pain 2020;24(8):1505–16. 32445190 10.1002/ejp.1605 PMC7496774

[234] Marsili L , Bologna M, Jankovic J, Colosimo C. Long-term efficacy and safety of botulinum toxin treatment for cervical dystonia: A critical reappraisal. Expert Opin Drug Saf 2021;20(6):695–705. 33831328 10.1080/14740338.2021.1915282

[235] Ranoux D , Attal N, Morain F, Bouhassira D. Botulinum toxin type A induces direct analgesic effects in chronic neuropathic pain. Ann Neurol 2008;64(3):274–83. 18546285 10.1002/ana.21427

[236] Carroll I , Clark JD, Mackey S. Sympathetic block with botulinum toxin to treat complex regional pain syndrome. Ann Neurol 2009;65(3):348–51. 19334078 10.1002/ana.21601 PMC2763598

[237] Attal N , Brasseur L, Chauvin M, Bouhassira D. Effects of single and repeated applications of a eutectic mixture of local anaesthetics (EMLA) cream on spontaneous and evoked pain in post-herpetic neuralgia. Pain 1999;81(1-2):203–9. 10353509 10.1016/s0304-3959(99)00014-7

[238] Galer BS , Rowbotham MC, Perander J, Friedman E. Topical lidocaine patch relieves postherpetic neuralgia more effectively than a vehicle topical patch: Results of an enriched enrollment study. Pain 1999;80(3):533–8. 10342414 10.1016/S0304-3959(98)00244-9

[239] Rowbotham MC , Davies PS, Verkempinck C, Galer BS. Lidocaine patch: Double-blind controlled study of a new treatment method for postherpetic neuralgia. Pain 1996;65(1):39– 44. 8826488 10.1016/0304-3959(95)00146-8

[240] Chang A , Rosani A, Quick J. Capsaicin. In: StatPearls. Treasure Island, FL: StatPearls Publishing; 2021. PMID 29083760. 29083760

[241] Watson CP , Tyler KL, Bickers DR, Millikan LE, Smith S, Coleman E. A randomized vehicle-controlled trial of topical capsaicin in the treatment of postherpetic neuralgia. Clinical Therapeutics 1993;15(3):510–26. 8364943

[242] Robbins WR , Staats PS, Levine J, et al Treatment of intractable pain with topical large-dose capsaicin: Preliminary report. Anesth Analg 1998;86(3):579–83. 9495419 10.1097/00000539-199803000-00027

[243] Bernstein JE , Korman NJ, Bickers DR, Dahl MV, Millikan LE. Topical capsaicin treatment of chronic postherpetic neuralgia. J Am Acad Dermatil 1989;21(2):265–70. 10.1016/s0190-9622(89)70171-7 2768576

[244] Capsaicin Study Group. Treatment of painful diabetic neuropathy with topical capsaicin: A mulitcenter, double-blind, vehicle-controlled study. Arch Intern Med 1991;151:2225–9. 1953227 10.1001/archinte.151.11.2225

[245] Peikert A , Hentrich M, Ochs G. Topical 0.025% capsaicin in chronic post-herpetic neuralgia: Efficacy, predictors of response and long-term course. J Neurol 1991;238(8):452–6. 1779253 10.1007/BF00314653

[246] Simone DA , Ochoa J. Early and late effects of prolonged topical capsaicin on cutaneous sensibility and neurogenic vasodilation in humans. Pain 1991;47(3):285–94. 1784499 10.1016/0304-3959(91)90217-L

[247] Backonja M , Wallace MS, Blonsky ER, et al NGX-4010, a high-concentration capsaicin patch, for the treatment of postherpetic neuralgia: A randomised, double-blind study. Lancet Neurology 2008;7(12):1106–12. 18977178 10.1016/S1474-4422(08)70228-X

[248] Simpson DM , Brown S, Tobias J; NGX-4010 C107 Study Group. Controlled trial of high-concentration capsaicin patch for treatment of painful HIV neuropathy. Neurology 2008;70(24):2305–13. 18541884 10.1212/01.wnl.0000314647.35825.9c

[249] Simpson DM , Gazda S, Brown S, NGX-4010 C118 Study Group, et al Long-term safety of NGX-4010, a high-concentration capsaicin patch, in patients with peripheral neuropathic pain. J Pain Symptom Manage 2010;39(6):1053–64. 20538187 10.1016/j.jpainsymman.2009.11.316

[250] Girtler R , Kloimstein H, Gustorff B, et al Pronounced symptom deterioration in complex regional pain syndrome type 2 after isolated application of a highly concentrated capsaicin patch. A case report. Schmerz 2013;27(1):67–71. 23229516 10.1007/s00482-012-1268-8

[251] Zuurmond WWA , Langendijk PNJ, Bezemer PD, Brink HEJ, Lange JJ, Loenen AC. Treatment of acute reflex sympathetic dystrophy with DMSO 50% in a fatty cream. Acta Anaesthesiol Scand 1996;40(3):364–7. 8721469 10.1111/j.1399-6576.1996.tb04446.x

[252] Zollinger PE , Tuinebreijer WE, Kreis RW, Breederveld RS. Effect of vitamin C on frequency of reflex sympathetic dystrophy in wrist fractures: A randomized trial. Lancet 1999;354(9195):2025–8. 10636366 10.1016/S0140-6736(99)03059-7

[253] Shibuya N , Humphers JM, Agarwal MR, Jupiter DC. Efficacy and safety of high dose vitamin C on complex regional pain syndrome in extremity trauma and surgery-systematic review and meta-analysis. J Foot Ankle Surg 2013;52(1):62–6. 22985495 10.1053/j.jfas.2012.08.003

[254] Evaniew N , McCarthy C, Kleinlugtenbelt YV, Ghert M, Bhandari M. Vitamin C to prevent complex regional pain syndrome in patients with distal radius fractures: A meta-analysis of randomized controlled trials. J Orthop Trauma 2015;29(8):e235–41. PMID26197022 26197022 10.1097/BOT.0000000000000305

[255] Grabow TS , Christo PJ, Raja SN. Complex regional pain syndrome: Diagnostic controversies, psychological dysfunction, and emerging concepts. Adv Psychosom Med 2004;25:89–101. 15248369 10.1159/000079060

[256] Bruehl S. An update on the pathophysiology of complex regional pain syndrome. Anesthesiology 2010;113(3):713–25. 20693883 10.1097/ALN.0b013e3181e3db38

[257] Bruehl S. Complex regional pain syndrome. BMJ 2015;351:h2730. 26224572 10.1136/bmj.h2730

[258] Harden RN , Duc TA, Williams TR, Coley D, Cate JC, Gracely RH. Norepinephrine and epinephrine levels in affected versus unaffected limbs in sympathetically maintained pain. Clin J Pain 1994;10(4):324–30. 7858364 10.1097/00002508-199412000-00014

[259] Birklein F , Riedl B, Claus D, Neundorfer B. Pattern of autonomic dysfunction in time course of complex regional pain syndrome. Clin Auton Res 1998;8(2):79–85. 9613797 10.1007/BF02267817

[260] Kurvers H , Daemen M, Slaaf D. Partial peripheral neuropathy and denervation induced adrenoceptor supersensitivity: Functional studies in an experimental model. Acta Orthopaedica Belgica 1998;64:64–70. 9586253

[261] Arnold JM , Teasell RW, MacLeod AP, Brown JE, Carruthers SG. Increased venous alpha-adrenoceptor responsiveness in patients with reflex sympathetic dystrophy. Annals of Internal Medicine 1993;118(8):619–21. 8383935 10.7326/0003-4819-118-8-199304150-00008

[262] Baron R , Maier C. Reflex sympathetic dystrophy: Skin blood flow, sympathetic vasoconstrictor reflexes and pain before and after surgical sympathectomy. Pain 1996;67(2):317–26. 8951925 10.1016/0304-3959(96)03136-3

[263] Oaklander AL , Rissmiller JG, Gelman LB, Zheng L, Chang Y, Gott R. Evidence of focal small-fiber axonal degeneration in complex regional pain syndrome-I (reflex sympathetic dystrophy). Pain 2006;120(3):235–43. 16427737 10.1016/j.pain.2005.09.036

[264] Siegel SM , Lee JW, Oaklander AL. Needlestick distal nerve injury in rats models symptoms of complex regional pain syndrome. Anesth Analg 2007;105(6):1820–9. 18042888 10.1213/01.ane.0000295234.21892.bc

[265] Drummond PD , Finch PM, Skipworth S, Blockey P. Pain increases during sympathetic arousal in patients with complex regional pain syndrome. Neurology 2001;57(7):1296–303. 11591852 10.1212/wnl.57.7.1296

[266] Drummond ES , Maker G, Birklein F, Finch PM, Drummond PD. Topical prazosin attenuates sensitivity to tactile stimuli in patients with complex regional pain syndrome. Eur J Pain 2016;20(6):926–35. 26568465 10.1002/ejp.817

[267] Drummond PD , Morellini N, Finch PM, Birklein F, Knudsen LF. Complex regional pain syndrome: Intradermal injection of phenylephrine evokes pain and hyperalgesia in a subgroup of patients with upregulated alpha1-adrenoceptors on dermal nerves. Pain 2018;159(11):2296–305. 29994991 10.1097/j.pain.0000000000001335

[268] Gracely RH , Lynch SA, Bennett GJ. Painful neuropathy: Altered central processing maintained dynamically by peripheral input. Pain 1992;51(2):175–94. 1484715 10.1016/0304-3959(92)90259-E

[269] Woolf CJ , Shortland P, Coggeshall RE. Peripheral nerve injury triggers central sprouting of myelinated afferents. Nature 1992;355(6355):75–8. 1370574 10.1038/355075a0

[270] Charney DS , Woods SW, Nagy LM, Southwick SM, Krystal JH, Heninger GR. Noradrenergic function in panic disorder. J Clin Psychiatry 1990;51 Suppl A:5–11. 2258377

[271] Light KC , Kothandapani RV, Allen MT. Enhanced cardiovascular and catecholamine responses in women with depressive symptoms. Int J Psychophysiol 1998;28(2):157–66. 9545653 10.1016/s0167-8760(97)00093-7

[272] Edwards RR , Kronfli T, Haythornthwaite JA, Smith MT, McGuire L, Page GG. Association of catastrophizing with interleukin-6 responses to acute pain. Pain 2008;140(1):135–44. 18778895 10.1016/j.pain.2008.07.024 PMC2659503

[273] Kaufmann I , Eisner C, Richter P, et al Lymphocyte subsets and the role of TH1/TH2 balance in stressed chronic pain patients. Neuroimmunomodulation 2007;14(5):272–80. 18239379 10.1159/000115041

[274] Beerthuizen A , van 't Spijker A, Huygen FJ, Klein J, de Wit R. Is there an association between psychological factors and the Complex Regional Pain Syndrome type 1 (CRPS1) in adults? A systematic review. Pain 2009;145(1-2):52–9. 19573987 10.1016/j.pain.2009.05.003

[275] Puchalski P , Zyluk A. Complex regional pain syndrome type 1 after fractures of the distal radius: A prospective study of the role of psychological factors. J Hand Surg 2005;30(6):574–80. 10.1016/j.jhsb.2005.06.023 16126313

[276] Beerthuizen A , Stronks DL, Huygen FJ, Passchier J, Klein J, Spijker AV. The association between psychological factors and the development of complex regional pain syndrome type 1 (CRPS1)–a prospective multicenter study. Eur J Pain 2011;15(9):971–5. 21459637 10.1016/j.ejpain.2011.02.008

[277] Harden RN , Bruehl S, Stanos S, et al Prospective examination of pain-related and psychological predictors of CRPS-like phenomena following total knee arthroplasty: A preliminary study. Pain 2003;106(3):393–400. 14659522 10.1016/j.pain.2003.08.009

[278] Dijkstra PU , Groothoff JW, ten Duis HJ, Geertzen JH. Incidence of complex regional pain syndrome type I after fractures of the distal radius. Eur J Pain 2003;7(5):457–62. 12935798 10.1016/s1090-3801(03)00015-6

[279] Van Houdenhove B , Vasquez G, Onghena P, et al Etiopathogenesis of reflex sympathetic dystrophy: A review and biopsychosocial hypothesis. Clin J Pain 1992;8(4):300–6. 1493341 10.1097/00002508-199212000-00004

[280] Egle UT , Hoffmann SO. [Psychosomatic correlations of sympathetic reflex dystrophy (Sudeck's disease). Review of the literature and initial clinical results]. Psychother Psychosom Med Psychol 1990;40:123–35. 1692417

[281] Geertzen JH , de Bruijn-Kofman AT, de Bruijn HP, van de Wiel HB, Dijkstra PU. Stressful life events and psychological dysfunction in Complex Regional Pain Syndrome type I. Clin J Pain 1998;14(2):143–7. 9647456 10.1097/00002508-199806000-00009

[282] Wager J , Brehmer H, Hirschfeld G, Zernikow B. Psychological distress and stressful life events in pediatric complex regional pain syndrome. Pain Res Manag 2015;20(4):189–94. 26035287 10.1155/2015/139329 PMC4532204

[283] Speck V , Schlereth T, Birklein F, Maihöfner C. Increased prevalence of posttraumatic stress disorder in CRPS. Eur J Pain 2017;21(3):466–‐73. 27650922 10.1002/ejp.940

[284] Reedijk WB , van Rijn MA, Roelofs K, Tuijl JP, Marinus J, van Hilten JJ. Psychological features of patients with complex regional pain syndrome type I related dystonia. Mov Disord 2008;23(11):1551–9. 18546322 10.1002/mds.22159

[285] Monti DA , Herring CL, Schwartzman RJ, Marchese M. Personality assessment of patients with complex regional pain syndrome type I. Clin J Pain 1998;14(4):295–302. 9874007 10.1097/00002508-199812000-00005

[286] Rommel O , Malin JP, Zenz M, Janig W. Quantitative sensory testing, neurophysiological and psychological examination in patients with complex regional pain syndrome and hemisensory deficits. Pain 2001;93(3):279–93. 11514087 10.1016/S0304-3959(01)00332-3

[287] Park HY , Jang YE, Oh S, Lee PB. Psychological characteristics in patients with chronic complex regional pain syndrome: comparisons with patients with major depressive disorder and other types of chronic pain. J Pain Res 2020;13:389–98. 32104060 10.2147/JPR.S230394 PMC7026116

[288] Geertzen JHB , de Bruijn H, de Bruijn-Kofman AT, Arendzen JH. Reflex sympathetic dystrophy: Early treatment and psychological aspects. Arch Phys Med Rehabil 1994;75(4):442–6. 8172505 10.1016/0003-9993(94)90169-4

[289] Hardy M , Merritt W. Psychological evaluation and pain assessment in patients with reflex sympathetic dystrophy. J Hand Ther 1988;1(4):155–64.

[290] Bruehl S , Husfeldt B, Lubenow t, Nath H, Ivankovich AD. Psychological differences between reflex sympathetic dystrophy and non-RSD chronic pain patients. Pain 1996;67(1):107–14. 8895237 10.1016/0304-3959(96)81973-7

[291] Feldman SI , Downey G, Schaffer-Neitz R. Pain, negative mood, and perceived support in chronic pain patients: A daily diary study of people with reflex sympathetic dystrophy syndrome. J Consult Clin Psychol 1999;67(5):776–85. 10535244 10.1037//0022-006x.67.5.776

[292] Burns JW , Gerhart JI, Bruehl S, et al Anger arousal and behavioral anger regulation in everyday life among patients with chronic low back pain: Relationships to patient pain and function. Health Psychol 2015;34(5):547–55. 25110843 10.1037/hea0000091 PMC4513650

[293] Ciccone DS , Bandilla EB, Wu W. Psychological dysfunction in patients with reflex sympathetic dystrophy. Pain 1997;71:323–33. 9231876 10.1016/s0304-3959(97)00009-2

[294] DeGood DE , Cundiff GW, Adams LE, Shutty MS Jr. A psychosocial and behavioral comparison of reflex sympathetic dystrophy, low back pain, and headache patients. Pain 1993;54(3):317–22. 8233546 10.1016/0304-3959(93)90031-J

[295] Haddox JD , Abram SE, Hopwood MH. Comparison of psychometric data in RSD and radiculopathy. Reg Anesth 1988;13:27.

[296] Bruehl S , Chung OY, Burns JW. Differential effects of expressive anger regulation on chronic pain intensity in CRPS and non-CRPS limb pain patients. Pain 2003;104(3):647–54. 12927637 10.1016/S0304-3959(03)00135-0

[297] Bean DJ , Johnson MH, Kydd RR. Relationships between psychological factors, pain, and disability in complex regional pain syndrome and low back pain. Clin J Pain 2014;30(8):647–‐53. 24135903 10.1097/AJP.0000000000000007

[298] Margalit D , Ben Har L, Brill S, Vatine JJ. Complex regional pain syndrome, alexithymia, and psychological distress. J Psychosom Res 2014;77(4):273–‐7. 25280824 10.1016/j.jpsychores.2014.07.005

[299] Breimhorst M , Dellen C, Wittayer M, Rebhorn C, Drummond PD, Birklein F. Mental load during cognitive performance in complex regional pain syndrome I. Eur J Pain 2018;22(7):1343–‐50. 29635839 10.1002/ejp.1223

[300] Hartrick CT , Kovan JP, Naismith P. Outcome prediction following sympathetic block for complex regional pain syndrome. Pain Pract 2004;4(3):222–8. 17173603 10.1111/j.1533-2500.2004.04306.x

[301] Schurmann M , Gradl G, Andress HJ, Furst H, Schildberg FW. Assessment of peripheral sympathetic nervous function for diagnosing early post-traumatic complex regional pain syndrome type I. Pain 1999;80(1-2):149–59. 10204727 10.1016/s0304-3959(98)00198-5

[302] Kemler MA , de Vet HC. Health-related quality of life in chronic refractory reflex sympathetic dystrophy (complex regional pain syndrome type I). J Pain Symptom Manage 2000;20(1):68–76. 10946171 10.1016/s0885-3924(00)00170-6

[303] Geertzen JH , Dijkstra PU, Groothoff JW, ten Duis HJ, Eisma WH. Reflex sympathetic dystrophy of the upper extremity–a 5.5-year follow-up. Part I. Impairments and perceived disability. Acta Orthop Scand Suppl 1998;279:12–8. 9614810

[304] Weber M , Birklein F, Neundorfer B, Schmelz M. Facilitated neurogenic inflammation in complex regional pain syndrome. Pain 2001;91(3):251–7. 11275381 10.1016/S0304-3959(00)00445-0

[305] Bruehl S. Do psychological factors play a role in the onset and maintenance of CRPS? In: Harden R, Baron R, Janig W, eds. Complex Regional Pain Syndrome. Seattle, WA: IASP Press; 2001:279–90.

[306] Bean DJ , Johnson MH, Heiss-Dunlop W, Lee AC, Kydd RR. Do psychological factors influence recovery from complex regional pain syndrome type 1? A prospective study. Pain 2015;156(11):2310–8. 26133727 10.1097/j.pain.0000000000000282

[307] Cho S , McCracken LM, Heiby EM, Moon DE, Lee JH. Pain acceptance-based coping in complex regional pain syndrome Type I: Daily relations with pain intensity, activity, and mood. J Behav Med 2013;36(5):531–8. 22854886 10.1007/s10865-012-9448-7

[308] Blanchard EB. The use of temperature biofeedback in the treatment of chronic pain due to causalgia. Biofeedback Self Regul 1979;4(2):183–8. 476193 10.1007/BF01007112

[309] Alioto JT. Behavioral treatment of reflex sympathetic dystrophy. Psychosomatics 1981;22(6):539–40. 6265965 10.1016/S0033-3182(81)73501-1

[310] Barowsky EI , Zweig JB, Moskowitz J. Thermal biofeedback in the treatment of symptoms associated with reflex sympathetic dystrophy. J Child Neurol 1987;2(3):229–32. 3611636 10.1177/088307388700200311

[311] Kawano M , Matsuoka M, Kurokawa T, Tomita S, Mizuno Y, Ueda K. Autogenic training as an effective treatment for reflex neurovascular dystrophy: A case report. Acta Paediatr Jpn 1989;31(4):500–3. 2514577 10.1111/j.1442-200x.1989.tb01341.x

[312] Wesdock KA , Stanton RP, Singsen BH. Reflex sympathetic dystrophy in children. A physical therapy approach. Arthritis Care Res 1991;4(1):32–8. 11188585 10.1002/art.1790040107

[313] Gainer MJ. Hypnotherapy for reflex sympathetic dystrophy. Am J Clin Hypn 1992;34(4):227–32. 1349789 10.1080/00029157.1992.10402852

[314] Wilder RT , Berde CB, Wolohan M, Vieyra MA, Masek BJ, Micheli LJ. Reflex sympathetic dystrophy in children: Clinical characteristics and follow-up of seventy patients. J Bone Joint Surg Am 1992;74(6):910–9. 1634582

[315] Fialka V , Korpan M, Saradeth T, et al Autogenic training for reflex sympathetic dystrophy: A pilot study. Complement Ther Med 1996;4(2):103–5.

[316] Oerlemans HM , Oostendorp RA, de Boo T, van der Laan L, Severens JL, Goris JA. Adjuvant physical therapy versus occupational therapy in patients with reflex sympathetic dystrophy/complex regional pain syndrome type I. Arch Phys Med Rehabil 2000;81(1):49–56. 10638876

[317] Singh G , Willen SN, Boswell MV, Janata JW, Chelimsky TC. The value of interdisciplinary pain management in complex regional pain syndrome type I: A prospective outcome study. Pain Physician 2004;7:203–9. 16868593

[318] Bartur G , Vatine JJ, Raphaely-Beer N, Peleg S, Katz-Leurer M. Heart rate autonomic regulation system at rest and during paced breathing among patients with CRPS as compared to age-matched healthy controls. Pain Med 2014;15(9):1569–‐74. 25060074 10.1111/pme.12449

[319] Barnhoorn KJ , van de Meent H, van Dongen RT, et al Pain exposure physical therapy (PEPT) compared to conventional treatment in complex regional pain syndrome type 1: A randomised controlled trial. BMJ Open 2015;5(12):e008283. 10.1136/bmjopen-2015-008283 PMC4679993 26628523

[320] den Hollander M , Goossens M, de Jong J, et al Expose or protect? A randomized controlled trial of exposure in vivo vs pain-contingent treatment as usual in patients with complex regional pain syndrome type 1. Pain 2016;157(10):2318–29. 27429174 10.1097/j.pain.0000000000000651

[321] Solcà M , Ronchi R, Bello-Ruiz J, et al Heartbeat-enhanced immersive virtual reality to treat complex regional pain syndrome. Neurology 2018;91(5):e479–89. 29980635 10.1212/WNL.0000000000005905

[322] den Hollander MD , de Jong J, Onghena P, Vlaeyen JWS. Generalization of exposure in vivo in Complex Regional Pain Syndrome type I. Behav Res Ther 2020;124:103511. 31865235 10.1016/j.brat.2019.103511

[323] Vowles KE , Pielech M, Edwards KA, McEntee ML, Bailey RW. A comparative meta-analysis of unidisciplinary psychology and interdisciplinary treatment outcomes following acceptance and commitment therapy for adults with chronic pain. J Pain 2021;21(5–6):529–45. 10.1016/j.jpain.2019.10.004 PMC7477894 31683020

[324] Elomaa M , Hotta J, de C Williams AC, et al Symptom reduction and improved function in chronic CRPS type 1 after 12-week integrated, interdisciplinary therapy. Scand J Pain 2019;19(2):257–70. 30789827 10.1515/sjpain-2018-0098

[325] Wetering EJ , Lemmens KM, Nieboer AP, Huijsman R. Cognitive and behavioral interventions for the management of chronic neuropathic pain in adults–a systematic review. Eur J Pain 2010;14(7):670–81. 20096614 10.1016/j.ejpain.2009.11.010

[326] Jensen IB , Bergstrom G, Ljungquist T, Bodin L, Nygren AL. A randomized controlled component analysis of a behavioral medicine rehabilitation program for chronic spinal pain: Are the effects dependent on gender? Pain 2001;91(1-2):65–78. 11240079 10.1016/s0304-3959(00)00420-6

[327] Carlson CR , Hoyle RH. Efficacy of abbreviated progressive muscle relaxation training: A quantitative review of behavioral medicine research. J Consult Clin Psy 1993;61(6):1059–67. 10.1037//0022-006x.61.6.1059 8113484

[328] Stetter F , Kupper S. Autogenic training: A meta-analysis of clinical outcome studies. Appl Psychophysiol Biofeedback 2002;27(1):45–98. 12001885 10.1023/a:1014576505223

[329] Holroyd KA , Penzien DB. Pharmacological versus non-pharmacological prophylaxis of recurrent migraine headache: A meta-analytic review of clinical trials. Pain 1990;42(1):1–13. 2146583 10.1016/0304-3959(90)91085-W

[330] Crider AB , Glaros AG. A meta-analysis of EMG biofeedback treatment of temporomandibular disorders. J Orofac Pain 1999;13(1):29–37. 10425966

[331] Malone MD , Strube MJ, Scogin FR. Meta-analysis of non-medical treatments for chronic pain. Pain 1988;34(3):231–44. 3054729 10.1016/0304-3959(88)90118-2

[332] Bogaards MC , ter Kuile MM. Treatment of recurrent tension headache: A meta-analytic review. Clin J Pain 1994;10(3):174–90. 7833575 10.1097/00002508-199409000-00003

[333] Morley S , Eccleston C, Williams A. Systematic review and meta-analysis of randomized controlled trials of cognitive behaviour therapy and behaviour therapy for chronic pain in adults, excluding headache. Pain 1999;80(1-2):1–13. 10204712 10.1016/s0304-3959(98)00255-3

[334] Rossy LA , Buckelew SP, Dorr N, et al A meta-analysis of fibromyalgia treatment interventions. Ann Behav Med 1999;21(2):180–91. 10499139 10.1007/BF02908299

[335] Astin JA , Beckner W, Soeken K, Hochberg MC, Berman B. Psychological interventions for rheumatoid arthritis: A meta-analysis of randomized controlled trials. Arthritis Rheum 2002;47(3):291–302. 12115160 10.1002/art.10416

[336] Sim J , Adams N. Systematic review of randomized controlled trials of nonpharmacological interventions for fibromyalgia. Clin J Pain 2002;18(5):324–36. 12218504 10.1097/00002508-200209000-00008

[337] Devine EC. Meta-analysis of the effect of psychoeducational interventions on pain in adults with cancer. Oncol Nurs Forum 2003;30(1):75–89. 12515986 10.1188/03.ONF.75-89

[338] Donahue ML , Dunne EM, Gathright EC, et al Complementary and integrative health approaches to manage chronic pain in U.S. military populations: Results from a systematic review and meta-analysis, 1985-2019 [published online ahead of print, 2020 Mar 5]. Psychol Serv 2020;18(3):295–309. 32134305 10.1037/ser0000417 PMC7483193

[339] Khoo EL , Small R, Cheng W, et al Comparative evaluation of group-based mindfulness-based stress reduction and cognitive behavioural therapy for the treatment and management of chronic pain: A systematic review and network meta-analysis. Evid Based Ment Health 2019;22(1):26–35. 30705039 10.1136/ebmental-2018-300062 PMC10270397

[340] Hilton L , Hempel S, Ewing BA, et al Mindfulness meditation for chronic pain: systematic review and meta-analysis. Ann Behav Med 2017;51(2):199–‐213. 27658913 10.1007/s12160-016-9844-2 PMC5368208

[341] Hughes LS , Clark J, Colclough JA, Dale E, McMillan D. Acceptance and Commitment Therapy (ACT) for chronic pain: A systematic review and meta-analyses. Clin J Pain 2017;33(6):552–68. 27479642 10.1097/AJP.0000000000000425

[342] Eccleston C , Morley S, Williams A, Yorke L, Mastroyannopoulou K. Systematic review of randomized controlled trials of psychological therapy for chronic pain in children and adolescents, with a subset meta-analysis of pain relief. Pain 2002;99(1-2):157–65. 12237193 10.1016/s0304-3959(02)00072-6

[343] Grieve S , Adams J, McCabe C. ‘What I Really Needed Was the Truth’. Exploring the information needs of people with complex regional pain syndrome. Musculoskelet Care 2016;14(1):15–25. 10.1002/msc.1107 26076593

[344] Vlaeyen JW , Seelen HA, Peters M, et al Fear of movement/(re)injury and muscular reactivity in chronic low back pain patients: An experimental investigation. Pain 1999;82(3):297–304. 10488681 10.1016/S0304-3959(99)00054-8

[345] Asmundson GJ , Norton PJ, Norton GR. Beyond pain: The role of fear and avoidance in chronicity. Clin Psychol Rev 1999;19(1):97–119. 9987586 10.1016/s0272-7358(98)00034-8

[346] Haythornthwaite JA , Clark MR, Pappagallo M, Raja SN. Pain coping strategies play a role in the persistence of pain in post-herpetic neuralgia. Pain 2003;106(3):453–60. 14659529 10.1016/j.pain.2003.09.009

[347] Rodham K , McCabe C, Blake D. Seeking support: An interpretative phenomenological analysis of an Internet message board for people with Complex Regional Pain Syndrome. Psychol Health 2009;24(6):619–34. 20205016 10.1080/08870440802563245

[348] Andrasik F , Holroyd KA. Specific and nonspecific effects in the biofeedback treatment of tension headache: 3-year follow-up. J Consult Clin Psychol 1983;51(4):634–6. 6619376 10.1037//0022-006x.51.4.634

[349] Vowles KE , Sowden G, Ashworth J. A comprehensive examination of the model underlying acceptance and commitment therapy for chronic pain. Behav Ther 2014;45(3):390–‐401. 24680233 10.1016/j.beth.2013.12.009

[350] Blake H. Strain and psychological distress among informal supporters of reflex sympathetic dystrophy patients. Disabil Rehabil 2000;22(18):827–32. 11197519 10.1080/09638280050207875

[351] Deer TR , Levy RM, Kramer J, et al Dorsal root ganglion stimulation yielded higher treatment success rate for complex regional pain syndrome and causalgia at 3 and 12 months: A randomized comparative trial. Pain 2017;158(4):669–81. 28030470 10.1097/j.pain.0000000000000814 PMC5359787

[352] Deer TR , Pope JE, Lamer TJ, et al The Neuromodulation Appropriateness Consensus Committee on Best Practices for Dorsal Root Ganglion Stimulation. Neuromodulation 2019;22(1):1–35. 30246899 10.1111/ner.12845

[353] Shealy CN , Mortimer JT, Reswick JB. Electrical inhibition of pain by stimulation of the dorsal columns: Preliminary clinical report. Anesth Analg 1967;46(4):489–91. 4952225

[354] Janig W , Baron R. Complex regional pain syndrome: Mystery explained? Lancet Neurology 2003;2(11):687–97. 14572737 10.1016/s1474-4422(03)00557-x

[355] Livingstone JA , Atkins RM. Intravenous regional guanethidine blockade in the treatment of post-traumatic complex regional pain syndrome type 1 (algodystrophy) of the hand. J Bone Joint Surg Br 2002;84-B (3):380–6. 10.1302/0301-620x.84b3.11901 12002497

[356] Bruehl S , Gamazon ER, Van de Ven T, et al DNA methylation profiles are associated with complex regional pain syndrome after traumatic injury. Pain 2019;160(10):2328–37. 31145213 10.1097/j.pain.0000000000001624 PMC7473388

[357] Janig W , Habler H. Sympathetic nervous system: Contribution to chronic pain. Prog Brain Res 2000;129:451–68. 11098710

[358] Price D , Long S, Wilsey B, Rafii A. Analysis of peak magnitude and duration of analgesia produced by local anesthetics injected into sympathetic ganglia of complex regional pain syndrome patients. Clin J Pain 1998;14(3):216–26. 9758071 10.1097/00002508-199809000-00008

[359] Burton A , Conroy B, Sims S, Solanki D, Williams C. Complex regional pain syndrome type II as a complication of subclavian line insertion (letter). Anesthesiology 1998;89:804. 9743433 10.1097/00000542-199809000-00053

[360] Arner S. Intravenous phentolamine test: Diagnostic and prognostic use in reflex sympathetic dystrophy. Pain 1991;46:17–22. 1896205 10.1016/0304-3959(91)90028-V

[361] Hord AH , Rooks MD, Stephens BO, Rogers HG, Fleming LL. Intravenous regional bretylium and lidocaine for treatment of reflex sympathetic dystrophy: A randomized, double-blind study. Anesth Analg 1992;74(6):818–21. 1595913 10.1213/00000539-199206000-00007

[362] Reuben S , Sklar J. Intravenous regional analgesia with clonidine in the management of complex regional pain syndrome of the knee. J Clin Anesth 2002;14(2):87–91. 11943518 10.1016/s0952-8180(01)00346-4

[363] Blanchard J , Ramamurthy S, Walsh N, Hoffman J, Schoenfeld L. Intravenous regional sympatholysis: A double-blind comparison of guanethidine, reserpine, and normal saline. J Pain Sympt Management 1990;5(6):357–61. 10.1016/0885-3924(90)90030-n 2269803

[364] Boas R. Sympathetic nerve blocks: In search of a role. Reg Anesth Pain Med 1998;23(3):292–305. 9613543 10.1016/s1098-7339(98)90058-x

[365] Stanton-Hicks M. Complex regional pain syndrome. Anesthesiol Clin North America 2003;21(4):733–44. 14719716 10.1016/s0889-8537(03)00084-1

[366] Schurmann M , Gradl G, Wizgal I. Clinical and physiologic evaluation of stellate ganglion blockade for complex regional pain syndrome type I. Clin J Pain 2001;17:94–100. 11289093 10.1097/00002508-200103000-00012

[367] Cepeda M , Lau J, Carr D. Defining the therapeutic role of local anesthetic sympathetic blockade in complex regional pain syndrome: A narrative and systematic review. Clin J Pain 2002;18(4):216–33. 12131063 10.1097/00002508-200207000-00002

[368] Raja SN , Treede RD, Davis KD, Campbell JN. Systemic alpha-adrenergic blockade with phentolamine: A diagnostic test for sympathetically maintained pain. Anesthesiology 1991;74(4):691–8. 1848966 10.1097/00000542-199104000-00012

[369] Malmqvist EL , Bengtsson M, Sorensen J. Efficacy of stellate ganglion block: A clinical study with bupivacaine. Reg Anesth 1992;17(6):340–7. 1363053

[370] Raj PP , Montgomery SJ, Nettles D, Jenkins MT. Infraclavicular brachial plexus block–a new approach. Anesth Analg 1973;52(6):897–904. 4796563

[371] Raj P. Nerve blocks: Continuous regional analgesia. In: Raj P, ed. *Practical Management of Pain*, Vol. 3. St Louis: Mosby; 2000. 710–22.

[372] Wang L , Chen H, Chang P, Kang F, Tsai Y. Axillary brachial plexus block with patient controlled analgesia for complex regional pain syndrome type I: A case report. Reg Anesth Pain Med 2001;26(1):68–71. 11172515 10.1053/rapm.2001.9879

[373] Cooper DE , DeLee JC, Ramamurthy S. Reflex sympathetic dystrophy of the knee. Treatment using continuous epidural anesthesia. J Bone Joint Surg Am 1989;71(3):365–9. 2925709

[374] Koning H , Christiaans C, Overdijk G, Mackie D. Cervical epidural blockade and reflex sympathetic dystrophy. Pain Clinic 1995;8:239–44.

[375] Buchneit T , Crews J. Lateral cervical epidural catheter placement for continuous unilateral upper extremity analgesia and sympathetic block. Reg Anesth Pain Med 2000;25(3):313–7. 10834791 10.1016/s1098-7339(00)90019-1

[376] Du Pen SL , Peterson DG, Williams A, Bogosian AJ. Infection during chronic catheter epidural catheterization: Diagnosis and treatment. Anesthesiology 1990;73(5):905–9. 2240680 10.1097/00000542-199011000-00018

[377] Lundborg C , Dahm P, Nitescu P, Appelgren L, Curelaru I. Clinical experience using intrathecal bupivacaine infusion in three patients with complex regional pain syndrome type I. Acta Anaesthesiol Scand 1999;43(6):667–78. 10408823 10.1034/j.1399-6576.1999.430613.x

[378] Evans J. Sympathectomy for reflex sympathetic dystrophy: Report of 29 cases. JAMA 1946;132(11):620–3. 10.1001/jama.1946.02870460010003 21001604

[379] Kim K , DeSalles A, Johnson J, Ahn S. Sympathectomy: Open and thoracoscopic. In: Burchiel K, ed. *Surgical Management of Pain*. New York: Thieme Publishers; 2002:688–700.

[380] Mockus MB , Rutherford RB, Rosales C, Pearce WH. Sympathectomy for causalgia. Patient selection and long-term results. Arch Surg 1987;122(6):668–72. 3579581 10.1001/archsurg.1987.01400180050009

[381] Wilkinson H. Percutaneous radiofrequency upper thoracic sympathectomy. Neurosurgery 1996;38(4):715–25. 8692390

[382] Ghosh P , Gungor S. Utilization of Concurrent Dorsal Root Ganglion Stimulation and Dorsal Column Spinal Cord Stimulation in Complex Regional Pain Syndrome [Mar 11]. Neuromodulation 2020;10:1–5. 10.1111/ner.13144 32162402

[383] Hunter CW , Sayed D, Lubenow T, et al DRG FOCUS: A Multicenter Study Evaluating Dorsal Root Ganglion Stimulation and Predictors for Trial Success. Neuromodulation 2019;22(1):61–79. 30085382 10.1111/ner.12796

[384] Kemler MA , Barendse GAM, van Kleef M, et al Spinal cord stimulation in patients with chronic reflex sympathetic dystrophy. N Engl J Med 2000;343(9):618–24. 10965008 10.1056/NEJM200008313430904

[385] Kemler M , De Vet H, Barendse G, Van Den Wildenberg F, van Cleef M. The effect of spinal cord stimulation in patients with chronic reflex sympathetic dystrophy: Two years follow-up of the randomized controlled trial. Ann Neurol 2004;55(1):13–8. 14705107 10.1002/ana.10996

[386] Forouzanfar T , Kemler M, Weber W, Kessels A, van Cleef M. Spinal cord stimulation in complex regional pain syndrome: Cervical and lumbar devices are comparably effective. Br J Anaesth 2004;92(3):348–53. 14742334 10.1093/bja/aeh072

[387] Kriek N , Schreurs MWJ, Groeneweg JG, et al Spinal cord stimulation in patients with complex regional pain syndrome: A possible target for immunomodulation? Neuromodulation 2018;21(1):77–86. 29064599 10.1111/ner.12704

[388] van Eijs F , Smits H, Geurts JW, et al Brush-evoked allodynia predicts outcome of spinal cord stimulation in complex regional pain syndrome type 1. Eur J Pain 2010;14(2):164–9. 19942463 10.1016/j.ejpain.2009.10.009

[389] Mekhail N , Deer TR, Kramer J, et al Paresthesia-free dorsal root ganglion stimulation: An ACCURATE study sub-analysis. Neuromodulation 2020;23(2):185–95. 30861286 10.1111/ner.12942

[390] Dworkin RH , O'Connor AB, Kent J, et al Interventional management of neuropathic pain: NeuPSIG recommendations. Pain 2013;154(11):2249–61. 23748119 10.1016/j.pain.2013.06.004 PMC4484720

[391] Huygen FJPM , Kallewaard JW, Nijhuis H, et al Effectiveness and safety of dorsal root ganglion stimulation for the treatment of chronic pain: A pooled analysis. Neuromodulation 2020;23(2):213–21. 31730273 10.1111/ner.13074 PMC7079258

[392] Hannington-Kiff JG. Intravenous regional sympathetic block with guanethidine. Lancet 1974;1(7865):1019–20. 4133701 10.1016/s0140-6736(74)90418-8

[393] Ramamurthy S , Hoffman J, Group GS. Intravenous regional guanethidine in the treatment of reflex sympathetic dystrophy/causalgia: A randomized double-blind study. Anesth Analg 1995;81(4):718–23. 7574000 10.1097/00000539-199510000-00011

[394] Jadad AR , Carroll D, Glynn CJ, McQuay HJ. Intravenous regional sympathetic dystrophy: A systemic review and a randomized, double-blind crossover study. J Pain Symptom Manag 1995;10(1):13–20. 10.1016/0885-3924(94)00064-R 7536227

[395] Rocco A , Kaul A, Reisman R, Gallo J, Lief P. A comparison of regional intravenous guanethidine and reserpine in reflex sympathetic dystrophy. A controlled, randomized, double-blind, crossover study. Clin J Pain 1989;5(3):205–9. 2520406 10.1097/00002508-198909000-00002

[396] Leis S , Weber M, Schmelz M, Birklein F. Facilitated neurogenic inflammation in unaffected limbs of patients with complex regional pain syndrome. Neurosci Lett 2004;359(3):163–6. 15050689 10.1016/j.neulet.2004.02.025

[397] Verdugo R , Ochoa JL. Sympathetically maintained pain I. Phentolamine block questions the concept. Neurology 1994;44(6):1003–10. 8208390 10.1212/wnl.44.6.1003

[398] Wallace M , Ridgeway B, Leung A, Gerayli A, Yaksh T. Concentration-effect relationship of intravenous lidocaine on the allodynia of complex regional pain syndrome types I and II. Anesthesiology 2000;92(1):75–83. 10638902 10.1097/00000542-200001000-00017

[399] Ostergard T , Munyon C, Miller JP. Motor cortex stimulation for chronic pain. Neurosurg Clin N Am 2014;25(4):693–8. 25240657 10.1016/j.nec.2014.06.004

[400] Taylor RS. Spinal cord stimulation in complex regional pain syndrome and refractory neuropathic back and leg pain/failed back surgery syndrome: results of a systematic review and meta-analysis. J Pain Symptom Manage. 2006 Apr;31(4 Suppl):S13–9. doi: 10.1016/j.jpainsymman.2005.12.010. PMID: 16647590. 10.1016/j.jpainsymman.2005.12.010 16647590

[401] Kemler MA, de Vet HC, Barendse GA, van den Wildenberg FA, van Kleef M. Effect of spinal cord stimulation for chronic complex regional pain syndrome Type I: five-year final follow-up of patients in a randomized controlled trial. J Neurosurg. 2008 Feb;108(2):292–8. doi: 10.3171/JNS/2008/108/2/0292. PMID: 18240925. 10.3171/JNS/2008/108/2/0292 18240925

[402] Kriek N, Groeneweg JG, Stronks DL, de Ridder D, Huygen FJ. Preferred frequencies and waveforms for spinal cord stimulation in patients with complex regional pain syndrome: A multicentre, double-blind, randomized and placebo-controlled crossover trial. Eur J Pain. 2017 Mar;21(3):507–519. doi: 10.1002/ejp.944. Epub 2016 Oct 7. PMID: 27714945. 10.1002/ejp.944 27714945

[403] Deer TR, Levy RM, Kramer J, Poree L, Amirdelfan K, Grigsby E, Staats P, Burgher AH, Scowcroft J, Golovac S, Kapural L, Paicius R, Pope JE, Samuel S, Porter McRoberts W, Schaufele M, Burton AW, Raza A, Agnesi F, Mekhail N. Comparison of Paresthesia Coverage of Patient's Pain: Dorsal Root Ganglion vs. Spinal Cord Stimulation. An ACCURATE Study Sub-Analysis. Neuromodulation. 2019 Dec;22(8):930–936. doi: 10.1111/ner.12920. Epub 2019 Jan 9. PMID: 30624003. 10.1111/ner.12920 30624003

[404] Bennett DS, Aló KM, Oakley J, Feler CA. Spinal Cord Stimulation for Complex Regional Pain Syndrome I [RSD]: a Retrospective Multicenter Experience from 1995 to 1998 of 101 Patients. Neuromodulation. 1999 Jul;2(3):202–10. doi: 10.1046/j.1525-1403.1999.00202.x. PMID: 22151209. 10.1046/j.1525-1403.1999.00202.x 22151209

[405] Kemler MA, Barendse GA, Van Kleef M, Van Den Wildenberg FA, Weber WE. Electrical spinal cord stimulation in reflex sympathetic dystrophy: retrospective analysis of 23 patients. J Neurosurg. 1999 Jan;90(1 Suppl):79–83. doi: 10.3171/spi.1999.90.1.0079. PMID: 10413130. 10.3171/spi.1999.90.1.0079 10413130

[406] Kumar K, Nath R, Toth C. Spinal cord stimulation is effective in the management of reflex sympathetic dystrophy. Neurosurgery 1997;40:503–9. 10.1097/00006123-199703000-00014 9055289

